# Do mindfulness-based programmes improve the cognitive skills, behaviour and mental health of children and adolescents? An updated meta-analysis of randomised controlled trials

**DOI:** 10.1136/ebmental-2022-300464

**Published:** 2022-07-07

**Authors:** Darren Dunning, Kate Tudor, Lucy Radley, Nicola Dalrymple, Julia Funk, Maris Vainre, Tamsin Ford, Jesus Montero-Marin, Willem Kuyken, Tim Dalgleish

**Affiliations:** 1 Medical Research Council Cognition and Brain Sciences Unit, University of Cambridge, Cambridge, UK; 2 Department of Psychiatry, University of Oxford, Oxford, UK; 3 Institute of Clinical Psychology and Psychotherapy, Ludwig-Maximilians-University of Munich, Munich, Germany; 4 Department of Psychiatry, University of Cambridge, Cambridge, UK; 5 Teaching, Research and Inovation Unit, Parc Sanitari Sant Joan de Deu, Saint Boi de Llobregat, Spain; 6 Cambridgeshire and Peterborough NHS Foundation Trust, Fulbourn, UK

**Keywords:** Child & adolescent psychiatry

## Abstract

**Question:**

Mindfulness-based programmes (MBPs) are an increasingly popular approach to improving mental health in young people. Our previous meta-analysis suggested that MBPs show promising effectiveness, but highlighted a lack of high-quality, adequately powered randomised controlled trials (RCTs). This updated meta-analysis assesses the-state-of the-art of MBPs for young people in light of new studies. It explores MBP’s effectiveness in active vs passive controls; selective versus universal interventions; and studies that included follow-up.

**Study selection and analysis:**

We searched for published and unpublished RCTs of MBPs with young people (<19 years) in PubMed Central, PsycINFO, Web of Science, EMBASE, ICTRP, ClinicalTrials.gov, EThOS, EBSCO and Google Scholar. Random-effects meta-analyses were conducted, and standardised mean differences (Cohen’s d) were calculated.

**Findings:**

Sixty-six RCTs, involving 20 138 participants (9552 receiving an MBP and 10 586 controls), were identified. Compared with passive controls, MBPs were effective in improving anxiety/stress, attention, executive functioning, and negative and social behaviour (d from 0.12 to 0.35). Compared against active controls, MBPs were more effective in reducing anxiety/stress and improving mindfulness (d=0.11 and 0.24, respectively). In studies with a follow-up, there were no significant positive effects of MBPs. No consistent pattern favoured MBPs as a universal versus selective intervention.

**Conclusions:**

The enthusiasm for MBPs in youth has arguably run ahead of the evidence. While MBPs show promising results for some outcomes, in general, the evidence is of low quality and inconclusive. We discuss a conceptual model and the theory-driven innovation required to realise the potential of MBPs in supporting youth mental health.

**WHAT IS ALREADY KNOWN ON THIS TOPIC**

- Our previous meta-analysis of randomised controlled trials (RCTs) of mindfulness-based programmes (MBPs) in children and young people provided preliminary support from 33 trials (n=3666) for MBPs improving mindfulness skills and symptoms of depression and anxiety against both active and passive control comparators. There was also evidence of improvements in cognitive skills when MBPs were compared to passive controls. However, RCTs were relatively small and follow-up data were sparse.

**WHAT THIS STUDY ADDS**

- This study updates the previous meta-analysis to now include data from 66 RCTs (n=20 168), including a number of recent adequately powered studies and studies with follow-ups. MBPs continued to improve mindfulness and symptoms of anxiety (but no longer depression) relative to active controls, and to improve cognitive skills relative to passive controls. New analyses revealed no beneficial effects of MBPs for wellbeing and no evidence of sustained benefits at follow-up. In addition, despite the influx of new RCTs, study quality was typically low and heterogeneity was high.

**HOW THIS STUDY MIGHT AFFECT RESEARCH, PRACTICE OR POLICY**

- The next generation of research needs to be theoretically informed and designed and adequately powered to answer questions of what works, for whom and how, as well as considering key contextual and implementation factors and elucidating whether MBPs can deliver sustained benefits.

## Background

Mindfulness-based programmes (MBPs) seek to improve cognitive, emotional and behavioural outcomes for young people. Our previous meta-analysis of randomised controlled trials (RCTs) suggested that, overall, MBPs significantly improved negative behaviour, attention, executive functions, anxiety/stress and depression. However, when comparing MBPs against active controls, effects were confined to anxiety/stress and depression.^1^ We also highlighted wider issues, including a lack of conceptual specification of both the target population and the nature of the prevention/intervention (universal vs indicated prevention, vs treatment),^1^ a lack of higher quality and adequately powered RCTs with adequate follow-up, and considerable heterogeneity and publication bias.

Since this previous meta-analysis, many more studies using MBPs with young people have been published, including fully powered RCTs.^2–4^ In addition, a number of studies now include longer follow-ups allowing us to gauge the sustainability of positive effects of MBPs with young people. Finally, more studies specify the target population (eg, age/developmental stage), whether they use selective/indicated or universal interventions, and also important dimensions of the intervention, such as dose received.

## Objective

The aim of this meta-analysis is to assess the updated evidence for MBPs improving the behaviour, mental health and cognitive outcomes of children and adolescents. Specifically, we evaluate MBPs against passive versus active controls, as selective/indicated (hereafter refered to as selective) vs universal programmes, and at postintervention and follow-up (calculated as the time lapsed following the post-assessments conducted immediately following cessation of the MBP). We further examine whether risk-of-bias, dose-of-training and age moderate outcomes.

### Study selection and analysis

#### Search strategy and inclusion criteria

The literature search followed Dunning *et al* 1 (2019; PROSPERO #42016038364). Searches for published and unpublished articles were carried out by three authors (DD, LR, ND) from January 2017 (the end date of our search for the previous meta-analysis) to January 2022, using keyword searches and titles in PubMed Central, PsycINFO, Web of Science, EMBASE and Google Scholar, using the terms “mindful*” AND “child*” OR “school” OR “adolescen*” OR “youth”. Additional literature searches of ICTRP, ClinicalTrials.gov, EThOS and EBSCO were carried out in April 2022 (DD, JM-M) using the same search terms. Reference lists of included studies and reviews were also searched. Searches were collated, duplicates removed and titles/abstracts of the remaining studies reviewed. Where the title/abstract suggested that the study may be appropriate, then the full- text was evaluated against the following inclusion criteria:

1. Study design: MBP versus a control condition with random assignment.
2. Participants: aged <18 years.
3. Intervention:
  - The core of the MBP comprised the essential elements from^5 5^ including: Present-moment-focus and decentring; Fostering attentional and behavioural self-regulation; Sustained mindfulness practice.
  - MBP comprised more than one session, delivered face to face by a trained mindfulness teacher.
  - Mindfulness training was the central intervention component—that is, not combined with another activity (eg, mindful yoga) or a subcomponent of a broader complex intervention (eg, acceptance commitment therapy).
4. Outcome variables: included either a measure of depression, anxiety/stress, well-being, mindfulness, negative behaviour, social behaviour or executive functioning, with quantitative data from which standardised mean difference(s) (SMDs) across conditions could be extracted. Studies were still collated if no outcomes were available in the published output. In such instances, the authors were contacted to establish if any outcome measures that were not reported in their manuscripts had been used. In the absence of relevant outcomes, papers were collated for inclusion in the review but excluded from quantitative analysis.

#### Data extraction and synthesis

For each study, we recorded: sample age, numbers of participants in each condition, type of control condition(s), targeted population, MBP dose (ie, sessions × session duration in minutes), type of MBP (eg, mindfulness-based stress reduction, dot.be), length of follow-up (if relevant) and outcome measures (see online supplemental table S1 for details of all studies included in quantitative analysis with references in online supplemental A; studies excluded from quantitative analysis can be found in online supplemental B). We made a number of decisions to combine data. For control condition, studies were split into ‘active’ or ‘passive’ groups. Active controls included attention placebos designed principally to account for non-specific factors, and active controls containing ingredients targeting change in one or more outcomes. Passive controls comprised no intervention, usual practice or wait list. For target population, interventions were classed as ‘selective’ or ‘universal’. Selective interventions included selective interventions targeting subpopulations at-risk of developing a disorder, based on known risk factors (eg, those with learning difficulties), and indicated interventions targeting individuals with detectable signs or symptoms of a disorder without being assessed as meeting diagnostic criteria (eg, individuals with symptoms of depression). Universal interventions were those targeting the whole population group (eg, whole school programmes).

10.1136/ebmental-2022-300464.supp1 Supplementary data

In line with our previous meta-analysis,^1^ outcomes were categorised as: anxiety/stress, depression, executive functioning, attention (a subset of executive functioning), mindfulness, negative behaviour (eg, aggression) and social behaviour (eg, empathy). Due to an increase in its use as an outcome (N=11 889 participants), we added a ‘well-being’ category as part of our update. Where studies used multiple measures in a given category, we chose the measure highest on a bespoke hierarchy based on the measure’s: theoretical fit with the construct it was designed to assess; frequency of use, especially in young populations; and psychometric properties, especially in young samples (see online supplemental C for a description of all measures used and online supplemental D the relevant hierarchies). Where measures included different raters (eg, Strengths and Difficulties Questionnaire), we reported parent/teacher rated outcomes.^6^

A mean of the SMDs for these measures was calculated on preintervention to postintervention effects (within 2 weeks of the end of the intervention). For studies using both active and passive controls, SMDs were calculated for each (ie, MBP vs active, MBP vs passive). For universal versus selective interventions, a mean of the SMDs was calculated across control conditions (active and passive) so that studies are represented only once in each outcome category. Outcomes were considered to be ‘improved’ when means were higher for the categories of mindfulness, executive control, attention, social behaviour and well-being, and lower for depression, anxiety/stress and negative behaviour. SMDs were calculated so that a positive SMD showed superiority of MBP over control condition. A full list of outcome measures, SMDs and outcome categories is in online supplemental table S2. The SMDs and categories for studies with follow-ups (between 1 and 40 months post-intervention and subsequent to a postintervention time point) are in online supplemental table S3.

#### Risk-of-bias and degree of evidence assessment

The Cochrane Collaboration’s Risk-of-Bias Tool V.2^7^ was used to assay the presence of biases that could lead to mis-estimation of intervention effects, see online supplemental E for full details.

The Grading of Recommendations, Assessment, Development and Evaluation (GRADE^8^) framework was used to evaluate the evidence for each outcome as ‘high’, ‘moderate’, ‘low’ or ‘very low’ based on: risk-of-bias, publication bias, inconsistency, imprecision, or indirectness. Two authors (JM-M, MV) independently assessed the evidence; in unclear cases a decision was made by a third party (DD) (see online supplemental F for full details).

#### Statistical analysis

The analysis plan followed our previous meta-analysis.^1^ Between-group SMDs (Cohen’s d^9^) were calculated based on the mean pre–post intervention change (or follow-up where relevant) in the MBP group minus the mean pre–post change in the control group, divided by the pooled preintervention SD^10 11^—chosen so the intervention does not influence the SD.^12^ Positive SMDs indicate the MBP benefitted more than the control and were interpreted as: d=0.20, a small effect; d=0.50, a moderate effect; and d=0.80, a large effect.^9^ Due to variation in studies (eg, universal or selective intervention, age-of-sample) random-effects models were used within the Comprehensive Meta-Analysis program V.3.3.70.^13^ 95% CIs were calculated for SMDs. Heterogeneity—the amount of variation in study outcomes between studies—was quantified using the Q statistic and I^2^ estimates. For I^2^, 0% equates to no heterogeneity, 25% to low, 50% to moderate and 75% to high.^14^

Separate random-effects meta-regression models examined the impact of three moderators: age-of-sample, to establish whether age determined who benefited most from mindfulness training; dose of mindfulness training, to explore whether duration of training influences results; and risk-of-bias, to examine whether level of study bias impacted findings. For studies that included a follow-up, we included time-since-post-assessment (between 1 and 40 months) as an additional potential moderator.

To investigate publication bias—the extent to which included studies were representative of the population of studies—we present Begg’s funnel plots and Egger’s regressions. This is important due to the tendency for journals to prefer to publish studies with positive rather than null findings.^15^ Missing studies were estimated using the trim-and-fill method.^16^

### Findings

**Figure 1:**
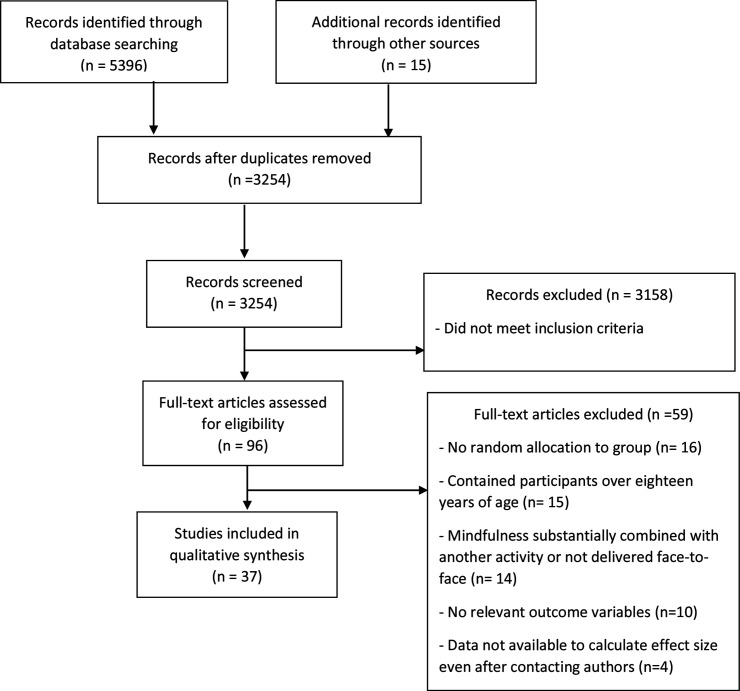
Flow chart of inclusion of studies.

Thirty-seven new studies met the inclusion criteria (figure 1) for quantitative analysis. Including the 29 eligible studies from our earlier review,^1^ there were 66 studies in total (N=20 138; n=9552 MBP, n=10 586 controls). Of these, 36 used an active control; 41 a passive control (NB: 7 studies used both active and passive control groups; 5 studies compared MBPs against 2 active control groups); 28 were selective interventions comprising seven indicated interventions targeting populations with detectable signs of symptoms and 21 selective interventions targeting at-risk populations; 38 were universal interventions; 23 included a follow-up assessment and 3 of these included two follow-up assessments.

Table 1 shows the results of the meta-analysis for all studies, studies with active controls, studies with passive controls, selective interventions and universal interventions, along with heterogeneity and publication bias analysis for each outcome category.

**Table 1:** Analysis of effects of MBPs, when compared with active/passive controls or delivered as a selective/universal intervention for selected outcomes

	k	Total n	Intervention effects					Heterogeneity			Publication bias (Egger)	
			Mean effect size (d)	SE	95% CI	z	P value	Q value	P value	I^2^	t	P value
Anxiety/stress												
All	36	11 530	0.14	0.04	(0.06 to 0.23)	3.25	<0.01	70.42	<0.01	50	4.08	<0.01
Active controls	17	1705	0.11	0.05	(0.02 to 0.21)	2.32	<0.05	10.17	0.86	0	1.49	0.16
Passive controls	21	9967	0.12	0.06	(−0.01 to 0.38)	2.05	<0.05	56.32	<0.01	64	2.51	<0.05
Selective intervention	18	1132	0.24	0.07	(0.11 to 0.37)	3.64	<0.01	18.98	0.33	10	0.55	0.59
Universal intervention	18	10 452	0.08	0.05	(−0.02 to 0.18)	1.64	0.1	34.86	<0.01	51	2.89	<0.05
Attention												
All	24	2920	0.21	0.07	(0.07 to 0.36)	2.89	<0.01	71.96	<0.01	68	1.3	0.21
Active controls	11	1503	0.04	0.05	(−0.06 to 0.14)	0.73	0.47	8.74	0.56	0	0.11	0.92
Passive controls	14	1435	0.35	0.12	(0.11 to 0.58)	2.91	<0.01	53.89	<0.01	76	0.9	0.38
Selective intervention	9	763	0.34	0.17	(−0.01 to 0.66)	2.02	<0.05	33.12	<0.01	76	0.75	0.48
Universal intervention	15	2142	0.16	0.08	(0.01 to 0.32)	2.06	<0.05	36.28	<0.01	61	0.74	0.47
Depression												
All	30	16 424	0.09	0.04	(0.02 to 0.17)	2.39	<0.05	70.93	<0.01	59	2.27	<0.05
Active controls	13	6002	0.1	0.07	(−0.04 to 0.23)	1.42	0.16	36.8	<0.01	67	1.1	0.3
Passive controls	19	12 802	0.06	0.04	(−0.02 to 0.13)	1.43	0.15	35.12	<0.05	46	1.87	0.08
Selective intervention	16	946	0.21	0.11	(0.004 to 0.42)	2	<0.05	34.6	<0.01	57	0.81	0.43
Universal intervention	14	15 478	0.05	0.04	(−0.02 to 0.12)	1.31	0.19	30.45	<0.01	57	1.29	0.22
Executive Functions												
All	36	11 618	0.25	0.06	(0.13 to 0.36)	4.22	<0.01	147.47	<0.01	76	3.49	<0.01
Active controls	14	1887	0.04	0.05	(−0.06 to 0.13)	0.76	0.45	13.25	0.43	2	0.86	0.41
Passive controls	25	9890	0.35	0.08	(0.19 to 0.52)	4.19	<0.01	140.9	<0.01	83	3.65	<0.01
Selective intervention	13	966	0.39	0.13	(0.14 to 0.65)	3.07	<0.01	38.35	<0.01	69	1.2	0.26
Universal intervention	23	10 652	0.19	0.06	(0.06 to 0.31)	2.89	<0.01	91.96	<0.01	76	2.31	<0.05
Mindfulness												
All	25	10 532	0.16	0.06	(0.04 to 0.29)	2.55	<0.05	84.65	<0.01	72	3.9	<0.01
Active controls	14	1628	0.24	0.01	(0.06 to 0.42)	2.61	<0.01	34.23	<0.01	62	1.1	0.29
Passive controls	12	8918	0.03	0.07	(-0.11 to 0.16)	0.4	0.69	25.85	<0.01	57	2.36	<0.05
Selective intervention	11	447	0.29	0.1	(0.09 to 0.50)	2.83	<0.01	12.19	0.27	18	0.04	0.97
Universal intervention	14	10 085	0.1	0.07	(−0.04 to 0.24)	1.44	0.15	58.24	<0.01	78	2.64	<0.05
Negative Behaviour												
All	23	10 318	0.21	0.06	(0.09 to 0.33)	3.4	<0.01	73.14	<0.01	70	3.22	<0.01
Active controls	8	999	0.13	0.1	(−0.06 to 0.32)	1.31	0.19	13.86	0.054	49	0.23	0.83
Passive controls	17	9462	0.23	0.08	(0.08 to 0.37)	3.02	<0.01	59.39	<0.01	73	2.95	<0.01
Selective intervention	12	1185	0.1	0.06	(−0.02 to 0.22)	1.69	0.09	6.94	0.8	0	1.74	0.11
Universal intervention	11	9133	0.31	0.1	(0.10 to 0.51)	2.99	<0.01	65.17	<0.01	85	2.86	<0.05
Social Behaviour												
All	18	10 051	0.13	0.07	(−0.01 to 0.26)	1.84	0.07	70.41	<0.01	76	2.3	<0.05
Active controls	8	1257	−0.03	0.13	(−0.27 to 0.22)	−0.21	0.83	25.17	<0.01	72	1.22	0.27
Passive controls	11	8915	0.21	0.09	(0.04 to 0.39)	2.34	<0.05	44.05	<0.01	77	4.72	<0.01
Selective intervention	7	570	−0.11	0.18	(−0.47 to 0.24)	−0.63	0.53	19.24	<0.01	69	1.49	0.2
Universal intervention	11	9481	0.2	0.08	(0.04 to 0.36)	2.43	<0.05	50.6	<0.01	80	5.28	<0.01
Well-being												
All	10	11 889	0.03	0.05	(−0.06 to 0.13)	0.71	0.48	21.63	<0.05	58	1.12	0.3
Active controls	7	3171	0.11	0.07	(−0.04 to 0.25)	1.43	0.15	9.87	0.13	39	0.78	0.47
Passive controls	6	10 094	−0.1	0.05	(−0.20 to 0.01)	−1.83	0.07	11.67	<0.05	57	1.13	0.32
Selective intervention	1	71	−0.61	–	–	–	–	–	–	–	–	–
Universal intervention	9	11 818	0.05	0.04	(−0.04 to 0.13)	1.05	0.29	16.3	<0.05	51	2.47	<0.05

d, cohen’s d; k, number of studies; MBPs, mindfulness-based programmes; p, significance level; Q, positive false discovery rate; t, Egger’s regression.

#### All RCTs

Across all RCTs, MBP led to small but significant improvements over controls in the categories of mindfulness, attention, executive functioning, negative behaviour, depression and anxiety/stress, but not for well-being or social behaviour (for forest plots see online supplemental G, section 1.1–1.8)

#### RCTs with active control groups

Relative to active controls, MBPs only significantly improved mindfulness and anxiety/stress, with small effects (for forest plots see online supplemental G, section 2.1–2.8).

#### RCTs with passive control groups

Relative to passive controls MBPs significantly improved social behaviour, executive functions, attention, anxiety/stress and negative behaviour. Effect sizes were small (for forest plots see online supplemental G, section 3.1–3.8).

#### Selective MBPs

Selective MBPs relative to controls led to small significant improvements in mindfulness, depression, and anxiety/stress, and moderate significant improvements in attention and executive functions. There was only one selective intervention that used a measure of well-being, so synthesis was not possible (for forest plots see online supplemental G, section 4.1–4.7).

### Universal MBPs

Universal MBPs led to small significant improvements, relative to controls, for social behaviour, executive functioning, attention, and negative behaviour (for forest plots see online supplemental G, section 5.1–5.8).

#### RCTs with follow-ups

Results showed no significant differences between MBPs and controls at follow-up in any outcome category (table 2).

**Table 2:** Analysis of effects of MBPs at follow-up assessment

	k	No of effect sizes	Total n	Intervention effects					Heterogeneity			Publication bias (Egger)	
				Mean effect size (d)	SE	95% CI	z	P value	Q value	P value	I^2^	t	P value
Anxiety/stress	9	21	9467	0.05	0.05	(−0.04 to 0.14)	1.14	0.26	23.57	<0.05	44	1.52	0.16
Attention	5	8	154	0.72	0.48	(−0.22 to 1.66)	1.49	0.14	25.13	<0.01	84	0.06	0.96
Depression	11	16	14 733	0.01	0.03	(−0.04 to 0.07)	0.39	0.70	34.26	<0.01	53	0.65	0.53
Executive functions	9	28	8549	0.28	0.15	(−0.02 to 0.57)	1.81	0.07	63.38	<0.01	84	1.35	0.21
Mindfulness	13	47	9618	−0.01	0.47	(−0.11 to 0.08)	−0.28	0.78	33.67	<0.05	54	1.60	0.13
Negative behaviour	6	11	8396	0.10	0.11	(−0.12 to 0.32)	0.86	0.39	14.72	<0.05	66	0.08	0.44
Social behaviour	7	15	8911	0.11	0.07	(−0.01 to 0.24)	1.77	0.08	34.41	<0.01	68	1.78	0.09
Well-being	8	18	12 014	0.00	0.02	(−0.04 to 0.03)	0.02	0.99	5.72	0.984	0	0.64	0.53

d, cohen’s d; k, number of studies; MBPs, mindfulness-based programmes; n, number of participants; p, significance level; Q, positive false discovery rate; t, Egger’s regression; z, number of standard deviations from the mean.

#### Heterogeneity

Table 1 shows Q values and I^2^ estimates for all analyses. For all RCTs, heterogeneity in all categories was moderate-to-large and significant. For RCTs with active controls, depression, mindfulness and social behaviour showed significant, moderate-to-high heterogeneity. For RCTs with passive controls, all categories showed significant moderate-to-high heterogeneity. For selective interventions, depression, executive functioning, attention and social behaviour showed significant moderate-to-high heterogeneity. For universal interventions, there was significant moderate-to-high heterogeneity for all categories. For studies with follow-ups, all categories with the exception of well-being showed significant moderate-to-high heterogeneity (table 2).

#### Risk-of-bias

**Figure 2:**
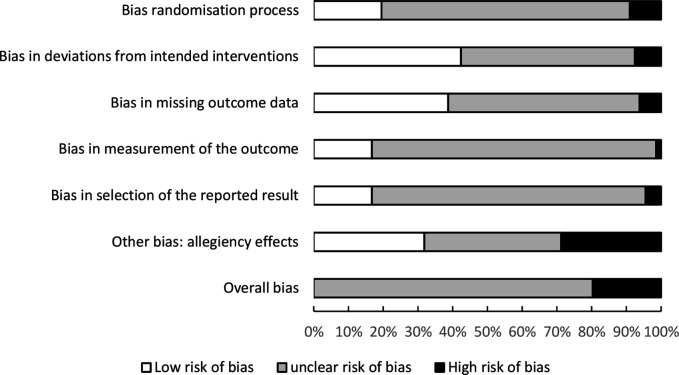
Risk of bias of included studies.

There was low risk-of-bias in 20% of studies for the ‘randomisation process’, 42% for ‘deviations from intended interventions’, 38% for ‘missing outcome data’, 17% for ‘measurement of the outcome’, 17% for ‘bias in selection of the reported result’, 32% for ‘bias caused by allegiance effects’ and 0% for overall bias. A high risk-of-bias existed in 9% of studies for ‘randomisation process’, 8% for ‘deviations from intended interventions’, 6% for ‘missing outcome data, 2% for ‘measurement of the outcome’, 5% for ‘bias in selection of the reported result’, 29% for ‘bias caused by allegiance effects’ and 20% for ‘overall bias’. In all other cases, there was ‘some concern’ of risk-of-bias or was unclear (see figure 2). Full details of the risk-of-bias analysis are in online supplemental E.

#### Publication bias

Table 1 shows Egger’s regression metrics. Begg’s funnel plots for each analysis showing observed and missing treatment effects using the trim-and-fill method^16^ are in online supplemental H. For all studies, there was evidence of publication bias for categories of anxiety/stress, depression, executive functions, mindfulness, negative behaviour and social behaviour. For studies with active controls, there was no evidence of publication bias in any category. For studies with passive controls, there was evidence of publication bias for anxiety/stress, executive functions, mindfulness, negative behaviour and social behaviour. For selective intervention studies, there was no evidence of publication bias in any category. For universal intervention studies, there was evidence of publication bias for anxiety/stress, executive functions, mindfulness, negative behaviour, social behaviour and well-being. For studies with follow-ups, there was no evidence of publication bias in any category. In all significant cases, publication bias was positively skewed suggesting an overestimation of the effects of MBPs in the published literature.

#### Quality of evidence

The quality of the evidence based on the GRADE criteria indicated that, in general, outcomes were subject to potential risks-of-bias, mainly from the inability to double-blind, but also due to inconsistency and to some extent publication bias. Thus, evidence was typically graded as ‘low’ or ‘very low’. The exceptions to were for anxiety/stress for MBPs vs active controls, anxiety/stress for MBPs as selective interventions, and mindfulness skills for MBPs as selective interventions that were graded as of ‘moderate’ quality (see online supplemental F).

#### Moderator analysis

Results of 152 meta-regressions are in online supplemental table S4 for the main analysis and online supplemental table S5 for the follow-up. The following are the 29 significant results, a proportion of which could be false positives resulting from multiple comparisons.

Age was a significant moderator of changes in: anxiety/stress, mindfulness and social behaviour (all universal interventions) and negative behaviour (active controls). Following MBPs, larger benefits were associated with younger age. Age was also a significant moderator of improvements in executive functions and well-being (both vs passive controls), and social behaviour (selective interventions), this time larger SMDs in favour of MBPs were associated with older age.

Dose of MBP significantly moderated changes in negative behaviour (all studies, passive controls, universal interventions), with more MBP leading to larger improvement. Dose of MBP also significantly moderated changes in well-being (passive controls), but with fewer hours equating to better outcomes.

Risk-of-bias moderated changes in mindfulness (all studies, passive controls, universal interventions), attention (active controls), executive functions (active controls), anxiety/stress (all studies, passive controls, universal interventions), depression (passive controls), social behaviour (all, passive controls, universal interventions) and well-being (passive controls, universal outcomes). In all cases a greater risk-of-bias was related to a larger SMD.

With follow-ups, dose of MBP moderated changes in anxiety/stress and negative behaviour, with larger SMDs related to a larger dose; age moderated changes in depression and mindfulness, with larger SMDs related to older age; risk-of-bias moderated changes in attention and mindfulness. For attention a greater risk-of-bias was related to a smaller SMD and for mindfulness a greater risk-of-bias was related to a larger SMD. Length of follow-up did not moderate changes in any outcome category.

## Conclusions and clinical implications

This study, an update of our prior meta-analysis,^1^ presents data from 66 RCTs of MBPs with young people, including a number of recent large-scale studies with substantive follow-up. The number of included studies has doubled since the previous meta-analysis (33 to 66) and the number of participants has increased fivefold (3666–20 168).

Compared with passive control groups, MBPs significantly improved outcomes of attention, executive functioning, social behaviour, negative behaviour and anxiety/stress. However, against active control groups, MBPs only significantly improved anxiety/stress and mindfulness. Universal MBPs were associated with improvements in attention, executive functioning and negative and social behaviour, while selective MBPs improved anxiety/stress, depression, attention, executive functioning and mindfulness. MBPs did not significantly improve well-being relative to controls in any analysis. For all significant improvements following MBP, SMDs were small.

Meta-regressions suggested an inconsistent pattern of results across different control groups and types of intervention. Some analyses suggested that a greater dose of MBP produced better results (for negative behaviour vs passive groups, and as a universal intervention) whereas another suggested a lower dose was more beneficial (well-being vs passive controls). Likewise, some significant results involving the potential moderating effects of age suggested that younger participants benefitted more from MBPs (eg, anxiety/stress and mindfulness for universal interventions), whereas for others, older age predicted better outcomes (eg, executive functions vs passive groups). Importantly, risk-of-bias moderated outcomes in a number of areas, with, studies that had a lower risk-of-bias showing smaller effects.

For the first time, we were able to examine if effects of MBPs were sustained by synthesising results from 23 studies with follow-up assessments. Notably, at follow-up MBPs did not outperform controls for any outcome category, though moderator analyses on follow-up assessments suggested that a larger dose of MBP led to better outcomes for anxiety/stress and negative behaviour, and that older participants benefit more than younger ones in depression and mindfulness outcomes.

Publication bias characterised just under half of all analyses, particularly those involving passive controls or when MBPs were evaluated as universal interventions. Publication bias was always positively skewed suggesting an overestimation of MBPs effects in the published literature. In addition, the quality of the evidence based on the GRADE criteria indicated that, overall, evidence was graded as ‘low’ or ‘very low’.

We can draw on our conceptual model^17^ to help us contextualise and interpret these findings. First, there is growing evidence that the wider context around mindfulness training is both key to mental health and a potential moderator of accessibility, acceptability and effectiveness of socialemotional learning generally and MBPs specifically.^17^ Careful consideration of contextual factors including the socioeconomic status of the area schools serve, the ethnic and cultural make-up of students, and prevailing school climate is needed. Second, the marked heterogenity and moderator effects suggest that MBPs need to consider the population more carefully in terms of age and developmental stage. For example, younger students may only be ready to learn concrete skills, like planning, while older students may be ready to learn the meta-cognitive abilities that only emerge later in adolescence.^18^ Third, MBPs include a broad range of curricula^19–21^, and there may be significant differences in their efficacy.

In terms of future development, a first step is identifying which exact modifiable MBP mechanisms impact mental health. It is also essential that MBPs are accessible and engaging, and codesigning interventions with young people could lead to stepwise improvements. Stratification of MBPs is also likely to be critical. As already noted, mechanisms will likely differ between younger and older adolescents. Moreover, adolescents with different mental health profiles will have different needs. More at-risk individuals may have compromised ability and motivation to engage with MBPs and may need more support.^22^

This updated meta-analysis had several strengths, including the large increase in studies and participants, and the continued focus on RCTs. We also included analysis of follow-up assessments, and exploratory examination of key moderators. There were also limitations. There was no published protocol as this was an update of our previous study and we relied on the original paper to guide the present approach. However, we also updated the analysis plan. It is important to note that the outcome category of ‘well-being’ was not included in our original meta-analysis, nor were the subgroup analyses examining universal and indicated/selective interventions, and we did not previously examine follow-up periods. It would also have improved this review to include an outcome of harm, such as drop-outs or adverse events but insufficient information was available in trial reports. Methodologically, many RCTs did not report data on the key implementation factors (eg, quality, fidelity, mindfulness practice) discussed above, precluding a quantitative analysis. High levels of heterogeneity, particularly for MBPs against passive control conditions and for univeral MBPs suggest the studies in these categories cannot easily be compared. We carried out a large number of exploratory moderator analyses to assess heterogeneity in more detail, however, these were exploratory and statistically uncorrected. The gold standard for a mental health prevention would be to reduce later incidence. However, this requires very large samples and extensive follow-up periods spanning many years. Current symptom levels (eg, of depression) and other relevant mental health variables are, therefore, typically used as proxy variables for incidence in the prevention studies included here. Finally, even though we synthesised 66 RCTs, only a few are well-designed adequately powered studies that also have low risk-of-bias, and in general, the quality of the evidence frames our results as provisional.

In summary, MBPs show promise in terms of improving mental ill health and executive skills but the pattern of results is complex; for example, benefits for depression are limited to selective interventions. There was no evidence that MBPs improved well-being. Notably, there was no indication that any benefits are sustained at follow-up. Future research should carefully consider the context of schools, and implementation factors, as well as the unique needs and developmental stage of young people. Moving forward it will also be useful to disaggregate MBPs to identify the curricula that are most efficacious.

## Data Availability

All data relevant to the study are included in the article or uploaded as online supplemental information.
