# Supplementary material for: Do mindfulness-based programmes improve the cognitive skills, behaviour and mental health of children and adolescents? An updated meta-analysis of randomised controlled trials
Source: Evid Based Ment Health. 2022 Jul 7;25(3):135–42. doi: 10.1136/ebmental-2022-300464 (PMC9340039; doi:10.1136/ebmental-2022-300464)
Supplement: Supplementary data [file ebmental-2022-300464supp001.pdf]

Do Mindfulness-Based Interventions Improve the Cognitive Skills, Behaviour and Mental Health of Children and Adolescents? An updated Meta-Analysis of Randomised Controlled Trials

Supplemental Materials

Darren L. Dunning, Kate Tudor, Lucy Radley, Nicola Dalrymple, Julia Funk, Maris Vainre, Tamsin Ford, Jesus Montero-Marin, Willem Kuyken, and Tim Dalgleish

Corresponding author: Tim Dalgleish, MRC Cognition and Brain Sciences Unit, University of Cambridge, 15 Chaucer Road, Cambridge, CB2 7EF, UK. Email: tim.dalgleish@mrc-cbu.cam.ac.uk

Contents

Table S1 - Details of all the studies included in the meta-analysis, including authors, type of sample, number of participants, type of control group, mean age of sample, type of mindfulness training, duration of mindfulness training, follow-up period (if applicable) and risk of bias ratings.

Table S2 – A list of all included outcome measures with effect sizes by study, including specific measures used and the category it was included in

Table S3 – A list of all included outcome measures with effect sizes by study at follow-up, including specific measure used and the category it was included in

Table S4 – Table showing summary of moderator analyses of the pre-to post effects of mindfulness-based programs

Table S5 - Table showing a complete list of meta-regressions for moderation analysis for studies at pre- to -follow-up

Supplement A – The full references of all studies included in the meta-analysis

Supplement B – Studies not included in quantitative analysis due to absence of outcome variables of interest

Supplement C - Description of all the measures included in the meta-analysis

Supplement D – Hierarchy of outcome measures

Supplement E - The summary Risk of Bias 2.0 assessment of each study

Supplement F - The Grading of Recommendations, Assessment, Development and Evaluation (GRADE) framework

Supplement G – Forest plots for all main analyses

1. **All MBPs**
    - 1.1 *Forest plot showing standardised difference in mean and 95% confidence intervals for* ***anxiety/stress*** *outcomes following a MBP*
    - 1.2 *Forest plot showing standardised difference in mean and 95% confidence intervals for* ***attention*** *outcomes following a MBP*
    - 1.3 *Forest plot showing standardised difference in mean and 95% confidence intervals for* ***depression*** *outcomes following a MBP*
    - 1.4 *Forest plot showing standardised difference in mean and 95% confidence intervals for* ***executive functions*** *outcomes following a MBP*
    - 1.5 *Forest plot showing standardised difference in mean and 95% confidence intervals for* ***mindfulness*** *outcomes following a MBP*
    - 1.6 *Forest plot showing standardised difference in mean and 95% confidence intervals for* ***negative behaviour*** *outcomes following a MBP*
    - 1.7 *Forest plot showing standardised difference in mean and 95% confidence intervals for* ***social behaviour*** *outcomes following a MBP*
    - 1.8 *Forest plot showing standardised difference in mean and 95% confidence intervals for* ***wellbeing*** *outcomes following a MBP*

2. **MBP vs active controls**
    - 2.1 *Forest plot showing standardised difference in mean and 95% confidence intervals for* ***anxiety/stress*** *outcomes following mindfulness training compared to active control groups*

2.2 *Forest plot showing standardised difference in mean and 95% confidence intervals for* ***attention*** *outcomes following mindfulness training compared to active control groups*

2.3 *Forest plot showing standardised difference in mean and 95% confidence intervals for* ***depression*** *outcomes following mindfulness training compared to active control groups*

2.4 *Forest plot showing standardised difference in mean and 95% confidence intervals for* ***executive functions*** *outcomes following mindfulness training compared to active control groups*

2.5 *Forest plot showing standardised difference in mean and 95% confidence intervals for* ***mindfulness*** *outcomes following mindfulness training compared to active control groups*

2.6 *Forest plot showing standardised difference in mean and 95% confidence intervals for* ***negative behaviour*** *outcomes following mindfulness training compared to active control groups*

2.7 *Forest plot showing standardised difference in mean and 95% confidence intervals for* ***social behaviour*** *outcomes following mindfulness training compared to active control groups*

2.8 *Forest plot showing standardised difference in mean and 95% confidence intervals for* ***wellbeing*** *outcomes following mindfulness training compared to active control groups*

3. **MBP vs passive controls**

3.1 *Forest plot showing standardised difference in mean and 95% confidence intervals for* ***anxiety/stress*** *outcomes following mindfulness training compared to passive control groups*

3.2 *Forest plot showing standardised difference in mean and 95% confidence intervals for* ***attention*** *outcomes following mindfulness training compared to passive control groups*

3.3 *Forest plot showing standardised difference in mean and 95% confidence intervals for* ***depression*** *outcomes following mindfulness training compared to passive control groups*

3.4 *Forest plot showing standardised difference in mean and 95% confidence intervals for* ***executive functions*** *outcomes following mindfulness training compared to passive control groups*

3.5 *Forest plot showing standardised difference in mean and 95% confidence intervals for* ***mindfulness*** *outcomes following mindfulness training compared to passive control groups*

3.6 *Forest plot showing standardised difference in mean and 95% confidence intervals for* ***negative behaviour*** *outcomes following mindfulness training compared to passive control groups*

3.7 *Forest plot showing standardised difference in mean and 95% confidence intervals for* ***social behaviour*** *outcomes following mindfulness training compared to passive control groups*

3.8 *Forest plot showing standardised difference in mean and 95% confidence intervals for* ***wellbeing*** *outcomes following mindfulness training compared to active passive groups*

4. **MBPs as Selective interventions**

4.1 *Forest plot showing standardised difference in mean and 95% confidence intervals for* ***anxiety/stress*** *outcomes following mindfulness training as a selective intervention*

4.2 *Forest plot showing standardised difference in mean and 95% confidence intervals for* ***attention*** *outcomes following mindfulness training as a selective intervention*

4.3 *Forest plot showing standardised difference in mean and 95% confidence intervals for* ***depression*** *outcomes following mindfulness training as a selective intervention*

4.4 *Forest plot showing standardised difference in mean and 95% confidence intervals for* ***executive functions*** *outcomes following mindfulness training as a selective intervention*

4.5 *Forest plot showing standardised difference in mean and 95% confidence intervals for* ***mindfulness*** *outcomes following mindfulness training as a selective intervention*

4.6 *Forest plot showing standardised difference in mean and 95% confidence intervals for* ***negative behaviour*** *outcomes following mindfulness training as a selective intervention*

4.7 *Forest plot showing standardised difference in mean and 95% confidence intervals for* ***social behaviour*** *outcomes following mindfulness training as a selective intervention*

5. **MWP as Universal interventions**

5.1 *Forest plot showing standardised difference in mean and 95% confidence intervals for* ***anxiety/stress*** *outcomes following mindfulness training as a universal intervention*

5.2 *Forest plot showing standardised difference in mean and 95% confidence intervals for **attention** outcomes following mindfulness training as a universal intervention*

5.3 *Forest plot showing standardised difference in mean and 95% confidence intervals for **depression** outcomes following mindfulness training as a universal intervention*

5.4 *Forest plot showing standardised difference in mean and 95% confidence intervals for **executive functions** outcomes following mindfulness training as a universal intervention*

5.5 *Forest plot showing standardised difference in mean and 95% confidence intervals for **mindfulness** outcomes following mindfulness training as a universal intervention*

5.6 *Forest plot showing standardised difference in mean and 95% confidence intervals for **negative behaviour** outcomes following mindfulness training as a universal intervention*

5.7 *Forest plot showing standardised difference in mean and 95% confidence intervals for **social behaviour** outcomes following mindfulness training as a universal intervention*

5.8 *Forest plot showing standardised difference in mean and 95% confidence intervals for **wellbeing** outcomes following mindfulness training as a universal intervention*

Supplement H - Funnel plots of publication bias for all main analyses

1. **All MBPs**
   1.1 *Funnel plot for **anxiety/stress** outcomes following Mindfulness training*

   1.2 *Funnel plot for **attention** outcomes following Mindfulness training*

   1.3 *Funnel plot for **depression** outcomes following Mindfulness training*

   1.4 *Funnel plot for **executive function** outcomes following Mindfulness training*

   1.5 *Funnel plot for **mindfulness** outcomes following Mindfulness training*

   1.6 *Funnel plot for **negative behaviour** outcomes following Mindfulness training*

   1.7 *Funnel plot for **social behaviour** outcomes following Mindfulness training*

   1.8 *Funnel plot for **wellbeing** outcomes following Mindfulness training*

2 **MBP vs active controls**
   2.1 *Funnel plot for **anxiety/stress** outcomes following Mindfulness training compared to active control conditions*

2.2 *Funnel plot for* ***attention*** *outcomes following Mindfulness training compared to active control conditions*

2.3 *Funnel plot for* ***depression*** *outcomes following Mindfulness training compared to active control conditions*

2.4 *Funnel plot for* ***executive function*** *outcomes following Mindfulness training compared to active control conditions*

2.5 *Funnel plot for* ***mindfulness*** *outcomes following Mindfulness training compared to active control conditions*

2.6 *Funnel plot for* ***negative behaviour*** *outcomes following Mindfulness training compared to active control conditions*

2.7 *Funnel plot for* ***social behaviour*** *outcomes following Mindfulness training compared to active control conditions*

2.8 *Funnel plot for* ***wellbeing*** *outcomes following Mindfulness training compared to active control conditions*

3 **MBP vs passive controls**

3.1 *Funnel plot for* ***anxiety/stress*** *outcomes following Mindfulness training compared to passive control conditions*

3.2 *Funnel plot for* ***attention*** *outcomes following Mindfulness training compared to passive control conditions*

3.3 *Funnel plot for* ***depression*** *outcomes following Mindfulness training compared to passive control conditions*

3.4 *Funnel plot for* ***executive function*** *outcomes following Mindfulness training compared to passive control conditions*

3.5 *Funnel plot for* ***mindfulness*** *outcomes following Mindfulness training compared to passive control conditions*

3.6 *Funnel plot for* ***negative behaviour*** *outcomes following Mindfulness training compared to passive control conditions*

3.7 *Funnel plot for* ***social behaviour*** *outcomes following Mindfulness training compared to passive control conditions*

3.8 *Funnel plot for* ***wellbeing*** *outcomes following Mindfulness training compared to passive control conditions*

4. **MBPs as Selective interventions**

4.1 *Funnel plot for* ***anxiety/stress*** *outcomes following Mindfulness training as a selective intervention*

4.2 *Funnel plot for* ***attention*** *outcomes following Mindfulness training as a selective intervention*

4.3 *Funnel plot for* ***depression*** *outcomes following Mindfulness training as a selective intervention*

4.4 *Funnel plot for* ***executive function*** *outcomes following Mindfulness training as a selective intervention*

4.5 *Funnel plot for* ***mindfulness*** *outcomes following Mindfulness training as a selective intervention*

4.6 *Funnel plot for* ***negative behaviour*** *outcomes following Mindfulness training as a selective intervention*

4.7 *Funnel plot for* ***social behaviour*** *outcomes following Mindfulness training as a selective intervention*

5. **MBPs as Selective interventions**

5.1 *Funnel plot for* ***anxiety/stress*** *outcomes following Mindfulness training as a universal intervention*

5.2 *Funnel plot for* ***attention*** *outcomes following Mindfulness training as a universal intervention*

5.3 *Funnel plot for* ***depression*** *outcomes following Mindfulness training as a universal intervention*

5.4 *Funnel plot for* ***executive function*** *outcomes following Mindfulness training as a universal intervention*

5.5 *Funnel plot for* ***mindfulness*** *outcomes following Mindfulness training as a universal intervention*

5.6 *Funnel plot for* ***negative behaviour*** *outcomes following Mindfulness training as a universal intervention*

5.7 *Funnel plot for* ***social behaviour*** *outcomes following Mindfulness training as a universal intervention*

5.8 *Funnel plot for* ***wellbeing*** *outcomes following Mindfulness training as a universal intervention*

Supplement I – References for Supplemental materials

Table S1. Details of all the studies included in the quantitative meta-analysis

Authors	Type of RCT/Sample	n Mindfulness	n Controls	Control group	Age (mean)	Mindfulness training	Total duration of training (hours)	Follow up period (months)
Abedini, et al., (2020) [1]	Selective/ Cancer sufferers	19	20	Passive - No contact	12.18	MBCT-C	15	2
Alampay, et al., (2020) [2]	Selective/ At risk	74	90	Active - Handicrafts	11.95	Kamalayan Curriculum	10	N/A
Atkinson & Wade (2015) [3]	Universal/ Typical (all female)	121	83	Passive – no contact	15.7	Adapted from MBCT for depression	2.17	1 & 6
			96	Active - dissonance-based training				
Barnes et al., (2016) [4]	Universal/ Typical	18	22	Active - Health Education	16.2	Mindfulness-based Eating Awareness	18	N/A
Biegal et al., (2009) [5]	Selective/ A mix of mental health disorders	39	46	Passive – no contact	15.7	MBSR	16	6
Bluth et al., (2015) [6]	Selective/ Low academic performance	14	13	Active- Substance abuse control	16.8	Learning to Breathe	6	N/A
Britton et al., (2014) [7]	Universal/ Typical	52	48	Active - Asian history course	11.8	Integrative Comtemplative Pedagogy	6	N/A
Chadli et al., (2016)[8]	Selective/ Females - chronic pain	10	9	Passive - wait-list	16.1	MBSR/MBCT	12	N/A
Cohen et al., (2021)[9]	Selective/ Adolescents with early life stress	21	16	Passive - No contact	14.3	MBSR for teens	2	N/A

Dunning D, *et al. Evid Based Ment Health* 2022; 25:135–142. doi: 10.1136/ebmental-2022-300464

Table S1 (continued). Details of all the studies included in the quantitative meta-analysis

Authors	Type of RCT/Sample	n Mindfulness	n Controls	Control group	Age (mean)	Mindfulness training	Total duration of training (hours)	Follow up period (months)
Cresentini et al., (2016)[10]	Universal/ Typical	16	15	Active - Emotional awareness	7.3	Mindfulness oriented meditation	Not stated	N/A
De Voy (2018) study 1 [11]	Universal/ Typical	20	14	Active - Progressive Muscle Relaxation	14.5	dot.be	6	3
			15	Active - Study Skills				
De Voy (2018) study 2 [11]	Universal/ Typical	41	58	Active - Progressive Muscle Relaxation	14.5	dot.be	6	3
			36	Active- Study Skills				
De Voy (2018) study 3 [11]	Universal/ Typical	6	5	Active - Progressive Muscle Relaxation	14.33	dot.be	6	3
			6	Active - Study Skills				
Delgado-Suárez et al., (2021) [12]	Universal/ Typical	81	83	Active - Relaxation	14	Unlearning	6	4
Desmond & Hanich (2010) [13]	Selective/ Minority, low income	15	25	Passive – no contact	11.5	Mindful Awareness Practice	5.83	N/A
Devcich et al., (2017) [14]	Universal/ Typical	45	46	Active - Emotional literacy	10.24	Pause, Breathe, Smile	8	N/A
Díaz-González, et al. (2018) [15]	Selective/ Mental disorders	41	39	Passive - No contact	14.61	MBSR	12	N/A
Dunning et al., (2022) [16]	Universal/ Typical	234	226	Active - SSS	13.84	dot.be	8	3 & 22-40

Table S1 (continued). Details of all the studies included in the quantitative meta-analysis

**Authors**	**Type of RCT/Sample**	**n Mindfulness**	**n Controls**	**Control group**	**Age (mean)**	**Mindfulness training**	**Total duration of training (hours)**	**Follow up period (months)**
Flook et al. (2010) [17]	Universal/ Typical	32	32	Active - Silent reading	8.2	Mindful Awareness Practice	8	N/A
Flook et al. (2015) [18]	Universal/ Typical	29	37	Passive – wait list	4.7	Mindfulness Kindness curriculum	10	N/A
Franco et al. (2010) [19]	Universal/ Typical	31	30	Passive - wait list	16.8	Meditacion Fluir	15	N/A
Franco Justo (2009) [20]	Universal/ Typical	30	30	Passive – no contact	17.3	Meditacion Fluir	15	N/A
Frank, et al., (2021) [21]	Universal/ Typical	120	110	Passive - No contact	16	Learning to Breathe	Not stated	N/A
Fung, et al., (2019) [22]	Selective/ Ethnic minority, elevated mood symptoms	70	49	Passive - wait-list	14	Learning to Breathe	10	N/A
Ghiroldi, et al., (2020) [23]	Universal/ Typical	232	168	Passive - wait list	8.5	Gaia	12	N/A
Gregoski et al. (2011) [24]	Universal/ Typical (all classified as African American or Black)	53	69<br>44	Active - Life Skills<br>Active - Health Education	15	Breathing Awareness Meditation	14	N/A
Himelstein et al. (2015) [25]	Selective/ Incarcerated males	14	13	Passive – no contact	16.5	Mindfulness-based substance abuse treatment	3	N/A
Ho, et al., (2021) [26]	Selective/ Autistic Spectrum Disorder	19	18	Passive - wait list	13	MYmind	13.5	N/A
Johnson et al. (2016) [27]	Universal/ Typical	115	154	Passive – no contact	13.6	Dot be	6	3

Table S1 (continued). Details of all the studies included in the quantitative meta-analysis

Authors	Type of RCT/Sample	n Mindfulness	n Controls	Control group	Age (mean)	Mindfulness training	Total duration of training (hours)	Follow up period (months)
Johnson et al. (2017) [28]	Universal/ Typical	169	151	Passive – no contact	13.4	Dot be	6	6
		179				Dot be w/parental involvement		
Kiani, et al., (2017) [29]	Selective/ Females - ADHD symptoms	15	15	Passive - waitlist	13.17	The Mindfulness Prescription for Adult ADHD	12	N/A
Koncz et al., (2021) [30]	Universal/ Typical	29	27	Passive - No contact	6.75	Story-based mindfulness	2.4	N/A
Kuyken, et al. (2022) [31]	Universal/ Typical	3768	4144	Passive - No contact	R = 11-16	dot.be	8	12
Lam & Seiden (2019) [32]	Universal/ Typical	53	62	Passive - No contact	12.4	Learning to Breathe	7	N/A
Lassander, et al., (2020) [33]	Universal/ Typical	58	59	Active - relaxation	13.5	dot.be	6.75	3
Lassander, et al., (2021) [34]	Universal/ Typical	1203	1170	Active - Relaxation	R = 12-15	dot.be	6.75	3
			353	Passive - No contact				
Lawler & Esposito (2019) [35]	Selective/ Internationally adopted	33	32	Passive - No contact	7.6	Mindfulness	12	N/A
			31	Active - Executive function training				
Leonard et al. (2013) [36]	Selective/ Incarcerated males	147	117	Active - Cognitive-perception intervention	17.4	Power Source (elements of CBT)	12.5	N/A
Liehr & Diaz (2010) [37]	Selective/ Minority, low income	9	9	Active - Health Education	9.5	Designed by Mindfulness in Schools	2.5	N/A

Table S1 (continued). Details of all the studies included in the quantitative meta-analysis

Authors	Type of RCT/Sample	n Mindfulness	n Controls	Control group	Age (mean)	Mindfulness training	Total duration of training (hours)	Follow up period (months)
Lo, et al., (2017) [38]	Selective/ ADHD	50	50	Passive - wait-list	6.24	Mindfulness Matters	8	N/A
Long, et al., (2018) [39]	Selective/ African American, learning difficulties, disciplinary problems	22	25	Active - Good Behaviour Game	11.69	Mindfulness Skills Training	40.5	N/A
			24	Passive - No contact				
Lu, et al., (2019) [40]	Selective/ left-behind children	21	28	Passive - wait-list	11.7	adapted MBCT	8	N/A
Malboeuf-Hurtubise, et al., (2019) [41]	Selective/ Learning difficulties	13	10	Active -Social skills Curriculum	9-12	Mission Méditation	7	3
Moreno-Gomez & Cejudo (2019) [42]	Universal/ Typical	48	26	Passive - No contact	5.08	MindKinder	36	6
Napoli et al. (2005) [43]	Universal/ Typical	97	97	Passive – no contact	R = 7-10	Attention Academy Program	9	N/A
Parker et al. (2014) [44]	Universal/ Typical	71	40	Passive – wait list	10.1	Master Mind	5	N/A
Poehlmann-Tynan et al. (2016) [45]	Selective/ Economically disadvantaged	12	12	Active - Dialogic Reading	4.7	The Kindness Curriculum	10	3
Quach et al. (2015) [46]	Universal/ Typical	54	53	Passive – wait list	R = 12-17	MBSR	6	N/A
			65	Active - Hatha yoga				
Rawlett et al., (2019) [47]	Selective/ At risk females	12	11	Active - Psycho-ed	11.58	L2B	7.5	N/A

Table S1 (continued). Details of all the studies included in the quantitative meta-analysis

Authors	Type of RCT/Sample	n Mindfulness	n Controls	Control group	Age (mean)	Mindfulness training	Total duration of training (hours)	Follow up period (months)
Ricarte et al. (2015) [48]	Universal/ Typical	45	45	Passive – wait list	8.9	Mindfulness Emotional Intelligence Training	1.5	N/A
Schonert-Reichl et al. (2015) [49]	Universal/ Typical	48	51	Active - Social responsibility program	10.2	MindUP	9	N/A
Semple et al. (2010) [50]	Universal/ Typical	13	12	Passive – wait list	10.5	MBCT-C	18	N/A
Shirk et al. (2014) [51]	Selective/Depressed	20	23	Passive – no contact	15.3	Mindfulness CBT	Not stated	N/A
Shomaker et al. (2017) [52, 54]	Selective/ Girls at risk of type II diabetes	17	16	Active - Cognitive-behavioural	15	Learning to Breathe	6	12
Shomaker et al. (2019) [53]	Selective/ At-risk for excess weight gain	29	25	Active - Health education	14.2	Learning to Breathe	6	6
Sibinga et al. (2013) [55]	Universal/ Typical (all male)	22	19	Active - Health education	12.5	MBSR	10	N/A
Sibinga et al. (2015) [56]	Universal/ Typical	141	159	Active - Health education	12	MBSR	10	N/A
Siffredi et al (2021) [57]	Selective/ Very preterm young adolescents	29	27	Passive - Wait-list	12.15	Mindfulness-based Intervention adapted for adolescents	12	3
Solar (2018) [58]	Selective/ Special education services	5	5	Passive - Wait-list	16	Described as "guided mindfulness"	7.5	N/A

Dunning D, *et al. Evid Based Ment Health* 2022; 25:135–142. doi: 10.1136/ebmental-2022-300464

Table S1 (continued). Details of all the studies included in the quantitative meta-analysis

Authors	Type of RCT/Sample	n Mindfulness	n Controls	Control group	Age (mean)	Mindfulness training	Total duration of training (hours)	Follow up period (months)
Tan & Martin (2014) [59]	Selective/ A variety of mental health disorders	43	37	Passive – no contact	15.4	Taming the Adolescent Mind	7.5	3
Thomas & Atkinson (2016) [60]	Universal/ Typical	16	14	Passive - wait-list	8.83	Paws .B	6	N/A
Viglas & Perlman (2018) [61]	Universal/ Typical	72	55	Passive - No contact	5.2	Mindful Schools	6	N/A
Vohra et al., (2019) [62]	Selective/ Mental health problems	42	39	Passive - No contact	14	MBSR	16	3
Volanen, et al., (2020) [63]	Universal/ Typical	1177	1124	Active - Relaxation	R = 12-15	dot.be	6.75	6
			324	Passive -No contact				
Wright et al., (2019) [64]	Universal/ Typical	45	44	Active - FRIENDS for Life	10.6	MBCT-C	18	N/A
Zelazo et al., (2018) [65]	Universal/ Typical	72	76	Active - Literacy	4.75	Mindfulness/ reflection	10	1
			68	Passive - No contact				

M, Mean; R Range; MBCT-C = Mindfulness-based Cognitive Therapy - Children; MBSR = Mindfulness-based Stress Reduction; L2B = Learning to Breathe

Note: Full references for all studies included in the quantitative analysis can be found in Supplement A

Table S2. A list of all effect sizes by study

Study	Outcome measures used, Cohen's d & Outcome Categories
Abedini, et al., (2020)	**CBCL (Attention = 3.61 (EF, Att)**); AYSR (Attention = 2.44 (EF, Att))
Alampay, et al., (2020)	SMFQ (Depression = -.26 (D))
Atkinson & Wade (2015)	*Versus Passive control group:* **Socio-cultural Attitudes Towards Appearance Scale (Socio-cultural pressures = .12 (SB) subscales);** Clinical Impairment Assessment (Psychosocial impairment = .21 (SB))<br><br>*Versus Active control group:* **Socio-cultural Attitudes Towards Appearance Scale (Socio-cultural pressures = .00 (SB) subscales);** Clinical Impairment Assessment (Psychosocial impairment = -.06 (SB))
Barnes et al. (2016)	Behaviour Assessment System for Children (Perceived Stress = .13 (Anx); Anger Expression = -.34 (NB))
Biegal et al. (2009)	PSS-10 = .41 (Anx); **STAI (Present = .29 (Anx), Past = .41 (Anx));** SCL-90 Interpersonal scale = .31 (SB), SES = .49 (D), SCL-90 (Hostility = .22(NB));
Bluth et al. (2015)	CAMM = .61 (M), Self compassion scale = -.34 (SB), **Social Connectedness Scale = -.18 (SB)**, PSS-10 = -.52 (Anx), **STAI = -.12 (Anx)**, SMFQ = .97 (D)
Britton et al. (2014)	STAI (Total affect disturbance = .32 (Anx), Positive affect = .34 (Anx)); CAMM = .05 (M); YSR (Externalizing problems = .07 (NB), Attention problems = .12 (EF, Att))
Chadli et al., (2016)	Beck Youth Depression & Anxiety Scales (Depression = -.77 (D)**; Anxiety = -.46 (Anx**)), IDPESQ-14 (Psychological distress = -.63 (Anx))
Cohen et al., (2021)	SMFQ = .31 (D); MAAS = -.22 (M)
Cresentini et al., (2016)	CBCL (Withdrawn/depressed = .06 (D), Attention problems = .26 (EF, Att), Rule breaking behaviour = .00 (NB), Aggressive behaviour = .02 (NB)); **CTRS – R (Oppositional = -.09(NB)**, **Cognitive problems = .38 (EF), Hyperactivity = .06 (NB)**, Anxious/shy = .03 (Anx), **ADHD = .31 (NB))** DSM-IV (Inattention = .18 (EF, Att), Hyperactivity = .05 (NB), Restless/impulsive = .22 (NB))
De Voy (2018)1	*Versus Active control group:* WEMWBS = -.29 (W), CAM-R = .17 (M)<br><br>*Versus Attention placebo control group:* WEMWBS =.26 (W); CAM-R = .57(M)

Table S2 continued *A list of all effect sizes by study*

Study	Outcome measures used, Cohen's d & Categories
De Voy (2018)2	*Versus Active control group:* WEMWBS = .30 (W); CAM-R = .06 (M)
	Versus Attention placebo control group: WEMWBS = .49 (W); CAM-R = .02 (M)
De Voy (2018)3	*Versus Active control group:* WEMWBS = .28 (W); CAM-R = .87 (M); STAI = -.14 (Anx); Test of Attention=-.19 (EF, Att)
	Versus Attention placebo control group: WEMWBS = .68 (W); CAM-R = -.43 (M); STAI = -.04 (Anx); Test of Attention = -.29 (EF, Att)
Delgado-Suárez et al., (2021)	The Attitudes Toward Social Aggression Scale (Cognition = .05 (NB), Affective = -.06 (NB), Behaviour = .06 (NB)); MAAS = -.19 (M)
Desmond & Hanich (2010)	BRIEF-teacher = .31 (EF)
Devcich et al., (2017)	SCWBS (Wellbeing: Subjective = .34 (W), Wellbeing: Psychlogical = .40 (W), Wellbeing: General = .22 (W)); MAAS-C =.41 (M)
Díaz-González, et al. (2018)	MAAS = .29 (M); PSS = .23 (Anx); **STAI-C (State = .27 (Anx), Trait = .18 (Anx);** CBCL (Depression = .25 (D), Anxiety = .25 (Anx), Hostility = .18 (NB))
Dunning et al., (2022)	CES-D = .07(D); RCADS = .05 (Anx); CAMM = -.06 (M); WEMWBS = .04 (W); **Stroop (congruent correct = -.06 (EF), neutral correct = -.16 (EF), incongruent correct = -.15 (EF))**, Sustained Attention (Commissions negative = .19 (EF, Att), Commissions neutral = -.17 (EF, Att), Omissions negative = -.13 (EF, Att), Omissions neutral = .01 (EF, Att)); Working memory (negative correct = -.05 (EF), neutral correct = -.05 (EF)); SDQ (prosocial behaviour = .00 (SB))
Flook et al. (2010)	BRIEF-teacher = .07 (EF); BRIEF-Parent = .13 (EF)
Flook et al. (2015)	**Teacher-rated social competence = .26 (SB)**; Sharing task = .33 (SB); DCCS = -.13 (EF); **Flanker task = -.08 (EF, Att)**
Franco et al (2010)	STAI = (State anxiety = .44 (Anx); Trait anxiety = -.24 (Anx))
Franco Justo (2009)	Torrance Test of Creative Thinking = 1.77 (EF)

Table S2 continued *A list of all effect sizes by study*

Study	Outcome measures used, Cohen's d & Categories
Frank, et al., (2021)	**CAMM = -.01 (M)**; SCS (self-comparison = .03(M), self-kindness = -.03 (M), self-judgement = -.04 (M), common humanity = .09 (M)); **Patient Health Questionnaire = -.01 (D)**; **GAD-7 = .13 (Anx)**; RRS = -.15 (D); **Stroop (congruent correct RT = .29 (EF), incongruent correct RT = .27 (EF), congruent correct = .12 (EF), incongruent correct = .15 (EF))**; Working Memory (nback hits = (EF) = -.09, nback false alarms = .00 (EF))
Fung, et al., (2019)	Adolescent Stress Questionnaire (Stress of school performance = .13 (Anx), Stress of peer pressure = -.02 (Anx)) YSR (externalizing problems = .04 (NB), attention problems = .11 (EF, Att)); PSS = .59 (Anx), RSQ = .31 (D)
Ghiroldi, et al., (2020)	TRF (withdrawal/depression = .17 (D); Attention problems = .11 (EF, Att), social problems = .16 (SB), Rule-breaking behaviour = .16 (NB), Aggressive behaviour = .13 (NB))
Gregoski et al. (2011)	*Versus Active control condition:* PSS = .16 (Anx)<br><br>*Versus Active control condition:* PSS = .00 (Anx)
Ho, et al., (2021)	CBCL (attention problems = .10 (EF, Att), externalizing problems = .07 (NB)); BRIEF = -.15 (EF); Social responsiveness scale = -.11 (SB)
Himelstein et al. (2015)	MAAS = .22 (M); Decision-Making Skills =.48 (EF); SES = .81 (D), Behavioual regulation =.80 (NB)
Johnson et al. (2016)	DASS-21 (Depression -.11 (D), Anxiety = -.07 (Anx)); WEMWBS =.01 (W); MAAS = -.19 (M)
Johnson et al. (2017)	*For MBI:* DASS-21 (Depression = -.07 (D), Anxiety = -.23 (Anx)); WEMWBS = .02 (W); CHIME-A (Awareness of Internal Experience = -.07 (M), Awareness of External Experience = -.09 (M), Acting with Awareness = -.30 (M), Acceptance and non-judgement = .14 (M), Decentering and non-reactivity = .17 (M), Openess = .02 (M), Relativity = -.05 (M), Insight = .08 (M)<br><br>*For MBI with parental involvement:* DASS-21 (Depression = -.18 (D), Anxiety = -.20 (A)); EDE-Q (Weight and shape concerns = .13); WEMWBS = -.13 (W); CHIME-A (Awareness of Internal Experience = -.19 (M), Awareness of External Experience = -.09 (M), Acting with Awareness = -.37 (M), Acceptance and non-judgement = .06 (M), Decentering and non-reactivity = .02 (M), Openess = .04 (M), Relativity = -.22 (M), Insight = -.07 (M)
Kiani et al., (2017)	Sustained attention (CPT commissions = .23 (EF, Att), CPT omissions = -.08 (EF, Att), CPT accuracy = .04 (EF, Att)); WISC-IV (digit span forward = -.27 (EF), digit span backward = .26 (EF), letter-number sequencing = .19 (EF)); **Stroop = .56 (EF)**; Tower of London test = .17 (EF)

Table S2 continued *A list of all effect sizes by study*

Study	Outcome measures used, Cohen's d & Categories
Koncz et al., (2021)	*Boys*: Corsi forward = -.24 (EF); Corsi backwards = -.19 (EF); **Go-No/go (omission = -.07 (EF, Att), commission = -.26 (EF, Att)**; Hears and Flowers task = -.26 (EF)
	Girls: Corsi forward = .22 (EF); Corsi backwards = -.71 (EF); **Go-No/go (omission =-1.25 (EF, Att), commission = .57 (EF, Att)**; Hears and Flowers task = .48 (EF)
Kuyken, et al. (in prep)	CES-D = -.01 (D); WEMWBS = .04 (W); SDQ (conduct problems = -.02 (NB), hyperactivity = -.04 (NB), prosocial = .01 (SB)); BRIEF = -.01 (EF); RCADS (social phobia = .28 (Anx), panic disorder = -.06 (Anx), seperation anxiety = -.05 (Anx), generalised anxiety = -.06 (Anx), obsessive-compulsive = -.06 (Anx)); CAMM = -.11 (M); S**DQ teacher report (Conduct = -.04 (NB), hyperactivity = .04(NB))**
Lam & Seiden (2019)	AYSR (attention problems = .57 (EF, Att)); Anxiety = -.02 (Anx); RSS = .35 (D); **BRIEF (global = .54 (EF))**
Lassander, et al., (2020)	**WISC-IV (Rote memory = .21 (EF); Working Memory = .27 (EF))**; NEPSY-II (Response Inhibition = -.27 (EF)), DKEFS Trail making (Cognitive Processing = -.16 (EF) Cognitive Flexibility = .02 (EF))
Lassander, et al., (2021)	*Versus Active control condition:* KINDL-R (physical wellbeing = .06 (W), emotional wellbeing = .14 (W), self-esteem = .02 (D))
	Versus Passive control condition: KINDL-R(physical wellbeing = -.49 (W), emotional wellbeing = -.05 (W), self-esteem = .03 (D))
Lawler & Esposito (2019)	*Versus Passive control condition:* **Flanker task = .18 (EF, Att)**; Star delay task = .23 (EF); Go/No-Go task = .18 (EF, Att)
	Versus Active control condition: **Flanker task =-.31 (EF, Att)**; Star delay task = .18 (EF); Go/No-Go task = .05 (EF, Att)
Leonard et al. (2013)	Attention Network Task = .26 (EF, Att)
Liehr & Diaz (2010)	SMFQ = .81 (D); STAI = .63 (Anx)
Lo, et al., (2017)	SWAN (Inattention = .57 (EF, Att)); CBCL (Hyperactivity = .53 (NB)), Anxiety = .12 (Anx), withdrawn/depressed =.29 (D), attention .45 (EF, Att), aggression = .32 (NB))
Long, et al., (2018)	*Versus Active control group:* Student Externalizing Behavior Screener = -.07 (NB); Student Subjective Wellbeing Questionnaire = -.70 (W)
	Versus Passive control group: Student Externalizing Behavior Screener = .01 (NB); Student Subjective Wellbeing Questionnaire= -.70 (W)

Dunning D, *et al. Evid Based Ment Health* 2022; 25:135–142. doi: 10.1136/ebmental-2022-300464

Table S2 continued *A list of effect sizes by study*

Study	Outcome measures used, Cohen's d & Categories
Lu, et al., (2019)	MAAS = .78(M); Social Anxiety Scale for Children = .65 (Anx); SES = -.01 (D); Positive and Negative Suicide Ideation = .58 (D)
Malboeuf-Hurtubise, et al., (2019)	Behavior Assessment Scale for Children (anxiety = -.07 (Anx); depression = -.74 (D))
Moreno-Gomez & Cejudo (2019)	The Behavioral Assessment System for Children (externalizing problems = .46 (NB), anxiety = .67 (Anx), depression = -.45 (D), Attention deficits = .46 (EF, Att), social skills = .25 (SB))
Napoli et al. (2005)	ADD-H Comprehensive Teacher Rating Scale (Attention = .49 (EF); Social skills = .47 (SB)); Test Anxiety Scale = .39 (Anx); **TEA-Ch (Selective attention subtest = .60 (EF, Att))**
Parker et al. (2014)	**Flanker task =.42 (EF, Att)**; CBCL (Social problems = .41 (NB), Agression problems = .54 (NB), Attention problems = .16 (EF, Att), Anxiety problems = .23 (Anx))
Poehlmann-Tynan et al (2016)	Heads-Toes-Knees-Shoulders = .05 (EF); **Go/No-Go = .10 (EF, Att)**; Observed Empathic Responding (Simulated distress = .47 (SB)); Attachment Story Completion Task (Representations of empathy = .75 (SB) Representations of compassion = -3.26 (SB))
Quach et al (2015)	*Versus Passive control group:* AOSPAN = .33 (EF); PSS = .32 (Anx); **SCARED = -.01 (Anx)**<br>*Versus Active control group:* AOSPAN = .66 (EF); PSS = .40 (Anx); **SCARED = .02 (Anx)**
Rawlett et al., (2019)	RSQ = .27 (Anx); MAAS = .29 (M)
Ricarte et al. (2015)	STAI = .40 (Anx); Trail Making Task = .15 (EF); Perception of Differences Test-Faces = -.16 (EF); **WISC (Digit span = .65 (EF))**
Schonert-Reichl et al. (2015)	**Flanker task = -.14 (EF, Att);** Hearts and Flowers task = -.19 (EF); Interpersonal Reactivity Index (Empathy = -.66 (SB), Perspective Taking =-.61 (SB)); SPQ (Depression = .58 (D)), MAAS-C = .81 (M); **Social Goals Scale (Social responsibility = .19 (SB)); Peer Nominations of Prosociality (Shares = .71 (SB), Trustworthy = 1.03 (SB), Helpful = 1.21 (SB), Takes others' views = 1.43 (SB), Kind = .53 (SB)**, Breaks rules = .85 (NB), Starts fights = 1.04 (NB)); Peer Nominations of Peer Acceptance ('is liked' item only = .62 (SB))
Semple et al. (2010)	**STAI = -.15 (Anx)**; MASC - .65 (Anx); CBCL = .13 (NB)
Shirk et al. (2014)	BDI = -.37 (D)

Table S2 continued *A list of effect sizes by study*

Study	Outcome measures used, Cohen's d & Categories
Shomaker et al. (2017)	MAAS = .34 (M); CES-D = .84 (D); **STAI = .20 (Anx);** PSS = -.14 (Anx)
Shomaker et al. (2019)	PSS = .45 (Anx); BRIEF (parent rating (Inhibit = .30 (EF), shift = .36 (EF), emotion control = .57 (EF), initiate = .32 (EF), working memory = .14 (EF), plan/organise = .56 (EF), materials = .35 (EF), monitor = .56 (EF)), **Flanker = .03 (EF, Att),** List sort = -.17 (EF)
Sibinga et al. (2013)	SCL-90 (Hostility = -.38 (NB); Paranoid ideation = -.30 (Anx), depression = -.33 (D)); Mindfulness (Observe = .14 (M), Accept without judgement = .20 (M), Act with awareness = -.18 (M)); **MASC = .79 (Anx)**, PSS = .05 (Anx); Rumination = .64 (D); DES (Anger = -.66 (NB), Fear = .35 (Anx), Hostility =.08 (NB) Sadness = .06 (D), Shyness = .03 (SB)); Anger Expression (Angry temperament = .38 (NB), Reactivity = -.03 (NB)); Conflict = -.40 (NB)
Sibinga et al. (2015)	PSS = .24 (Anx), CAMM = .52 (M), **The Children's Depression Inventory = .31 (D)**, SCL-90 (Hostility = .26 (NB)); **MASC = .08 (Anx);** DES (Sadness = .19 (D), Anger = .07(NB), Contempt = .31 (NB), Fear = .29 (Anx), Self-hostility = .35 (NB), Shyness = .20 (SB)); **Agression Scale = .14 (NB**); CRSQ (Rumination = .24 (D), Distraction = .13 (EF, Att), Problem solving = -.21 (EF)); Children's Post-Traumatic Symptom Severity Checklist (Post-Traumatic Symptoms = .35 (Anx); Reexperiencing = .31 (Anx), Depression = .31 (D))
Siffredi et al (2021)	BRIEF = .86 (EF); WISC-IV (Letter-number sequencing = .03 (EF)); **Flanker (processing speed =.79 (EF), inhibition = .63 (EF));** Social goal scale = .12 (SB)
Solar (2018)	CAMM = .27 (M); PSS = .28 (Anx); **The Revised Children's Manifest Anxiety Scale = -.84 (Anx)**
Tan & Martin (2014)	SES = .31 (D); Avoidance and Fusion Questionnaire for Children (Psychological Inflexibility = .43 (EF)); CAMM = .69 (M); CBCL = .00 (NB)
Thomas & Atkinson (2016)	Teacher-reported Attention Checklist = .85 (EF, Att); **NEPSY-II (inhibition = .34 (EF)**
Viglas & Perlman (2018)	SDQ (prosocial = .43 (SB), hyperactivity = .39 (NB), conduct problems = .24 (NB), peer problems = .03 (NB))
Vohra et al., (2019)	PSS = .12 (Anx); CAMM = -.09 (M); The Behavior Assessment System for Children-2 (Parent Rating Scales (externalizing problems = .07 (NB), behavioural symptoms index = .02 (NB)), Teacher Rating Scales (Parent Rating Scales (externalizing problems = .15 (NB), behavioural symptoms index = .23 (NB))
Volanen, et al., (2020)	*Versus active controls:* BDI = .03 (D)
	Versus passive controls: BDI = .00 (D)

Table S2 continued *A list of effect sizes by study*

Study	Outcome measures used, Cohen's d & Categories
Wright et al., (2019)	RCADS = (anxiety =.20 (Anx), Depression = .03 (D)); SDQ (prosocial behaviour- child = -.09 (SB), **prosocial behaviour- teacher = -.12 (SB), prosocial behaviour- parent = -.13 (SB)**); CAMM = .31 (M) The CNS Vital Signs (attention control = .05 (EF, Att), shifting attention = -.48 (EF, Att), sustained attention = - .02(EF, Att))
Zelazo et al., (2018)	*Versus active controls:* Heads-Toes-Knees-Shoulders = .14 (EF); Peg tapping = .32 (EF); **Minnesota executive function scale= -.02 (EF)**
	Versus passive controls: Heads-Toes-Knees-Shoulders = .05 (EF); Peg tapping = .22 (EF); **Minnesota executive function scale= -.14 (EF)**

Note. Anx, Anxiety; Att, Attention; D, Depression; EF, Executive Functions; M, Mindfulness; NB, Negative Behaviour; SB, Social Behaviour; W, Wellbeing; AOSPAN, Automated Operation Span task; BDI, Beck Depression Inventory; BRIEF, Behaviour Rating Inventory of Executive Function; CAM-R, Cognitive and Affective Mindfulness Scale, CAMM, Child and Adolescent Mindfulness Measure; CBCL, Child Behaviour Checklist; CES-D, Center for Epidemiologic Studies Depression Scale; CHIME-A, Comprehensive Inventory of Mindfulness Experiences; CTRS, The Conners Teacher Rating Scale; CRSQ, Children's Response Styles Questionnaire; DASS-21, Depression Anxiety Stress Scale; CDI, Children's Depression Inventory; DCCS, Dimensional Change Card Sort; GAD-7, General Anxiety Disorder-7; MAAS, Mindful Attention Awareness Scale; MASC, Multidimensional Anxiety Scale for Children; NEPSY, A Developmental NEuroPSYchological Assessment, PSS, Perceived Stress Scale; RCADS, Revised Child Anxiety and Depression Scale; RRS, Runmination Response Scale; RSQ, Relationship Scales Questionnaire; SCARED, Screen for Child Anxiety Related Disorders; SCS, Self-Compassion Scale; SCL-90, Symptoms Checklist-90; SCWBS, Stirling Children's Wellbeing Scale, SDQ, Strengths and Difficulties Questionnaire, SES, Rosenberg's Self-Esteem Scale; SMFQ, The Short Mood and Feelings Questionnaire, SPQ, Seattle Personality Questionnaire; STAI, State Trait Anxiety Inventory; TEA-Ch, test of Everyday Attention for Children; TRF, Teacher Report Form; WEMWBS, Warwick-Edinburgh Mental Wellbeing Scale.

Outcomes in bold were chosen for the meta-analysis due to position on outcome hierarchy

Table S3. A list of all included outcome measures, with effect sizes, by study, at follow-up

Study	Outcome measures used, Cohen's d & Categories
Abedini, et al., (2020)	CBCL Attention = 3.09 (EF, Att); **YSR Attention = 2.64 (EF, Att)**
Atkinson & Wade (2015)	*One month follow up:* (versus Passive control group**) Socio-cultural Attitudes Towards Appearance Scale (Socio-cultural pressures = .00 (SB) subscales);** Clinical Impairment Assessment (Psycholsocal impairment = -.08 (SB)); (versus Active control group) **Socio-cultural Attitudes Towards Appearance Scale (Socio-cultural pressures = .02 (SB) subscales)**; Clinical Impairment Assessment (Psychosocial impairment = .32 (SB))<br>*Six month follow up:* (versus Passive control group) **Socio-cultural Attitudes Towards Appearance Scale (Socio-cultural pressures = .39 (SB) subscales)**; Clinical Impairment Assessment (Psycholsocal impairment = .23 (SB)); (versus Active control group) **Socio-cultural Attitudes Towards Appearance Scale (Socio-cultural pressures = .01 (SB) subscales)**; Clinical Impairment Assessment (Psychosocial impairment = -.29 (SB))
Biegal et al. (2009)	PSS-10 = .63 (Anx); **STAI (Present = .69 (Anx), Past = .57 (Anx))**; SCL-90 (Interpersonal scale = .53 (SB)), Depression = .58 (D), Anxiety = .43 (Anx), Hostility = .42 (NB)); SES = .70 (D)
De Voy (2018)1	(Versus Active control group) WEMWBS = .51 (W); CAM-R = .19 (M); (Versus Attention placebo control group) WEMWBS = -.16 (W); CAM-R = .21 (M)
De Voy (2018)2	(Versus Active control group) WEMWBS = -.04 (W); CAM-R = -.08 (M); (Versus Attention placebo control group) WEMWBS = .13 (W); CAM-R = .06 (M)
De Voy (2018)3	(Versus Active control group) WEMWBS = -.11 (W); CAM-R = -.02 (M); STAI = -.67 (Anx); Attention =-.34 (EF, Att); (Versus Attention placebo control group) WEMWBS = .45 (W); CAM-R = .32 (M); STAI = -.04 (Anx); Attention = .01 (EF, Att)
Delgado-Suárez et al., (2021)	The Attitudes Toward Social Aggression Scale (Cognition = .28 (NB), Affective = -.51 (NB), Behaviour = .20 (NB)); MAAS = .08 (M)
Dunning et al., (2022)	*Three month follow-up:* CES-D = .01 (D); RCADS = .08 (Anx); CAMM = -.13 (M); WEMWBS = -.02 (W); SDQ (prosocial behaviour = -.05 (SB))<br>*Long-term follow-up (22-40 months):* CES-D = .003(D); RCADS = .09 (Anx); CAMM = -.06 (M); WEMWBS = -.05 (W); SDQ (prosocial behaviour = .02 (SB))
Johnson et al. (2016)	DASS-21 (depression = -.33 (D)); GAD-7 = .20 (Anx); WEMWBS = -.07 (W); MAAS = -.32 (M)

Table S3 (continued). A list of all included outcome measures, with effect sizes, by study at follow-up

Study	Outcome measures used, Cohen's d & Categories
Johnson et al. (2017)	*Six month follow-up: (For MBI)* DASS-21 (Depression = -.04 (D), Anxiety = -.33 (Anx); WEMWBS = -.01 (W); CHIME-A (Awareness of Internal Experience = -.23 (M), Awareness of External Experience = -.08 (M), Acting with Awareness = -.21 (M), Acceptance and non-judgement = -.03 (M), Decentering and non-reactivity = -.22 (M), Openness = .23 (M), Relativity = -.28 (M), Insight = .09 (M); (For MBI with parental involvement) DASS-21 (Depression = -.05 (D), Anxiety = -.00 (A)); WEMWBS = -.05 (W); CHIME-A (Awareness of Internal Experience = .04 (M), Awareness of External Experience = .00 (M), Acting with Awareness = -.11 (M), Acceptance and non-judgement = .15 (M), Decentering and non-reactivity = .05 (M), Openness = .18 (M), Relativity = -.06 (M), Insight = .18 (M)<br>*Twelve month follow-up: (For MBI)* DASS-21 (Depression = -.01 (D), Anxiety = .03 (Anx); WEMWBS = .10 (W); CHIME-A (Awareness of Internal Experience = -.15 (M), Awareness of External Experience = -.08 (M), Acting with Awareness = -.13 (M), Acceptance and non-judgement = .06 (M), Decentering and non-reactivity = .03 (M), Openness = .10 (M), Relativity = -.17 (M), Insight = .11 (M); (For MBI with parental involvement) DASS-21 (Depression = -.121 (D), Anxiety = .099 (A)); WEMWBS = -.059 (W); CHIME-A (Awareness of Internal Experience = .13 (M), Awareness of External Experience = .06 (M), Acting with Awareness = -.01 (M), Acceptance and non-judgement = .16 (M), Decentering and non-reactivity = .19 (M), Openness = .03 (M), Relativity = -.02 (M), Insight = .16 (M)
Kuyken, et al. (in prep)	CES-D = -.01 (D); WEMWBS = -.01 (W); SDQ (conduct problems = .03 (NB), hyperactivity = -.003 (NB), prosocial = -.04 (SB)); BRIEF = -.001 (EF); RCADS (social phobia = -.02 (Anx), panic disorder = -.01 (Anx), separation anxiety = .01 (Anx), generalised anxiety = -.01 (Anx), obsessive-compulsive = -.03 (Anx)); CAMM = -.11 (M**); SDQ teacher report (Conduct = -.09 (NB), hyperactivity = .08 (NB))**
Lassander, et al., (2020)	**WISC (Rote memory = .02 (EF), Working Memory = -.002 (EF))**; Response Inhibition = -.38 (EF) Cognitive Processing = .07 (EF) Cognitive Flexibility = .02 (EF)
Lassander, et al., (2021)	Versus Active control condition: KINDL-R (physical wellbeing = .04 (W), emotional wellbeing = .06 (W), self-esteem = .003 (D)); Versus Passive control condition: KINDL-R (physical wellbeing = - .39 (W), emotional wellbeing = -.01 (W), self-esteem = .02 (D));
Malboeuf-Hurtubise, et al., (2019)	Behaviour Assessment Scale for Children (anxiety = -.37 (Anx); depression = -.98 (D))
Moreno-Gomez & Cejudo (2019)	The Behavioural Assessment System for Children (externalizing problems = .41 (NB, anxiety = .61 (Anx), depression = -.30 (D), Attention deficits = .43 (EF, Att), social skills = .41 (SB))
Poehlmann-Tynan et al (2016)	Heads-Toes-Knees-Shoulders = .60 (EF); **Go/No-Go = .65 (EF, Att**); Empathic Responding (Simulated distress = -.32 (SB)); Attachment Stem Completion Task (Representations of empathy = 1.79 (SB); Representations of compassion = -1.33 (SB))
Shomaker et al. (2017)	MAAS = .17 (M); CES-D = .68 (D)

Shomaker et al. (2019)	PSS = -.47 (Anx); BRIEF (parent rating (Inhibit = .35 (EF), shift = .15 (EF), emotion control = .55 (EF), initiate = .46 (EF), working memory = .10 (EF), plan/organise = .45 (EF), materials = .11 (EF), monitor = .62 (EF)), **Flanker = .16 (EF),** List sort = -.19 (EF)

Table S3 (continued). A list of all included outcome measures, with effect sizes, by study at follow-up

Study	Outcome measures used, Cohen's d & Categories
Siffredi et al (2021)	BRIEF = .86 (EF); Letter-number sequencing = .03 (EF**); Flanker (processing speed =.79 (EF), inhibition = .63 (EF)**); Social goal scale = .12 (SB)
Tan & Martin (2014)	SES = .60 (D); Avoidance and Fusion Questionnaire for Children (Psychological Inflexibility = -.78 (EF)); CAMM = .96 (M); CBCL = -.488 (NB)
Vohra et al., (2019)	PSS = .34 (Anx); CAMM = -.34 (M); The BASC-2 (Parent Rating Scales (externalizing problems = .07 (NB), behavioural symptoms index = .23 (NB))
Volanen, et al., (2020)	*(Versus active controls)* BDI = .06 (D); (Versus passive controls) BDI = .05 (D)
Zelazo et al., (2018)	(Versus active controls) Heads-Toes-Knees-Shoulders = .23 (EF); Peg tapping = .24 (EF); **Minnesota executive function scale= .19 (EF);** (Versus passive controls) Heads-Toes-Knees-Shoulders = .03 (EF); Peg tapping = .46 (EF); **Minnesota executive function scale= -.15 (EF)**

Anx, Anxiety; Att, Attention; D, Depression; EF, Executive Functions; M, Mindfulness; NB, Negative Behaviour; SB, Social Behaviour; W, Wellbeing; BDI, Beck Depression Inventory; BRIEF, Behaviour Rating Inventory of Executive Function; CAM-R, Cognitive and Affective Mindfulness Scale, CAMM, Child and Adolescent Mindfulness Measure; CBCL, Child Behaviour Checklist; CES-D, Center for Epidemiologic Studies Depression Scale; CHIME-A, Comprehensive Inventory of Mindfulness Experiences; DASS-21, Depression Anxiety Stress Scale; GAD-7, General Anxiety Disorder-7; MAAS, Mindful Attention Awareness Scale; PSS, Perceived Stress Scale; RCADS, Revised Child Anxiety and Depression Scale; SCL-90, Symptoms Checklist-90; SDQ, Strengths and Difficulties Questionnaire; SES, Rosenberg's Self-Esteem Scale; STAI, State Trait Anxiety Inventory; WEMWBS, Warwick-Edinburgh Mental Wellbeing Scale.

Outcomes in bold were chosen for the meta-analysis due to position on outcome hierarchy

Table S4 Summary of moderator analyses of the pre- to post-intervention effects of mindfulness-based programmes (MBPs)

	Age			Total hours of MBP			Risk-of-Bias		
	β	*SE*	*p*	β	*SE*	*p*	β	*SE*	*p*
Anxiety/Stress									
All	-0.03	0.02	0.05	0.01	0.01	0.17	-0.04	0.02	<.05
Active controls	-0.02	0.02	0.30	0.01	0.01	0.26	0.01	0.03	0.64
Passive controls	-0.04	0.02	0.08	0.01	0.01	0.13	-0.07	0.03	<.01
Selective intervention	-0.02	0.02	0.30	-0.01	0.02	0.44	0.03	0.03	0.46
Universal intervention	-0.05	0.02	<.01	0.01	0.01	0.08	-0.05	0.02	<.01
Attention									
All	0.00	0.02	0.93	0.01	0.01	0.27	-0.13	0.04	0.75
Active controls	0.02	0.02	0.28	0.05	0.03	0.05	-0.07	0.03	<.05
Passive controls	0.03	0.04	0.44	0.01	0.02	0.57	0.07	0.07	0.33
Selective intervention	-0.02	0.05	0.74	0.10	0.07	0.14	0.07	0.11	0.56
Universal intervention	-0.01	0.03	0.74	0.01	0.01	0.46	-0.04	0.04	0.32
Depression									
All	0.00	0.02	0.93	0.00	0.01	0.76	-0.01	0.02	0.68
Active controls	0.05	0.07	0.45	-0.02	0.02	0.54	0.06	0.04	0.11
Passive controls	0.00	0.02	0.84	0.02	0.02	0.22	-0.05	0.02	<.05
Selective intervention	0.03	0.04	0.42	-0.03	0.03	0.28	0.02	0.05	0.66
Universal intervention	-0.05	0.03	0.06	0.03	0.02	0.14	-0.01	0.03	0.66
Executive Functioning									
All	0.03	0.02	0.12	0.01	0.01	0.30	-0.03	0.03	0.38
Active controls	0.01	0.01	0.34	-0.01	0.02	0.75	-0.05	0.03	<.05
Passive controls	0.06	0.03	<.05	0.01	0.01	0.32	0.00	0.04	0.96
Selective intervention	-0.01	0.04	0.72	0.05	0.04	0.29	0.01	0.07	0.84
Universal intervention	0.04	0.02	0.09	0.01	0.01	0.48	-0.03	0.03	0.33
Mindfulness									
All	-0.06	0.03	0.07	0.01	0.02	0.77	-0.09	0.02	<.01
Active controls	-0.08	0.04	0.07	0.02	0.03	0.59	-0.05	0.56	0.34
Passive controls	-0.02	0.06	0.66	-0.01	0.02	0.76	-0.08	0.03	<.01
Selective intervention	0.01	0.05	0.90	-0.02	0.02	0.29	-0.08	0.04	0.07
Universal intervention	-0.12	0.04	<.01	0.04	0.03	0.17	-0.09	0.04	<.05
Negative Behaviour									
All	0.00	0.02	0.94	0.02	0.01	<.05	-0.03	0.03	0.38
Active controls	-0.09	0.03	<.01	0.03	0.04	0.54	-0.06	0.07	0.36
Passive controls	0.01	0.02	0.62	0.02	0.01	<.05	-0.03	0.03	0.31
Selective intervention	-0.01	0.02	0.57	0.00	0.02	0.97	0.01	0.03	0.79
Universal intervention	0.03	0.03	0.44	0.03	0.01	<.05	-0.06	0.05	0.20

Dunning D, *et al. Evid Based Ment Health* 2022; 25:135–142. doi: 10.1136/ebmental-2022-300464

Table S4 (continued) Summary of moderator analyses of the pre-to post effects of mindfulness-based programs

	Age			Total hours of MBP			Riskof-Bias		
	β	*SE*	*p*	β	*SE*	*p*	β	*SE*	*p*
Social Behaviour									
All	-0.01	0.02	0.72	0.00	0.01	0.79	-0.06	0.03	<.05
Active controls	0.08	0.05	0.10	-0.01	0.05	0.88	0.05	0.13	0.71
Passive controls	-0.01	0.02	0.46	0.00	0.01	0.97	-0.08	0.01	<.01
Selective intervention	0.14	0.05	<.01	0.01	0.05	0.81	0.13	0.20	0.50
Universal intervention	-0.03	0.02	<.05	0.00	0.01	1.00	-0.08	0.02	<.01
Wellbeing									
All	0.04	0.07	0.56	0.00	0.02	0.95	-0.03	0.03	0.32
Active controls	0.04	0.08	0.62	-0.03	0.02	0.12	-0.02	0.04	0.68
Passive controls	0.37	0.17	<.05	-0.03	0.01	<.05	0.05	0.02	0.03
Selective intervention	-	-	-	-	-	-	-	-	-
Universal intervention	-0.02	0.06	0.71	0.00	0.02	0.95	-0.05	0.01	<.01

Note: it was not possible analyse Selective interventions in the Wellbeing category as there was just a single study

Table S5 Summary of moderator analyses of the pre-intervention to follow-up effects of mindfulness-based programs

		Age			Total hours of MBP			Risk-of-Bias			Follow-up periods		
	k	β	*SE*	*p*	β	*SE*	*p*	β	*SE*	*p*	β	*SE*	*p*
Anxiety/Stress	10	-0.04	0.03	0.22	0.03	0.01	<.01	-0.03	0.04	0.45	0.00	0.01	0.73
Attention	5	-0.01	0.13	0.93	0.01	.05	0.91	0.53	0.27	<.05	-0.29	0.39	0.46
Depression	12	0.07	0.03	<.05	0.00	0.01	0.93	0.00	0.03	0.92	0.00	0.01	0.74
Executive Functions	9	-0.03	0.05	0.58	0.03	0.02	0.14	0.13	0.10	0.21	-0.02	0.06	0.69
Mindfulness	13	0.27	0.10	<.01	0.00	0.05	0.99	-0.07	0.03	<.01	0.00	0.02	0.97
Negative Behaviour	6	-0.04	0.05	0.46	0.02	0.01	<.05	0.02	0.06	0.69	-0.01	0.05	0.81
Social Behaviour	7	-0.02	0.03	0.49	0.01	0.01	0.29	-0.03	0.04	0.47	0.01	0.02	0.73
Wellbeing	9	0.06	0.12	0.63	-0.02	0.02	0.44	-0.02	0.02	0.30	0.00	0.00	0.41

Supplement A

References for all the studies included in the quantitative meta-analysis

1 Abedini S, Habibi M, Abedini N, et al. A randomized clinical trial of a modified mindfulness-based cognitive therapy for children hospitalized with cancer. *Mindfulness.* 2021;12(1):141-51.

2 Alampay LP, Galvez Tan LJ, Tuliao AP, et al. A pilot randomized controlled trial of a mindfulness program for Filipino children. *Mindfulness*. 2020;11(2):303-16.

3 Atkinson MJ, Wade TD. Mindfulness-based prevention for eating disorders: A school-based cluster randomized controlled study. *Int J Eat Disord*. 2015;48(7):1024-37.

4 Barnes VA, Kristeller JL. Impact of mindfulness-based eating awareness on diet and exercise habits in adolescents. *Int J Complement Altern Med*. 2016;3(2).

5 Biegel GM, Brown KW, Shapiro SL, et al. Mindfulness-based stress reduction for the treatment of adolescent psychiatric outpatients: A randomized clinical trial. *J Consult Clin Psychol.* 2009;77(5):855.

6 Bluth K, Campo RA, Pruteanu-Malinici S, et al. A school-based mindfulness pilot study for ethnically diverse at-risk adolescents. *Mindfulness*. 2016;7(1):90-104.

7 Britton WB, Lepp NE, Niles HF, et al. A randomized controlled pilot trial of classroom-based mindfulness meditation compared to an active control condition in sixth-grade children. *J Sch Psychol*. 20141;52(3):263-78.

8 Chadi N, McMahon A, Vadnais M, et al. Mindfulness-based intervention for female adolescents with chronic pain: A pilot randomized trial. *J Can Acad Child Adolesc Psychiatry*. 2016;25(3):159.

9 Cohen ZP, Cosgrove KT, Akeman E, Coffey S, Teague K, Hays-Grudo J, Paulus MP, Aupperle RL, Kirlic N. The effect of a mindfulness-based stress intervention on neurobiological and symptom measures in adolescents with early life stress: a randomized feasibility study. BMC complementary medicine and therapies. 2021 Dec;21(1):1-4.

10 Crescentini C, Capurso V, Furlan S, et al. Mindfulness-oriented meditation for primary school children: Effects on attention and psychological well-being. *Front Psychol*. 2016;7:805.

11 De Voy, M. (2019). *The impact of mindfulness training on schoolchildren's academic performance* (Order No. 28357344). Available from ProQuest Dissertations & Theses A&I. (2475221279). Retrieved from https://ezp.lib.cam.ac.uk/login?url=https://www.proquest.com/dissertations-theses/impact-mindfulness-training-on-schoolchildrens/docview/2475221279/se-2?accountid=9851

12 Delgado-Suárez I, López-del-Hoyo Y, García-Campayo J, et al. Testing the Efficacy of 'Unlearning', a Mindfulness and Compassion-Based Programme for Cultivating Nonviolence in Teenagers: A Randomised Controlled Trial. *Front Psychol*. 2021;12.

13 Desmond CT, Hanich L, Millersville PA. The effects of mindful awareness teaching practices on the executive functions of students in an urban, low-income middle school. Millersville University: Pennsylvania Research Report. 2010;1:12.

14 Devcich DA, Rix G, Bernay R, Graham E. Effectiveness of a mindfulness-based program on school children's self-reported well-being: A pilot study comparing effects with an emotional literacy program. *J Appl Sch Psychol*. 2017;33(4):309-30.

15 Díaz González MD, Pérez Dueñas C, Sánchez Raya MA, et al. Mindfulness-based stress reduction in adolescents with mental disorders: A randomised clinical trial. *Psicothema*. 2018;30(2):165-70.

16 Dunning, DL, Ahmed, S, Foulkes L. et al. The impact of mindfulness training in early adolescence on affective executive control, and on later mental health during-COVID-19: A randomised controlled trial (under review).

17 Flook L, Smalley SL, Kitil MJ, et al. Effects of mindful awareness practices on executive functions in elementary school children. J Appl Sch Psychol. 2010;26(1):70-95.

18 Flook L, Goldberg SB, Pinger L, et al. Promoting prosocial behavior and self-regulatory skills in preschool children through a mindfulness-based Kindness Curriculum. *Dev Psychol*. 2015;51(1):44.

19 Franco C, Mañas I, Cangas AJ, et al. The applications of mindfulness with students of secondary school: Results on the academic performance, self-concept and anxiety. In World Summit on Knowledge Society 2010 Sep 22 (pp. 83-97). Springer, Berlin, Heidelberg.

20 Franco Justo C. Effects of a meditation program on verbal creative levels in a group of students in late secondary education. *Suma Psicológica*. 2009;16:113-20.

21 Frank JL, Broderick PC, Oh Y, et al. The effectiveness of a teacher-delivered mindfulness-based curriculum on adolescent social-emotional and executive functioning. *Mindfulness.* 2021;12(5):1234-51.

22 Fung J, Kim JJ, Jin J, et al. A randomized trial evaluating school-based mindfulness intervention for ethnic minority youth: Exploring mediators and moderators of intervention effects. *J Abnorm Child Psychol*. 2019;47(1):1-9.

23 Ghiroldi S, Scafuto F, Montecucco NF, et al. Effectiveness of a school-based mindfulness intervention on children's internalizing and externalizing problems: The Gaia project. *Mindfulness*. 2020;11(11):2589-603.

24 Gregoski MJ, Barnes VA, Tingen MS, et al. Breathing awareness meditation and LifeSkills Training programs influence upon ambulatory blood pressure and sodium excretion among African American adolescents. *J Adolesc Health*. 2011;48(1):59-64.

25 Himelstein S, Saul S, Garcia-Romeu A. Does mindfulness meditation increase effectiveness of substance abuse treatment with incarcerated youth? A pilot randomized controlled trial. *Mindfulness.* 2015;6(6):1472-80.

26 Ho RY, Zhang D, Chan SK, Gao TT, Lee EK, Lo HH, Au Yeung P, Lai KY, Bögels SM, de Bruin EI, Wong SY. Brief Report: Mindfulness training for Chinese adolescents with autism spectrum disorder and their parents in Hong Kong. Journal of Autism and Developmental Disorders. 2021 Nov;51(11):4147-59

27 Johnson C, Burke C, Brinkman S, et al. Effectiveness of a school-based mindfulness program for transdiagnostic prevention in young adolescents. *Behav Res Ther*. 2016;81:1-1.

28 Johnson C, Burke C, Brinkman S, et al. A randomized controlled evaluation of a secondary school mindfulness program for early adolescents: Do we have the recipe right yet? *Behav Res Ther*. 2017;99:37-46.

29 Kiani B, Hadianfard H, Mitchell JT. The impact of mindfulness meditation training on executive functions and emotion dysregulation in an Iranian sample of female adolescents with elevated attention-deficit/hyperactivity disorder symptoms. *Aust J Psychol.* 2017;69(4):273-82.

30 Koncz A, Köteles F, Demetrovics Z, et al. Benefits of a Mindfulness-Based Intervention upon School Entry: A Pilot Study. *Int J Environ Res Public Health.* 2021;18(23):12630.

31 Kuyken W, Ball S, Crane C, et al. Effectiveness and cost-effectiveness of a universal school-based mindfulness training programme compared with normal school provision in reducing risk of mental

health problems and promoting well-being in adolescence: results of the MYRIAD cluster randomised controlled trial. Evid Based Ment Health 2022; doi:10.1136/ebmental-2021-300396 .

32 Lam K, Seiden D. Effects of a brief mindfulness curriculum on self-reported executive functioning and emotion regulation in Hong Kong adolescents. *Mindfulness*. 2020;11(3):627-42.

33 Lassander M, Hintsanen M, Suominen S, et al. The effects of school-based mindfulness intervention on executive functioning in a cluster randomized controlled trial. *Dev Neuropsychol.* 2020;45(7-8):469-84.

34 Lassander M, Hintsanen M, Suominen S, et al. Effects of school-based mindfulness intervention on health-related quality of life: moderating effect of gender, grade, and independent practice in cluster randomized controlled trial. *Qual Life Res*. 2021;30(12):3407-19.

35 Lawler JM, Esposito EA, Doyle CM, et al. A preliminary, randomized-controlled trial of mindfulness and game-based executive function trainings to promote self-regulation in internationally-adopted children. *Dev Psychopathol*. 2019;31(4):1513-25.

36 Leonard NR, Jha AP, Casarjian B, et al. Mindfulness training improves attentional task performance in incarcerated youth: a group randomized controlled intervention trial. *Front Psychol*. 2013;4:792.

37 Liehr P, Diaz N. A pilot study examining the effect of mindfulness on depression and anxiety for minority children. *Arch Psychiatr Nurs*. 2010;24(1):69-71.

38 Lo HH, Wong SW, Wong JY, et al. The effects of family-based mindfulness intervention on ADHD symptomology in young children and their parents: a randomized control trial. *J Atten Disord*. 2020;24(5):667-80.

39 Long AC, Renshaw TL, Camarota D. Classroom management in an urban, alternative school: A comparison of mindfulness and behavioral approaches. *Contemp Sch Psychol*. 2018;22(3):233-48.

40 Lu R, Zhou Y, Wu Q, et al. The effects of mindfulness training on suicide ideation among left-behind children in China: A randomized controlled trial. *Child Care Health Dev*. 2019;45(3):371-9.

41 Malboeuf-Hurtubise C, Taylor G, Mageau GA. Impact of a mindfulness-based intervention on basic psychological need satisfaction and internalized symptoms in elementary school students with severe learning disabilities: Results from a randomized cluster trial. *Front Psychol*. 2019:2715.

42 Moreno-Gómez AJ, Cejudo J. Effectiveness of a mindfulness-based social–emotional learning program on psychosocial adjustment and neuropsychological maturity in kindergarten children. *Mindfulness*. 2019;10(1):111-21.

43 Napoli M, Krech PR, Holley LC. Mindfulness training for elementary school students: The attention academy. J Appl Sch Psychol. 2005;21(1):99-125.

44 Parker AE, Kupersmidt JB, Mathis ET, et al. The impact of mindfulness education on elementary school students: Evaluation of the Master Mind program. *Adv Sch Ment Health Promot.* 2014;7(3):184-204.

45 Poehlmann-Tynan J, Vigna AB, Weymouth LA, et al. A pilot study of contemplative practices with economically disadvantaged preschoolers: Children's empathic and self-regulatory behaviors. *Mindfulness*. 2016;7(1):46-58.

46 Quach D, Mano KE, Alexander K. A randomized controlled trial examining the effect of mindfulness meditation on working memory capacity in adolescents. *J Adolesc Health.* 2016;58(5):489-96.

47 Rawlett KE, Friedmann E, Thomas SA. Mindfulness based intervention with an attentional comparison group in at risk young adolescents: a pilot randomized controlled trial. *Integr Med Res*. 2019;8(2):101-6.

48 Ricarte JJ, Ros L, Latorre JM, et al. Mindfulness-based intervention in a rural primary school: Effects on attention, concentration and mood. *Int J Cogn Ther*. 2015;8(3):258-70.

49 Schonert-Reichl KA, Oberle E, Lawlor MS, et al. Enhancing cognitive and social–emotional development through a simple-to-administer mindfulness-based school program for elementary school children: A randomized controlled trial. *Dev Psychol*. 2015;51(1):52.

50 Semple RJ, Lee J, Rosa D, et al. A randomized trial of mindfulness-based cognitive therapy for children: Promoting mindful attention to enhance social-emotional resiliency in children. *J Child Fam Stud*. 2010;19(2):218-29.

51 Shirk SR, DePrince AP, Crisostomo PS, et al. Cognitive behavioral therapy for depressed adolescents exposed to interpersonal trauma: An initial effectiveness trial. *Psychotherapy*. 2014;51(1):167.

52 Shomaker LB, Berman Z, Burke M, et al. Mindfulness-based group intervention in adolescents at-risk for excess weight gain: A randomized controlled pilot study. Appetite. 2019 Sep 1;140:213-22.

53 Shomaker LB, Bruggink S, Pivarunas B, et al. Pilot randomized controlled trial of a mindfulness-based group intervention in adolescent girls at risk for type 2 diabetes with depressive symptoms. *Complement Ther Med*. 2017;32:66-74.

54 Shomaker LB, Pivarunas B, Annameier SK, et al. One-year follow-up of a randomized controlled trial piloting a mindfulness-based group intervention for adolescent insulin resistance. *Front Psychol.* 2019;10:1040.

55 Sibinga EM, Perry-Parrish C, Chung SE, et al. School-based mindfulness instruction for urban male youth: A small randomized controlled trial. *Prev Med*. 2013;57(6):799-801.

56 Sibinga E, Webb L, Ghazarian SR, et al. School-based mindfulness instruction: An RCT. *Pediatr*. 2016;137(1).

57 Siffredi V, Liverani MC, Hüppi PS, et al. The effect of a mindfulness-based intervention on executive, behavioural and socio-emotional competencies in very preterm young adolescents. *Sci Rep*. 2021;11(1):1-2.

58 Solar E. Mindfulness Meditation with High School Students Who Receive Special Education Services: Research Recommendations from a Pilot Study. *J Am Acad Spec Educ Prof.* 2018;146:161.

59 Tan L, Martin G. Taming the adolescent mind: A randomised controlled trial examining clinical efficacy of an adolescent mindfulness-based group programme. *Child Adolesc Ment Health*. 2015;20(1):49-55.

60 Thomas G, Atkinson C. Measuring the effectiveness of a mindfulness-based intervention for children's attentional functioning. *Educational & Child Psychology*. 2016;33(1):51-64.

61 Viglas M, Perlman M. Effects of a mindfulness-based program on young children's self-regulation, prosocial behavior and hyperactivity. *J Child Fam Stud.* 2018;27(4):1150-61.

62 Vohra S, Punja S, Sibinga E, et al. Mindfulness-based stress reduction for mental health in youth: a cluster randomized controlled trial. *Child Adolesc Ment Health*. 2019;24(1):29-35.

63 Volanen SM, Lassander M, Hankonen N, et al. Healthy learning mind–Effectiveness of a mindfulness program on mental health compared to a relaxation program and teaching as usual in schools: A cluster-randomised controlled trial. *J Affect Disord*. 2020;260:660-9.

64 Wright KM, Roberts R, Proeve MJ. Mindfulness-based cognitive therapy for children (MBCT-C) for prevention of internalizing difficulties: A small randomized controlled trial with Australian primary school children. *Mindfulness*. 2019;10(11):2277-93.

65 Zelazo PD, Forston JL, Masten AS, et al. Mindfulness plus reflection training: Effects on executive function in early childhood. *Front Psychol*. 2018;9:208.

Supplement B

Studies that meet the inclusion criteria, but were excluded from qualitative analysis due to absence of appropriate outcome variables

Study	Outcomes
Barnes et al (2004)[168]	Systolic blood pressure, Diastolic blood pressure, Heart rate
Barnes et al (2008)[169]	Systolic blood pressure, Diastolic blood pressure, Heart rate, Sodium handling
Beattie et al (2019)[170]	Reasons given for non-practice of mindfulness, Home practice of intervention, Intervention lesson attendance
Beattie et al (2020)[171]	Intention to practice, Amount of mindfulness practice
Khoshkerder et al (2019)[172]	Body image concerns, Eating attitudes
Malboeuf-Hurubise et al (2021)[173]	Mental health difficulties, Basic psychological need
Salmoirago-Blotcher et al (2018)[174]	Retention rates, Class attendance, Intervention adherence, Physical activity, Diet
Tong Lee et al (2020)[175]	Body Mass Index, Step count, Happiness, Emotion, Interaction, Disruption, Play
Wright et al (2011)[[176]	Blood pressure, Heart Rate
Yuan (2021)[177]	Resilience, Emotional intelligence

Dunning D, *et al. Evid Based Ment Health* 2022; 25:135–142. doi: 10.1136/ebmental-2022-300464

Supplement C

Description of measures

Note: A list of which measures were used in each study can be found on Tables S2 and S3

Measures of Depression

Beck Depression Inventory-II (BDI-II)

The BDI-II (1) is a frequently used self-report measure assessing levels of depressive symptoms that can be used for ages 13 to 80. The measure has good internal consistency with Cronbach's alpha ranging between .85 and .96 and high test-retest reliability for a re-application range from one week to six months with r ranging between .73 to .96 as well as high convergent, discriminant, construct and criterion validity (2). Psychometric properties of the BDI-II have specifically been investigated in adolescent populations and were shown to be comparable to adult samples (3).

Beck Youth Depression & Anxiety Scales

The Beck Youth Depression & Anxiety Scales are two of the five self-report measures of the Beck Youth Inventory (BYI) (4). The scales can be used for assessing levels of depressive and anxiety symptoms in children and adolescents aged 7-14 and have demonstrated high internal consistency (average Cronbach's alpha .9), satisfactory test-retest reliability as well as good construct and criterion validity (5, 6) in these age groups.

Behaviour Assessment System for Children, Second Edition (BASC-2)

The BASC-2 (7) is an instrument for the evaluation of behaviour and self-perception of children, adolescents and young adults aged 2-25 that assesses a variety of problem behaviours, internalizing problems such as depression and anxiety, school problems, relations with peers and, adaptive skills. It consists of a self-report rating, a parent and a teacher rating scale. Internal consistency and test-retest reliability were found to be good with Cronbach's alpha ranging between .8 and .9 and r ranging between .7 and .9 (7).

Centre for Epidemiologic Studies Depression Scale-Revised (CESD-R)

The CESD-R (8) is a self-report measure designed for assessing depression. The measure has good psychometric properties (Cronbach's alpha >.9 and high convergent, discriminant and construct validity (9)). It is mostly used in psychiatric epidemiology studies (8).

Child Behaviour Checklist (CBCL)

The CBCL (9) is a parent-rating scale to assess various aspects of psychopathology and social competencies in childhood and adolescence (age groups 4-18). Internal consistency for the anxiously depressed subscale and test-retest reliability for the whole instrument were found to be good to acceptable (10, 11). The CBCL demonstrated good criterion validity as defined by the ability to distinguish between individuals with and without psychopathology (12).

Dunning D, *et al. Evid Based Ment Health* 2022; 25:135–142. doi: 10.1136/ebmental-2022-300464

The Child PTSD Symptom Scale (CPSS)

The CPSS (13) is a self-report questionnaire designed to assess the severity of posttraumatic stress symptoms in children and adolescents aged 8 to 18 who have been exposed to trauma. The measure was shown to have good psychometric properties (13).

Children's Response Styles Questionnaire (CRSQ)

The CRSQ is a self-report questionnaire measuring children's responses to their own depressive symptoms (14). The questionnaire assesses to what extend respondents react to depressed mood with rumination, distraction and problem-solving. The questionnaire has good psychometric properties and there are high correlations between the rumination subscale and depressive symptoms (15).

Depression Anxiety Stress Scale (DASS) - Depression

The depression sub-scale of the self-report instrument DASS (16) assesses levels of depressive and anxiety symptoms as well as stress levels. Whereas the DASS was found to have good psychometric properties in validation studies in adult samples (17), evidence is mixed as to whether the DASS has sufficient construct validity and can differentiate between depression, anxiety and distress in children and adolescents (18, 19)

Differential Emotions Scale (DES)

The DES (20) is self-report measures assessing 12 discrete negative and positive emotions. Psychometric properties of the DES were found to be good, however, younger age was found to be associated with lower internal consistency of the questionnaire (21).

Fragebogen zur Erfassung der gesundheitsbezogenen Lebensqualität von Kindern und Jugendlichen (KINDL)

The KINDL (22) is a German language self-report questionnaire measuring quality of life, specifically in the domains physical well-being, emotional well-being, self-esteem, family, friends, and everyday functioning in children and adolescents. Psychometric properties were found to be satisfactory to good (23).

Patient Health Questionnaire-9 (PHQ-9)

The PHQ-9 (24) is a widely used self-report measure of depressive symptoms and a screening instrument to make tentative depression diagnoses. The questionnaire was found to have high internal consistency with Cronbach's alpha ranging between .86 and .89, high test-re-test reliability as well as good construct and criterion validity (24). The PHQ-9 was also found to be a suitable instrument for assessing depressive symptoms and screening for depressive episodes in adolescents (25, 26).

Positive and Negative Suicide Ideation (PANSI)

The PANSI (27) is a self-report measure for assessing the frequency of positive and negative thoughts related to suicidal behaviour. In samples of adolescents from different countries, the PANSI was shown two have good internal consistency with Cronbach's alpha ranging between .83 and .94 and high construct validity (27, 28).

Response Styles Questionnaire (RSQ)

The RSQ (29) is a self-repot questionnaire measuring the respondents tendency to react with rumination, distraction and problem-solving when feeling distressed. The questionnaire, especially the rumination subscale, is used frequently in research and demonstrated good psychometric properties (30).

Revised Child Anxiety and Depression Scale (RCADS)

The RCADS (31) is a self-report measure which is widely used to screen for symptoms of depression and anxiety disorder. The questionnaire and the single depression and anxiety sub-scales demonstrated high internal consistency with an average Cronbach's alpha of .94 for the whole scale and average Cronbach's alphas of .74–.85 for the sub-scales as well as good construct validity (32).

Rosenberg's Self-Esteem Scale (RSES)

The RSES (33) is a widely used self-report measure for self-esteem in adolescents and adults. The RSES was shown to be a reliable (Cronbach's alpha between .88 and .90) and valid measure of self-esteem (34).

Seattle Personality Questionnaire (SPQ)

The SPQ (35) is a self-report measure for children that assesses the dimensions anxiety, conduct problems, somatization, depression, school dislike and additionally includes a lie scale. There is little evidence on the psychometric properties of the questionnaire.

Symptoms Checklist-90-R (SCL-90-R)

The SCL-90-R (36) is a frequently used self-report measure for individuals aged 13 years and older that covers a broad range of mental health problems. Symptom dimensions of the measure are somatization, obsessive-compulsive, interpersonal sensitivity, depression, anxiety, hostility, phobic anxiety, paranoid ideation and psychoticism. Additionally, the scale has three global distress indices. Reliability, construct and criterion validity of the SCL-90-R were found to be high (37).

Teacher Engagement Report Form (TERF)

The TERF (38) is a measure in which teachers rate their students on aspects of affective, behavioural, and cognitive engagement. The TERF was found to have good internal consistency, external validity as measured by agreement of the TERF scores with student self-rating was overall acceptable but correlations were only moderate (39).

The Children's Depression Inventory (CDI)

The CDI (40) is a self-report measure that assess levels of depressive symptoms in children and adolescents aged 7-17 and is a modification of the Beck Depression Inventory . The CDI was found a reliable and valid measure that can differentiate between children with and without depression diagnoses sufficiently accurately (41).

The Short Mood and Feelings Questionnaire (SMFQ)

The SMFQ (42) is a self-report questionnaire designed to measure depressive symptomatology in children and adolescents aged 6-17 years. Internal consistency of the SMFQ (Cronbach's alpha = 0.85) was found to be high and convergent and construct validity was found to be acceptable to good (42, 43).

Measures of Anxiety/ Stress

Adolescent Stress Questionnaire (ASQ)

The ASQ (49) is a self-report instrument assessing subjective stressor load in different domains of adolescent stressor exposure such as school performance, romantic relationships and peer pressure. Internal consistency and test-retest reliability were found to be moderate to good and evidence regarding criterion and construct validity is inconclusive (49, 50).

Beck Youth Depression & Anxiety Scales

The Beck Youth Depression & Anxiety Scales are two of the five self-report measures of the Beck Youth Inventory (BYI) (4). The scales can be used for assessing levels of depressive and anxiety symptoms in children and adolescents aged 7-14 and have demonstrated high internal consistency (average Cronbach's alpha .9), satisfactory test-retest reliability as well as good construct and criterion validity (5, 6) in these age groups.

Behaviour Assessment System for Children, Second Edition (BASC-2)

The BASC-2 (7) is an instrument for the evaluation of behaviour and self-perception of children, adolescents and young adults aged 2-25 that assesses a variety of problem behaviours, internalizing problems such as depression and anxiety, school problems, relations with peers and, adaptive skills. It consists of a self-report rating, a parent and a teacher rating scale. Internal consistency and test-retest reliability were found to be good with Cronbach's alpha ranging between .8 and .9 and r ranging between .7 and .9 (7).

Child Behaviour Checklist (CBCL)

The CBCL (9) is a parent-rating scale to assess various aspects of psychopathology and social competencies in childhood and adolescence (age groups 4-18). Internal consistency for the anxiously depressed subscale and test-retest reliability for the whole instrument were found to be good to acceptable (10, 11). The CBCL demonstrated good criterion validity as defined by the ability to distinguish between individuals with and without psychopathology (12).

The Child PTSD Symptom Scale (CPSS)

The CPSS (13) is a self-report questionnaire designed to assess the severity of posttraumatic stress symptoms in children and adolescents aged 8 to 18 who have been exposed to trauma. The measure was shown to have good psychometric properties (13).

Depression Anxiety Stress Scale (DASS)

The depression sub-scale of the self-report instrument DASS (16) assesses levels of depressive and anxiety symptoms as well as stress levels. Whereas the DASS was found to have good psychometric properties in validation studies in adult samples (17), evidence is mixed as to whether the DASS has sufficient construct validity and can differentiate between depression, anxiety and distress in children and adolescents (18, 19).

Differential Emotions Scale (DES)

The DES (20) is self-report measures assessing 12 discrete negative and positive emotions. Psychometric properties of the DES were found to be good, however, younger age was found to be associated with lower internal consistency of the questionnaire (21).

General Anxiety Disorder-7 Questionnaire (GAD-7)

The GAD-7 (51) is a self-report questionnaire that is commonly used to assess levels of generalized anxiety symptoms as well as to screen for generalized anxiety disorder. The GAD-7 was found to have good psychometric properties and validity as a screener for generalized anxiety disorder (51, 52). The questionnaire was also shown to be a valid and reliable measure in adolescent populations (53).

Multidimensional Anxiety Scale for Children (MASC)

The MASC (54) is a self-report scale that measures a wide spectrum of common anxiety symptoms in children and adolescents. The questionnaire and its single sub-scales were found to have moderate to high internal consistency, good test-retest reliability as well as validity in measuring anxiety (55, 56).

Perceived Stress Scale (PSS)

The PSS (57) is a commonly used self-report questionnaire that assesses the degree to which individuals appraise situations in their lives as stressful. Psychometric properties of the questionnaire have been studied intensively and were found to be overall found acceptable (58). The PSS is also occasionally used to assess perceived stress in adolescents (59), however, the psychometric properties of the questionnaire in the population are less well-known.

Relationship Scales Questionnaire (RSQ)

The RSQ (60) is a self-report questionnaire on which respondents rate themselves in response to a series of statements about their close relationship. It also contains items on anxiety related to relationships/ being alone. Findings on the psychometric properties and factor structure of the RSQ are inconsistent (61).

Revised Child Anxiety and Depression Scale (RCADS)

The RCADS (31) is a self-report measure which is widely used to screen for symptoms of depression and anxiety disorder. The questionnaire and the single depression and anxiety sub-scales demonstrated high internal consistency with an average Cronbach's alpha of .94 for the whole scale and average Cronbach's alphas of .74–.85 for the sub-scales as well as good construct validity (32).

Screen for Child Anxiety Related Emotional Disorders (SCARED)

The SCARED (62) is a questionnaire that is designed to screen for anxiety disorders in children and adolescents. It consists of a self-report questionnaire on which children and adolescents rate themselves and a scale on which parents answer questions about their children. The SCARED demonstrated good internal consistency with Cronbach's alpha α ranging between .74 and.93 as well as good test-retest reliability, discriminative validity (both between anxiety and other disorders and within anxiety disorders) and moderate child-parent agreement (62).

Social Anxiety Scale for Children – Revised (SASC-R)

The SASC-R (63) is a self-report measure for children's experience of social anxiety. The measure was found to have acceptable internal consistency and high convergent validity (63).

State Trait Anxiety Inventory for Children (STAIC)

The STAIC (63) is a frequently used self-report measure that assesses state and trait anxiety in children. Both the trait and the state anxiety scale were found to have good internal consistency (64)

and the measure demonstrated acceptable criterion validity as it was shown to discriminate between children with anxiety disorders and children without mental health disorders (65).

Symptoms Checklist-90-R (SCL-90-R)

The SCL-90-R (36) is a frequently used self-report measure for individuals aged 13 years and older that covers a broad range of mental health problems. Symptom dimensions of the measure are somatization, obsessive-compulsive, interpersonal sensitivity, depression, anxiety, hostility, phobic anxiety, paranoid ideation and psychoticism. Additionally, the scale has three global distress indices. Reliability, construct and criterion validity of the SCL-90-R were found to be high (37).

Test Anxiety Scale (TAS)

The TAS (66) is a self-report measure of test anxiety in children. The scale was shown to have adequate internal consistency and test-retest reliability as well as good convergent and discriminant validity (67).

The Conners Teacher Rating Scale (CTRS)

The CTRS (68) is a questionnaire on which teachers can assess children's behaviour in the classroom. The measure covers a range of facets of children’s behaviour such as hyperactivity-impulsivity, perfectionism, cognitive problems, social problems, oppositionality, and anxiousness/shyness. Test-retest reliability and internal consistency of the CTRS were found to be satisfactory and criterion validity of the measure as indexed by the ability of the measure to discriminate between children with and without attention deficit hyperactivity disorder was found to be good (68).

The Revised Children's Manifest Anxiety Scale (RCMAS)

The RCMAS (69) is a self-report instrument designed to assess anxiety in children and adolescents. The measure was shown to have good test-retest reliability (70) as well as high concurrent validity (71).

Indices de détresse psychologique – Enquête Santé Québec (IDPESQ-14)

The IDPESQ-14 Psychological distress scale (72) is a self-report measurement scale used to assess negative emotions leading to depression and anxiety in teens.

Dunning D, *et al. Evid Based Ment Health* 2022; 25:135–142. doi: 10.1136/ebmental-2022-300464

Measures of Well-Being

Warwick-Edinburgh Mental Wellbeing Scale (WEMWBS)

The WEMWBS (73) is a commonly used self-report measure of well-being, which covers different aspects of positive mental health. The WEMWBS was shown to have strong psychometric properties in adult (73) as well as adolescent samples (74).

Stirling Children's Wellbeing Scale (SCWBS)

The SCWBS (75) is a self-report measure of well-being that has been developed to assess different aspects of positive mental health in children aged 8 to 15 years. The SCWBS was shown to have good internal consistency, construct validity and external reliability (75).

Fragebogen zur Erfassung der gesundheitsbezogenen Lebensqualität von Kindern und Jugendlichen (KINDL)

The KINDL (22) is a German language self-report questionnaire measuring quality of life, specifically in the domains physical well-being, emotional well-being, self-esteem, family, friends, and everyday functioning in children and adolescents. Psychometric properties were found to be satisfactory to good (23).

Student Subjective Wellbeing Questionnaire (SSWQ)

The SSWQ (76) is a self-report measure for assessing youths' subjective wellbeing at school. The SSWQ demonstrated good internal consistency and high structural and external validity (76).

Dunning D, *et al. Evid Based Ment Health* 2022; 25:135–142. doi: 10.1136/ebmental-2022-300464

Measures of Mindfulness

Children Adolescents Mindfulness Measure (CAMM)

The CAMM (77) is a self-report questionnaire that has been developed and is frequently used to measure mindfulness in children and adolescents over the age of 9 years. The CAMM was shown to have good internal consistency as well as good construct and convergent validity (78).

Cognitive and Affective Mindfulness Scale (CAMS)

The CAMS (79) is a self-report questionnaire for assessing mindfulness in adults. The measure was shown to have acceptable internal consistency and high convergent validity in adult samples (80), however, its psychometric properties in adolescent samples have not yet been established.

Mindful Attention Awareness Scale (MAAS)

The MAAS (81) is a frequently used self-report measure of mindfulness which measures the construct based on the definition of mindfulness as attention to and awareness of what is occurring in the present moment. The MAAS has been adapted to assess mindfulness in adolescents (MAAS-A) and demonstrated good psychometric properties in adolescent samples (82).

Self-Compassion Scale (SCS)

The SCS (83) is a self-report measure which assesses thoughts, emotions, and behaviours associated with self-compassion. While the SCS was originally developed to measure self-compassion in adults, the measure was also shown to be a reliable and valid measure of self-compassion in adolescents (84).

Comprehensive Inventory of Mindfulness Experiences - Adolescents (CHIME-A)

The CHIME-A (85) is a self-report questionnaire that measures multiple facets of mindfulness such as awareness of internal experiences, awareness of external experiences, acting with awareness, accepting and non-judgmental orientation, decentring and nonreactivity in adolescents. Factor analyses supported the construct validity of the measure, however, the overall internal consistency of the CHIME-A was found to be poor (85).

Measures of Social Behaviour

Interpersonal Reactivity Index (IRI)

The IRI (86) is a self-report measure of empathy, which assesses both affective and cognitive components of the construct. While the measure has originally been developed for adults, its psychometric properties have also been investigated in adolescent samples (87). The IRI appears to be an adequate measure for assessing empathy in adolescents (87).

ADD-H Comprehensive Teacher Rating Scale (ACTERS) – social skills

The ACTERS (88) is a scale on which teachers can rate their students regarding to attention and hyperactive behaviour. Sub-scales of the measure are attention, hyperactivity, social skills and oppositional behaviour. The ACTERS was demonstrated to have high internal consistency and validity as a measure of attention deficit hyperactivity disorder (89).

Attachment Story Completion Task (ASCT)

The ASCT (90) is a behavioural task designed to assess the extent to which children view themselves as participating in a secure relationship with a parent who is available and responsive to their signals and needs in different circumstances. The task has been used to elucidate children's family experiences rather than to measure their social skills (90).

Clinical Impairment Assessment (CIA)

The CIA (91) is a self-report measure designed to assess psychosocial impairment due to eating disorder features. The measure was shown to have satisfactory psychometric properties in samples of young adults (92).

Differential Emotions Scale (DES)

The DES (20) is self-report measures assessing 12 discrete negative and positive emotions. Psychometric properties of the DES were found to be good, however, younger age was found to be associated with lower internal consistency of the questionnaire (21).

Emotion Awareness (Shyness) QUESTIONNAIRE NOT DESCRIBED

Empathic Responding (own measure)

The authors of the study (93) let observers rate the degree to which children behaved empathic during a standardized distress task (94). Interrater reliability was computed using intraclass correlations (ICC) and found to be good, ICC = 0.72–0.99 (93).

Sharing task (own test)

The sharing task has specifically been designed by the authors of (95) for the purpose of their study. It consisted of consisted of four separate trials in which children distributed stickers between themselves and a target recipient. The task has not been investigated outside the context of the study.

Social Goal Scale (SGS)

The SGS (96) is a self-report scale on which adolescents can rate themselves concerning their social responsibility goals, their social relationship goals and their social status goals. Psychometric properties of the measure have not been investigated intensively, however, there is evidence that

the internal consistency of the sub-scales of the measure is good with Cronbach's alpha ranging between .70 and .82 (96).

Socio-Cultural Attitudes Towards Appearance Scale (SATAQ)

The SATAQ (97) is a self-report measure of societal influences on body image and eating disturbances in adolescents and young adults. The SATAQ was shown to be a reliable and valid measure of multiple aspects of a societal influence (97).

Strength and Difficulties Questionnaire (SDQ) - prosocial behaviour

The SDQ (98) is a questionnaire which in a self-report and a parent-report version covers domains of children's and adolescents' psychopathology (i. e. emotional symptoms conduct problems, hyperactivity-inattention, and peer problems) as well as personal strengths (i. e. prosocial behaviour). The prosocial behaviour subscale was found to have acceptable internal consistency with Cronbach's alpha ranging between .62 and .68 as well as good convergent and divergent validity (99).

Symptoms Checklist-90-R (SCL-90-R)

The SCL-90-R (36) is a frequently used self-report measure for individuals aged 13 years and older that covers a broad range of mental health problems. Symptom dimensions of the measure are somatization, obsessive-compulsive, interpersonal sensitivity, depression, anxiety, hostility, phobic anxiety, paranoid ideation and psychoticism. Additionally, the scale has three global distress indices. Reliability, construct and criterion validity of the SCL-90-R were found to be high (37).

Teacher Engagement Report Form (TERF)

The TERF (38) is a measure in which teachers rate their students on aspects of affective, behavioural, and cognitive engagement. The TERF was found to have good internal consistency, external validity as measured by agreement of the TERF scores with student self-rating was overall acceptable but correlations were only moderate (39).

Teacher-Rated Social Competence Scale (TSC)

The TSC (100) is a questionnaire on which teacher can rate the social competencies of their students. The overall scale and its two subscales prosocial behaviour and emotion regulation were shown to have good internal consistency (95), however, the external validity of the measure is still unknown.

Behaviour Assessment System for Children, Second Edition (BASC-2)

The BASC-2 (7) is an instrument for the evaluation of behaviour and self-perception of children, adolescents and young adults aged 2-25 that assesses a variety of problem behaviours, internalizing problems such as depression and anxiety, school problems, relations with peers and, adaptive skills. It consists of a self-report rating, a parent and a teacher rating scale. Internal consistency and test-retest reliability were found to be good with Cronbach's alpha ranging between .8 and .9 and r ranging between .7 and .9 (7).

The Social Connectedness Scale - Revised

The Social Connectedness Scale – Revised (101) is a self-report questionnaire for assessing to which degree youth feel connected to others in their social environment. The measured demonstrated high internal consistency as well as good convergent, discriminant and construct validity (101).

Measures of Negative Behaviour

Aggression Scale (AS)

The AS (102) is a self-Report Measure of aggressive behaviour in young adolescents. The scale was found to have high internal consistency with Cronbach's alpha ranging from .85 through .92 and to correlate highly with teachers' ratings of aggressive behaviour (102).

Behaviour regulation (3rd person observations)

The rating system for behavioural regulation was designed by the study's authors (103). Ratings were made by the staff of the detention centre in which the study took place. In the rating system, points were given for good behaviour during school time, recreation, and during structured activities. Points were taken away for negative behaviour such as not following staff directives, verbal and physical aggression.

Child Behaviour Checklist (CBCL)

The CBCL (9) is a parent-rating scale to assess various aspects of psychopathology and social competencies in childhood and adolescence (age groups 4-18). Internal consistency for the anxiously depressed and the aggressive behaviour subscale and test-retest reliability for the whole instrument were found to be good to acceptable (10, 11). The CBCL demonstrated good criterion validity as defined by the ability to distinguish between individuals with and without psychopathology (12).

Differential Emotions Scale (DES)

The DES (20) is self-report measures assessing 12 discrete negative and positive emotions. Psychometric properties of the DES were found to be good, however, younger age was found to be associated with lower internal consistency of the questionnaire (21).

The Conners Teacher Rating Scale (CTRS)

The CTRS (68) is a questionnaire on which teachers can assess children's behaviour in the classroom. The measure covers a range of facets of children's behaviour such as hyperactivity-impulsivity, perfectionism, cognitive problems, social problems, oppositionality, and anxiousness/shyness. Test-retest reliability and internal consistency of the CTRS were found to be satisfactory and criterion validity of the measure as indexed by the ability of the measure to discriminate between children with and without attention deficit hyperactivity disorder was found to be good (68).

Strength and Difficulties Questionnaire (SDQ) - prosocial behaviour

The SDQ (98) is a questionnaire which in a self-report and a parent-report version covers domains of children's and adolescents' psychopathology (i. e. emotional symptoms conduct problems, hyperactivity-inattention, and peer problems) as well as personal strengths (i. e. prosocial behaviour). The prosocial behaviour subscale was found to have acceptable internal consistency with Cronbach's alpha ranging between .62 and .68 as well as good convergent and divergent validity (99).

Social Goal Scale (SGS)

The SGS (96) is a self-report scale on which adolescents can rate themselves concerning their social responsibility goals, their social relationship goals and their social status goals. Psychometric properties of the measure have not been investigated intensively, however, there is evidence that the internal consistency of the sub-scales of the measure is good with Cronbach's alpha ranging between .70 and .82 (96).

Dunning D, *et al. Evid Based Ment Health* 2022; 25:135–142. doi: 10.1136/ebmental-2022-300464

Student Externalizing Behaviour Screener (SEBS)

The SEBS (104) is a brief screening instrument for teachers to identify student with or at risk for behavioural disorders. The questionnaire was shown to be a reliable measure with concurrent validity and short-term predictive validity (105).

Symptoms Checklist-90-R (SCL-90-R)

The SCL-90-R (36) is a frequently used self-report measure for individuals aged 13 years and older that covers a broad range of mental health problems. Symptom dimensions of the measure are somatization, obsessive-compulsive, interpersonal sensitivity, depression, anxiety, hostility, phobic anxiety, paranoid ideation and psychoticism. Additionally, the scale has three global distress indices. Reliability, construct and criterion validity of the SCL-90-R were found to be high (37).

Teacher Engagement Report Form (TERF)

The TERF (38) is a measure in which teachers rate their students on aspects of affective, behavioural, and cognitive engagement. The TERF was found to have good internal consistency, external validity as measured by agreement of the TERF scores with student self-rating was overall acceptable but correlations were only moderate (39).

The Attitudes Toward Social Aggression Scale (ATSAS)

The ATSAS (106) is a self-report measure to assess attitudes towards violence and harassment among teenagers and includes cognitive, behavioural and affective dimensions. The questionnaire was developed in Spanish and presented good psychometric properties (106).

Behaviour Assessment System for Children, Second Edition (BASC-2)- parent rating scale

The BASC-2 (7) is an instrument for the evaluation of behaviour and self-perception of children, adolescents and young adults aged 2-25 that assesses a variety of problem behaviours, internalizing problems such as depression and anxiety, school problems, relations with peers and, adaptive skills. It consists of a self-report rating, a parent and a teacher rating scale. Internal consistency and test-retest reliability were found to be good with Cronbach's alpha ranging between .8 and .9 and r ranging between .7 and .9 (7).

Achenbach Youth Self-Report Scales (YSR)

The YSR (107) is a commonly self-report measure that comprises the following subscales: withdrawn, somatic complaints, anxious/depressed, social problems, thought problems, attention problems, delinquent behaviour, and aggressive behaviour. The measure has good internal consistency (108) and excellent convergence was found between the attention problems subscale with the ADHD diagnosis based on structured clinical interviews, as well as between the delinquent behaviour scale and the diagnosis of conduct disorder based on structured clinical interviews (109).

Measures of Executive Functions

A Developmental NEuroPSYchological Assessment (NEPSY II)

The NEPSY II (110) is a battery of tests designed to assess neuropsychological development including executive functioning, attention, visuo-spatial abilities and memory in children and adolescents aged 3 to 16 years old. The different subtests overall showed moderate to high test-retest reliability and good convergent validity (110).

ADD-H Comprehensive Teacher Rating Scale (ACTERS) – attention

The ACTERS (88) is a scale on which teachers can rate their students regarding to attention and hyperactive behaviour. Sub-scales of the measure are attention, hyperactivity, social skills and oppositional behaviour. The ACTERS was demonstrated to have high internal consistency and validity as a measure of attention deficit hyperactivity disorder (89).

Attention Network Task (ANT)

The ANT (111) is a widely used 30-min testing session designed to assess alerting, orienting and executive attention in children, adolescents and adults. In the ANT, efficiency of the three attentional networks is tested by measuring how response times are influenced by alerting cues, spatial cues, and flankers. The ANT was found to produce reliable outcomes (111) and to be useful in investigating differences in attention and executive functioning between children with and without attention deficit hyperactivity disorder (112).

Automated Operational Span Task (Ospan)

The Ospan (113) is a commonly used task for measuring working memory (WM) capacity which is mouse-driven and self-scoring, thus requiring little intervention on part of the experimenter. The task was shown to have good internal consistency (Cronbach's alpha = .78) as well as high test-retest reliability and was found to correlate highly with other measures of WM capacity (113). The Ospan can also be used to investigate WM capacity in children and adolescents (114, 115).

Avoidance and Fusion Questionnaire for Youth (AFQ-Y)

The AFQ-Y (116) is a self-report questionnaire that is designed to assess levels of cognitive fusion, experimental avoidance and resulting psychological inflexibility. The measure is based on concepts of acceptance and commitment therapy (ACT). The measure demonstrated adequate to good reliability, convergent and discriminant validity (117, 118).

Behaviour Rating Inventory of Executive Function (BRIEF) - parent

The BRIEF (118) is a questionnaire on which parents and teachers can rate children aged 5 to 18 regarding behavioral features of executive functioning. Internal consistency of both the parent and the teacher version was found to be high with Cronbach's alpha ranging between .80 and .98 and was found to correlate with other teacher/ parent report measures of executive functioning (119), however, findings on the correlation with performance-based measures of executive functioning are mixed (120).

Child Behaviour Checklist (CBCL)

The CBCL (9) is a parent-rating scale to assess various aspects of psychopathology and social competencies in childhood and adolescence (age groups 4-18). Internal consistency for the anxiously depressed and the aggressive behaviour subscale and test-retest reliability for the whole instrument

were found to be good to acceptable (10, 11). The CBCL demonstrated good criterion validity as defined by the ability to distinguish between individuals with and without psychopathology (12).

Children's Response Styles Questionnaire (CRSQ)

The CRSQ is a self-report questionnaire measuring children's responses to their own depressive symptoms (14). The questionnaire assesses to what extend respondents react to depressed mood with rumination, distraction and problem-solving. The questionnaire has good psychometric properties and there are high correlations between the rumination subscale and depressive symptoms (15).

Continuous Performance Tests (CPT)

CPTs (version: (121)) are computer-based vigilance task which is meant to assess how well individuals can inhibit responses and sustain attention. CPTs were overall found to be sensitive, reliable, and ecologically valid measures of inhibitory control and sustained attention in children and adolescents (122).

Corsi block forward

The Corsi block (123) is a widely used non-verbal paradigm to assess visou-spatial short-term and working memory (WM). In this test, subjects are required to point at blocks in the order they were presented before. The Corsi is a commonly used measure for children and adolescents and was found to correlate highly with WM facets of intelligence tests in youth (124).

Corsi block backward

The Corsi block backward (125) is an adaptation of the corsi block forward task in which subjects are required to point at blocks that where presented before in reversed order. The task is commonly used to test working memory (WM) in children and adolescents and was found to be less influenced by age than verbal WM backward recall tasks (125).

Decision-Making Skills (DMS)

The DMS (126) is a 4-item self-report test measuring decision-making ability in youth aged 11–18, with higher scores indicating a higher level of decision-making ability. Its psychometric properties are not yet established.

Digit span subtest - Wechsler Intelligence Scale for Children-Fourth Edition (WISC-IV)

The digit span subtest of the WISC-IV is a widely used test to measure working memory capacity in children and adolescents (127). It consists of the digit span forward subtest in which subjects have to repeat digits in the same order as they were read to them before and the digit span backward subtest in which subjects have to repeat digits that were read to them before in reverse order. Both subtests have strong psychometric properties (128).

Dimensional Change Card Sort (DCCS)

The DCCS (129) is a widely used performance-based measure of executive function in children. In the DCCS, children have to sort a series of bivalent test cards, first according to one dimension (e.g., color), and then according to the other (e.g., shape). The DDCS is commonly used to estimate the developmental stage of children's executive function as the ability to switch from one dimension to the other increases drastically with age (130).

The Conners Teacher Rating Scale (CTRS)

The CTRS (68) is a questionnaire on which teachers can assess children's behaviour in the classroom. The measure covers a range of facets of children's behaviour such as hyperactivity-impulsivity, perfectionism, cognitive problems, social problems, oppositionality, and anxiousness/shyness. Test-retest reliability and internal consistency of the CTRS were found to be satisfactory and criterion validity of the measure as indexed by the ability of the measure to discriminate between children with and without attention deficit hyperactivity disorder was found to be good (68).

Flanker Task (computerized)

The flanker task (131) is a widely used performance-based measure of inhibitory control for children. The flanker task has demonstrated excellent test-retest reliability as well as good convergent and discriminant validity (131).

Go/No-Go Task

The Go/No-Go Task (132) is a widely used performance-based measure of inhibitory control that is commonly used to assess this aspect of executive functioning in children. Performance of the task was found to correlate highly with measures of working memory capacity and general cognitive ability (133).

Head-Toes-Knees-Shoulders (HTKS)

The HTKS (134) is a performance-based measures for assessing executive function in children aged 3 to 6 years. It covers aspects of attentional or cognitive flexibility, working memory, and inhibitory control. The HTKS was found to have robust psychometric properties (134) and to correlate moderately with other performance-based measures of cognitive flexibility, working memory, and inhibitory control (135).

Hearts and Flowers Task (HF)

The HF (136) is a performance-based measure of executive function that can be administered to children up from an age of 4 years. In the task, subjects are asked to react to stimuli that are congruent and incongruent with certain categories as well as to switch between different rules. Performance (accuracy and reaction time) on the HF was found to predict academic outcomes in children (137).

Minnesota Executive Function Scale (MEFS)

The MEFS (138) is a performance-based measure of executive function for children aged 2 years and older. During the test, subjects are instructed to sort cards into one of two boxes on a screen according to an increasingly complex set of rules. Performance on the MEFS was found to correlate highly with other measures of cognitive abilities (139).

Peg tapping

The peg tapping task is a performance-based test of executive function in children aged three to six years (140). In the test, children have to act according to rules based on actions of the experimenter. The peg tapping task was found to have adequate test-retest reliability as well as good convergent and criterion validity (141).

Dunning D, *et al. Evid Based Ment Health* 2022; 25:135–142. doi: 10.1136/ebmental-2022-300464

Perception of Differences Test-Faces (FACES-R)

The FACES-R (142) was originally developed originally designed to measure perception of details and the ability to discriminate between objects, however, is also used assessing focused attention (143). Evidence on psychometric properties of the measure, especially for younger age groups, is currently still lacking.

Star Delay Task

The star delay task (144) is a behavioral measure for delay of gratification in children which has been adapted from the choice-delay task (145). As it is a recently developed measure, psychometric properties have not yet been established.

Stroop Test

The Stroop test (146) is one of the most widely used measures of executive function. It is designed to measure selective attention, inhibitory control and cognitive flexibility as in the ability to shift between different cognitive sets. In the task, participants are required to react to a series of color words according to the color in which the word is displayed which can be congruent or incongruent with the name of the color word. The Stroop test is commonly used to test executive function in children and adolescents and was shown to have good psychometric properties in these age groups (147).

Sustained Attention to response Task (SART)

The SART (148) is a performance-based measure of sustained attention. In this task, participants are required to withhold button pressing to an infrequent no-go stimulus. Failure to do so is scored an error of commission with more errors indicating poorer sustained attention. The SART is commonly used to measure sustained attention in children and adolescents and was found to discriminate between children with and without attention deficit hyperactivity disorder (149). Several different versions of the SART were used including an affective version where participants completed the task while listening to neutral or negatively aversive sounds.

Teacher-reported Attention Checklist

This attention checklist (150) is a scale on which teachers can rate their students' attentiveness in the classroom. There is preliminary evidence for the validity of the measure as the scores on the checklist were found to predict children's scores on performance-based attention tests (150).

Teacher Engagement Report Form (TERF)

The TERF (38) is a measure in which teachers rate their students on aspects of affective, behavioural, and cognitive engagement. The TERF was found to have good internal consistency, external validity as measured by agreement of the TERF scores with student self-rating was overall acceptable but correlations were only moderate (39).

Test Everyday Attention (TEA-Ch) – Children - Selective attention

The TEA-Ch (151) is a battery of performance-based tests designed to assess selective attention, divided attention, and attentional switching capacities in children and adolescents aged 6-16 years. The different subtests of the TEA-Ch were found to have high test-retest reliability and the subtests loading on the selective attention factor were found to correlate highly with other measures of selective attention (151).

Dunning D, *et al. Evid Based Ment Health* 2022; 25:135–142. doi: 10.1136/ebmental-2022-300464

d2 Test of Attention (d2)

The d2 (152) is a performance-based test involving simultaneous presentation of visually similar stimuli. The measure was found to be an internally consistent and valid measure of visual scanning accuracy and speed (152) and is also used for measuring attention in adolescents (153).

Behaviour Assessment System for Children, Second Edition (BASC-2)

The BASC-2 (7) is an instrument for the evaluation of behaviour and self-perception of children, adolescents and young adults aged 2-25 that assesses a variety of problem behaviours, internalizing problems such as depression and anxiety, school problems, relations with peers and, adaptive skills. It consists of a self-report rating, a parent and a teacher rating scale. Internal consistency and test-retest reliability were found to be good with Cronbach's alpha ranging between .8 and .9 and r ranging between .7 and .9 (7).

CNS Vital Signs (CNSVS)

The CNSVS (154) is a computerized neurocognitive test battery, which is commonly used as a clinical screening tool for children, adolescents and adults. It is based on established tests of executive function such as the Stroop test and comprises several subtests that assess processes such as verbal and visual memory, working memory capacity, selective attention, inhibitory control and attentional shifting. Psychometric properties of the CNSVS are good, similar to the single tests the battery is based on (154).

Strengths and Weaknesses of ADHD-Symptoms and Normal-Behavior scale (SWAN)

The SWAN (155) is a questionnaire on which parents can rate potential Attention Deficit Hyperactivity Disorder (ADHD) symptoms of their children on a dimensional scale. The SWAN appears to be a promising alternative to a categorical approach of assessing ADHD and allows to assess symptom levels in a more differentiated manner (156), however, it psychometric properties are not yet established.

Torrance Test of Creative Thinking (TTCT)

The TTCT (157) is a behavioral test that is designed to assess creativity. The TTCT was found to have moderate test–retest reliability and it has been argued that the TTCT does not capture every aspect of creativity and own its own might not be a valid measure of creativity (158).

Tower of London Test (TLT)

The TLT (159) is a widely used performance-based measure of planning and problem solving. In the test, subjects are instructed to rearrange objects so that the new configuration corresponds to a pattern presented on a stimulus card and are required to achieve this in a limited number of moves. There are various variations of the test including adaptations for children and the task was shown to be a reliable and valid measure of executive function in children (160).

Trail making task (TMT)

The TMT (161) is a widely used performance based measure of visual attention and task switching (cognitive flexibility). It is commonly used to measure these executive functions in children and adolescents and demonstrated good psychometric properties in these age groups (162).

Digit Span Subtest (Backward) - Wechsler Intelligence Scale for Children-Fourth Edition (WISC-IV)

The digit span subtest of the WISC-IV is a widely used test to measure working memory capacity in children and adolescents (127). It consists of the digit span forward subtest in which subjects have to repeat digits in the same order as they were read to them before and the digit span backward subtest in which subjects have to repeat digits that were read to them before in reverse order. Both subtests have strong psychometric properties (128).

Complex Span Task

Complex span tasks (163) are performance-based measures of working memory capacity that are designed to measure both processing and storage aspects of working memory. In these tasks, subjects have to engage in activities such as reading sentences or mental arithmetic, and simultaneously maintain certain aspects of this processing for subsequent recall. Complex span tasks were shown to be suitable measures of working memory capacity in children, adolescents and adults (164).

N-Back Task

The n-back task (165) is a commonly used performance-based measure of visuo-spatial working memory. In a continuous recognition task, participants must decide whether a stimulus was previously presented in certain conditions. The n-back task is commonly used to assess working memory in children and adolescents, however, evidence on psychometric properties in adults and adolescents is mixed (166, 167).

Achenbach Youth Self-Report Scales (YSR)

The YSR (107) is a commonly self-report measure that comprises the following subscales: withdrawn, somatic complaints, anxious/depressed, social problems, thought problems, attention problems, delinquent behaviour, and aggressive behaviour. The measure has good internal consistency (108) and excellent convergence was found between the attention problems subscale with the ADHD diagnosis based on structured clinical interviews, as well as between the delinquent behaviour scale and the diagnosis of conduct disorder based on structured clinical interviews (109).

Measures of Attention

ADD-H Comprehensive Teacher Rating Scale (ACTERS) – attention

The ACTERS (88) is a scale on which teachers can rate their students regarding to attention and hyperactive behaviour. Sub-scales of the measure are attention, hyperactivity, social skills and oppositional behaviour. The ACTERS was demonstrated to have high internal consistency and validity as a measure of attention deficit hyperactivity disorder (89).

Attention Network Task (ANT)

The ANT (111) is a widely used 30-min testing session designed to assess alerting, orienting and executive attention in children, adolescents and adults. In the ANT, efficiency of the three attentional networks is tested by measuring how response times are influenced by alerting cues, spatial cues, and flankers. The ANT was found to produce reliable outcomes (111) and to be useful in investigating differences in attention and executive functioning between children with and without attention deficit hyperactivity disorder (112).

Child Behaviour Checklist (CBCL)

The CBCL (9) is a parent-rating scale to assess various aspects of psychopathology and social competencies in childhood and adolescence (age groups 4-18). Internal consistency for the anxiously depressed and the aggressive behaviour subscale and test-retest reliability for the whole instrument were found to be good to acceptable (10, 11). The CBCL demonstrated good criterion validity as defined by the ability to distinguish between individuals with and without psychopathology (12).

Children's Response Styles Questionnaire (CRSQ)

The CRSQ is a self-report questionnaire measuring children's responses to their own depressive symptoms (14). The questionnaire assesses to what extend respondents react to depressed mood with rumination, distraction and problem-solving. The questionnaire has good psychometric properties and there are high correlations between the rumination subscale and depressive symptoms (15).

Continuous Performance Tests (CPT)

CPTs (version: (121)) are computer-based vigilance task which is meant to assess how well individuals can inhibit responses and sustain attention. CPTs were overall found to be sensitive, reliable, and ecologically valid measures of inhibitory control and sustained attention in children and adolescents (122).

The Conners Teacher Rating Scale (CTRS)

The CTRS (68) is a questionnaire on which teachers can assess children's behaviour in the classroom. The measure covers a range of facets of children's behaviour such as hyperactivity-impulsivity, perfectionism, cognitive problems, social problems, oppositionality, and anxiousness/shyness. test-retest reliability and internal consistency of the CTRS were found to be satisfactory and criterion validity of the measure as indexed by the ability of the measure to discriminate between children with and without attention deficit hyperactivity disorder was found to be good (68).

Flanker Task (computerized)

The flanker task (131) is a widely used performance-based measure of inhibitory control for children. The flanker task has demonstrated excellent test-retest reliability as well as good convergent and discriminant validity (131).

Go/No-Go Task

The Go/No-Go Task (132) is a widely used performance-based measure of inhibitory control that is commonly used to assess this aspect of executive functioning in children. Performance of the task was found to correlate highly with measures of working memory capacity and general cognitive ability (133).

Sustained Attention to response Task (SART)

The SART (148) is a performance-based measure of sustained attention. In this task, participants are required to withhold button pressing to an infrequent no-go stimulus. Failure to do so is scored an error of commission with more errors indicating poorer sustained attention. The SART is commonly used to measure sustained attention in children and adolescents and was found to discriminate between children with and without attention deficit hyperactivity disorder (149).

Teacher Engagement Report Form (TERF)

The TERF (38) is a measure in which teachers rate their students on aspects of affective, behavioural, and cognitive engagement. The TERF was found to have good internal consistency, external validity as measured by agreement of the TERF scores with student self-rating was overall acceptable but correlations were only moderate (39).

Teacher-reported Attention Checklist

This attention checklist (150) is a scale on which teachers can rate their students' attentiveness in the classroom. There is preliminary evidence for the validity of the measure as the scores on the checklist were found to predict children's scores on performance-based attention tests (150).

Test Everyday Attention (TEA-Ch) – Children - Selective attention

The TEA-Ch (151) is a battery of performance-based tests designed to assess selective attention, divided attention, and attentional switching capacities in children and adolescents aged 6-16 years. The different subtests of the TEA-Ch were found to have high test-retest reliability and the subtests loading on the selective attention factor were found to correlate highly with other measures of selective attention (151).

d2 Test of Attention (d2)

The d2 (152) is a performance-based test involving simultaneous presentation of visually similar stimuli. The measure was found to be an internally consistent and valid measure of visual scanning accuracy and speed (152) and is also used for measuring attention in adolescents (153).

Behaviour Assessment System for Children, Second Edition (BASC-2)

The BASC-2 (7) is an instrument for the evaluation of behaviour and self-perception of children, adolescents and young adults aged 2-25 that assesses a variety of problem behaviours, internalizing problems such as depression and anxiety, school problems, relations with peers and, adaptive skills. It consists of a self-report rating, a parent and a teacher rating scale. Internal consistency and test-retest reliability were found to be good with Cronbach's alpha ranging between .8 and .9 and r ranging between .7 and .9 (7).

Achenbach Youth Self-Report Scales (YSR)

The YSR (107) is a commonly self-report measure that comprises the following subscales: withdrawn, somatic complaints, anxious/depressed, social problems, thought problems, attention problems,

Dunning D, *et al. Evid Based Ment Health* 2022; 25:135–142. doi: 10.1136/ebmental-2022-300464

delinquent behaviour, and aggressive behaviour. The measure has good internal consistency (108) and excellent convergence was found between the attention problems subscale with the ADHD diagnosis based on structured clinical interviews, as well as between the delinquent behaviour scale and the diagnosis of conduct disorder based on structured clinical interviews (109).

Supplement D

Hierarchy of outcome measures

The hierarchies of outcome measures were created based on the following criteria. (A) Theoretical fit between the outcome and the construct the measure was designed to assess; (B) Frequency of use of the measure in general and especially in children and adolescent populations; (C) Psychometric properties of the measure, especially in children and adolescent samples; (D) Existing hierarchies of measurement scales from earlier meta-analyses, e.g.. (44); (E) Appropriateness of measure modality for assessing the outcome – for internalizing symptoms and inner processes self-report measures were generally ranked highest (45), for externalizing symptoms concerning aspects of negative and social behaviour parent- and teacher-report measures were mostly ranked highest (46) and for executive functions and attention performance-based measures were generally ranked highest (47,48).

Note: If a measure has separate scales it can appear in more than one hierarchy. For example the Strength and Difficulties Questionnaire has scales relating to both social and negative behaviour.

Hierarchy of Depression Measures

Hierarchy of depression symptom severity measurement scales and abbreviations		
1	Beck Depression Inventory-II	BDI
2	Children’s Depression Inventory	CDI
3	Beck Youth Depression & Anxiety Scales	BYI
4	Revised Child Anxiety and Depression Scale	RDACS
5	Patient Health Questionnaire-9	PHQ-9
6	The Short Mood and Feelings Questionnaire	SMFQ
7	Symptoms Checklist-90-R	SCL-90-R
8	Depression Anxiety Stress Scale - Depression	DASS
9	Centre for Epidemiologic Studies Depression Scale-Revised	CESD-R
10	Behavior Assessment System for Children, Second Edition	BASC-2
11	Children’s Response Styles Questionnaire	CRSQ
12	Response Styles Questionnaire	RSQ
13	Child Behaviour Checklist	CBCL
14	Fragebogen zur Erfassung der gesundheitsbezogenen Lebensqualität von Kindern und Jugendlichen	KINDL
15	Positive and Negative Suicide Ideation	PANSI
16	Rosenberg's Self-Esteem Scale	RSES
17	Differential Emotions Scale	DES
18	The Child PTSD Symptom Scale	CPSS
19	Teacher Engagement Report Form	TERF
20	Seattle Personality Questionnaire	SPQ
21	Depression QUESTIONNAIRE NOT DESCRIBED	
22	Rumination QUESTIONNAIRE NOT DESCRIBED	
23	Emotion Awareness (Sadness) QUESTIONNAIRE NOT DESCRIBED	

Hierarchy of Anxiety measures

**Hierarchy of anxiety symptom severity and stress measurement scales and abbreviations**		
1	Beck Youth Depression & Anxiety Scales	BYI
2	Revised Child Anxiety and Depression Scale	RDACS
3	General Anxiety Disorder-7 Questionnaire	GAD-7
4	State Trait Anxiety Inventory for Children	STAIC
5	Multidimensional Anxiety Scale for Children	MASC
6	Symptoms Checklist-90-R	SCL-90-R
7	Screen for Child Anxiety Related Emotional Disorders	SCARED
8	Depression Anxiety Stress Scale	DASS
9	The Revised Children's Manifest Anxiety Scale	RMACS
10	Behavior Assessment System for Children, Second Edition	BASC-2
11	Social Anxiety Scale for Children – Revised	SASC-R
12	Adolescent Stress Questionnaire	ASQ
13	Test Anxiety Scale	TAS
14	Perceived Stress Scale	PSS
15	Child Behaviour Checklist	CBCL
16	The Conners Teacher Rating Scale	CTRS
17	Relationship Scales Questionnaire	RSQ
18	Differential Emotions Scale	DES
19	Indices de détresse psychologique – Enquête Santé Québec	IDPESQ-14
20	The Child PTSD Symptom Scale	CPSS
21	Emotion Awareness (Sadness) QUESTIONNAIRE NOT DESCRIBED	

Hierarchy of Wellbeing measures

Hierarchy of wellbeing measurement scales and abbreviations		
1	Warwick-Edinburgh Mental Wellbeing Scale	WEMWBS
2	Stirling Children's Wellbeing Scale	SCWBS
3	Student Subjective Wellbeing Questionnaire	SSWQ
4	Fragebogen zur Erfassung der gesundheitsbezogenen Lebensqualität von Kindern und Jugendlichen	KINDL

Hierarchy of Mindfulness Measures

Hierarchy of mindfulness measurement scales and abbreviations		
1	Children Adolescents Mindfulness Measure	CAMM
2	Mindful Attention Awareness Scale	MAAS
3	Comprehensive Inventory of Mindfulness Experiences - Adolescents	CHIME-A
4	Self-Compassion Scale	SCS
5	Cognitive and Affective Mindfulness Scale	CAMS
6	Mindfulness (Observe) QUESTIONNAIRE NOT DESCRIBED	
7	Mindfulness (Accept without judgement) QUESTIONNAIRE NOT DESCRIBED	
8	Mindfulness (Act with awareness) QUESTIONNAIRE NOT DESCRIBED	

Hierarchy of Social Behaviour measures

Hierarchy of social behaviour measurement scales and abbreviations		
1	Behaviour Assessment System for Children, Second Edition	BASC-2
2	ADD-H Comprehensive Teacher Rating Scale	ACTERS
3	Interpersonal Reactivity Index	IRI
4	Symptoms Checklist-90-R	SCL-90-R
5	Social Goal Scale	SGS
6	Strength and Difficulties Questionnaire	SDQ
7	Teacher Engagement Report Form	TERF
8	Teacher-Rated Social Competence Scale	TSC
9	The Social Connectedness Scale - Revised	
10	Socio-Cultural Attitudes Towards Appearance Scale	SATAQ
11	Clinical Impairment Assessment	CIA
12	Differential Emotions Scale	DES
13	Attachment Story Completion Task	ASCT
14	Sharing task (own test)	
15	Empathic Responding (own measure)	
16	Emotion Awareness (Shyness) QUESTIONNAIRE NOT DESCRIBED	

Dunning D, *et al. Evid Based Ment Health* 2022; 25:135–142. doi: 10.1136/ebmental-2022-300464

Hierarchy of negative behaviour measures

Hierarchy of negative behaviour measurement scales and abbreviations		
1	Behaviour Assessment System for Children, Second Edition - parent rating scale	BASC-2
2	Student Externalizing Behaviour Screener	SEBS
3	Strength and Difficulties Questionnaire	SDQ
4	The Conners Teacher Rating Scale	CTRS
5	Child Behaviour Checklist	CBCL
6	Teacher Engagement Report Form	TERF
7	Achenbach Youth Self-Report Scales	YSR
8	Aggression Scale	AS
9	The Attitudes Toward Social Aggression Scale	ATSAS
10	Symptoms Checklist-90-R	SCL-90-R
11	Social Goal Scale	SGS
12	Behaviour regulation (3rd person observations)	
13	Differential Emotions Scale	DES
14	The Child PTSD Symptom Scale	CPSS
15	Emotion Awareness QUESTIONNAIRE NOT DESCRIBED	

Dunning D, *et al. Evid Based Ment Health* 2022; 25:135–142. doi: 10.1136/ebmental-2022-300464

Hierarchy of executive function measures

Hierarchy of executive functions measures and abbreviations		
1	Stroop Test	
2	Tower of London Test	TLT
3	Flanker Task (computerized)	
4	Go/No-Go Task	
5	Dimensional Change Card Sort	DCCS
6	Digit Span Subtest - Wechsler Intelligence Scale for Children-Fourth Edition (WISC-IV)	DS (WISC-IV)
7	Corsi Block Forward	
8	Corsi block Backward	
9	CNS Vital Signs	CNSVS
10	Minnesota Executive Function Scale	MEFS
11	DKEFS Trail Making Task	TMT
12	Attention Network Task	ANT
13	Automated Operational Span Task	Ospan
14	Head-Toes-Knees-Shoulders	HTKS
15	Hearts and Flowers Task	HF
16	Peg tapping	
17	Continuous Performance Tests	CPT
18	Complex Span Task	
19	Sustained Attention to response Task	SART
20	A Developmental NEuroPSYchological Assessment	NEPSY II
21	d2 Test of Attention	d2
22	Test Everyday Attention	TEA-Ch
23	N-Back Task	
24	Star Delay Task	
25	Behaviour Rating Inventory of Executive Function - parent	BRIEF
26	Behaviour Assessment System for Children, Second Edition	BASC-2
27	Achenbach Youth Self-Report Scales	YSR
28	ADD-H Comprehensive Teacher Rating Scale – attention	ACTERS

29	Strengths and Weaknesses of ADHD-Symptoms and Normal-Behavior scale	SWAN
30	Perception of Differences Test-Faces	FACES-R
31	Torrance Test of Creative Thinking	TTCT
32	Teacher Engagement Report Form	TERF
33	The Conners Teacher Rating Scale	CTRS
34	Child Behaviour Checklist	CBCL
35	Teacher-reported Attention Checklist	
36	Decision-Making Skills	DMS
37	Children’s Response Styles Questionnaire	CRSQ
38	Avoidance and Fusion Questionnaire for Youth	AFQ-Y

Hierarchy of attention measures

Hierarchy of attention measures and abbreviations		
1	Attention Network Task	ANT
2	Continuous Performance Tests	CPT
3	Flanker Task (computerized)	
4	Go/No-Go Task	
5	Sustained Attention to response Task	SART
6	d2 Test of Attention	d2
7	Test Everyday Attention	TEA-Ch
8	ADD-H Comprehensive Teacher Rating Scale	ACTERS
9	Behaviour Assessment System for Children, Second Edition	BASC-2
10	The Conners Teacher Rating Scale	CTRS
11	Teacher Engagement Report Form	TERF
12	Teacher-reported Attention Checklist	
13	Child Behaviour Checklist	CBCL
14	Achenbach Youth Self-Report Scales	YSR
15	Children’s Response Styles Questionnaire	CRSQ

Supplement E

Risk of Bias

Method

Two authors (MV, JMM) independently graded risk-of-bias in the following seven categories: 1) bias arising from the randomisation process; 2) bias due to deviations from intended interventions; 3) bias due to missing outcome data; 4) bias in the measurement of the outcome; 5) bias in the selection of the reported result; 6) other bias (allegiance effects); 7) overall bias. Each risk of bias category for each study was given one of three ratings: low risk, high risk, or some concerns. After all studies were independently rated, consensus was reached through discussion. The category measuring Allegiance effects was graded slightly differently, with bias rated as 'yes', 'probably yes', 'probably no' and 'no'; the 'yes' rating was recorded as a high risk of bias, the 'no' rating a low risk of bias and the 'probably yes' and 'probably no' ratings as some concerns of bias.

To enable the use of risk of bias as a potential moderator of the effects of MBPs, a discrete variable was calculated based on the first six risk of bias categories, with each high risk of bias given a value of 1, each some concerns of bias given a value of 0 and each low lisk of bias a value of -1. Therefore, individual studies could have a risk of bias score of between -6 and 6, with higher scores indicating a higher risk of bias.

The summary Risk of Bias 2.0 assessment of each study

	Randomisation process	Deviations from intended interventions	Missing outcome data	Measurement of the outcome	Selection of the reported result	Other bias: allegiancy effects	Overall bias
Abedini, et al., (2020)	Some concerns	Low	Low	Low	Some concerns	Probably yes	Some concerns
Alampay, et al., (2020)	High	Some concerns	Some concerns	Some concerns	Some concerns	Yes	Some concerns
Atkinson & Wade (2015)	Some concerns	Some concerns	Some concerns	Some concerns	Some concerns	No	Some concerns
Barnes et al., (2016)	Some concerns	Some concerns	Low	Some concerns	Some concerns	Yes	High
Biegal et al., (2009)	Low	Low	Low	Some concerns	Some concerns	No	Some concerns
Bluth et al., (2015)	Some concerns	Some concerns	Low	Some concerns	Some concerns	No	Some concerns
Britton et al., (2014)	Some concerns	Some concerns	Some concerns	Some concerns	Some concerns	No	Some concerns
Chadli et al., (2016)	Some concerns	Low	Some concerns	Low	Some concerns	Yes	Some concerns
Cohen et al., (2021)	High	Low	Some concerns	Some concerns	Low	Probably no	Some concerns
Cresentini et al., (2016)	Some concerns	Some concerns	Low	Some concerns	Some concerns	Probably yes	Some concerns
De Voy (2018)1	Some concerns	Some concerns	Some concerns	Some concerns	Some concerns	Probably no	Some concerns
De Voy (2018)2	Some concerns	Some concerns	Some concerns	Some concerns	Some concerns	Probably no	Some concerns
De Voy (2018)3	Some concerns	Some concerns	Some concerns	Some concerns	Some concerns	Probably no	Some concerns
Delgado-Suárez et al., (2021)	Low	Low	Low	Low	Some concerns	Yes	Some concerns
Desmond & Hanich (2010)	Some concerns	Some concerns	Some concerns	Some concerns	Some concerns	No	Some concerns
Devcich et al., (2017)	Some concerns	Some concerns	Some concerns	Low	High	Yes	High
Díaz-González, et al. (2018)	Some concerns	Some concerns	High	Some concerns	Some concerns	Probably no	High
Dunning et al., (in prep)	Low	Low	High	Low	Low	No	Some concerns
Flook et al. (2010)	Some concerns	Low	Low	Some concerns	Some concerns	Probably yes	Some concerns
Flook et al. (2015)	Some concerns	Some concerns	Some concerns	Some concerns	Some concerns	Yes	Some concerns

Dunning D, *et al. Evid Based Ment Health* 2022; 25:135–142. doi: 10.1136/ebmental-2022-300464

The summary Risk of Bias 2.0 assessment of each study

	Randomisation process	Deviations from intended interventions	Missing outcome data	Measurement of the outcome	Selection of the reported result	Other bias: allegiancy effects	Overall bias
Franco et al. (2010)	Some concerns	Low	Some concerns	Some concerns	Some concerns	Yes	Some concerns
Franco Justo (2009)	Some concerns	Some concerns	Some concerns	Some concerns	Some concerns	Yes	Some concerns
Frank, et al., (2021)	Low	Low	Low	Some concerns	Some concerns	Probably no	Some concerns
Fung, et al., (2019)	Some concerns	Some concerns	Some concerns	Some concerns	Some concerns	Probably no	Some concerns
Ghiroldi, et al., (2020)	Some concerns	Low	Low	Some concerns	Some concerns	Probably no	Some concerns
Gregoski et al. (2011)	Some concerns	Low	Low	Some concerns	Low	Yes	Some concerns
Himelstein et al. (2015)	Some concerns	Some concerns	Some concerns	Some concerns	High	Yes	High
Ho, et al., (2021)	Some concerns	Some concerns	Some concerns	Some concerns	Low	No	Some concerns
Johnson et al. (2016)	Some concerns	Low	Some concerns	Some concerns	Low	No	Some concerns
Johnson et al. (2017)	Some concerns	Low	Some concerns	Some concerns	Low	No	Some concerns
Kiani, et al., (2017)	Some concerns	Some concerns	Low	Some concerns	Some concerns	Yes	Some concerns
Koncz et al., (2021)	Some concerns	High	Some concerns	Some concerns	Some concerns	Yes	Some concerns
Kuyken, et al. (in prep)	Low	Low	Low	Some concerns	Low	No	Some concerns
Lam & Seiden (2019)	Some concerns	Low	Low	Some concerns	Some concerns	No	Some concerns
Lassander, et al., (2020)	Low	Some concerns	Some concerns	Some concerns	Low	No	Some concerns
Lassander, et al., (2021)	Low	Some concerns	Some concerns	Some concerns	Some concerns	No	Some concerns
Lawler & Esposito (2019)	Some concerns	Low	Some concerns	Low	Low	Probably yes	Some concerns
Leonard et al. (2013)	Some concerns	High	Some concerns	Low	Some concerns	Yes	High
Liehr & Diaz (2010)	Some concerns	Some concerns	Some concerns	Some concerns	Some concerns	No info	Some concerns

Dunning D, et al. Evid Based Ment Health 2022; 25:135–142. doi: 10.1136/ebmental-2022-300464

The summary Risk of Bias 2.0 assessment of each study

	randomisation process	deviations from intended interventions	missing outcome data	measurement of the outcome	selection of the reported result	Other bias: allegiancy effects	Overall bias
Lo, et al., (2017)	Low	Some concerns	Some concerns	Some concerns	Low	Yes	Some concerns
Long, et al., (2018)	High	Some concerns	Some concerns	Some concerns	Some concerns	Yes	High
Lu, et al., (2019)	High	Some concerns	Some concerns	Some concerns	Some concerns	Yes	High
Malboeuf-Hurtubise, et al., (2019)	High	Low	Low	Some concerns	Some concerns	Yes	High
Moreno-Gomez & Cejudo (2019)	High	Some concerns	Some concerns	Some concerns	Some concerns	Probably no	High
Napoli et al. (2005)	Some concerns	Some concerns	Low	Some concerns	Some concerns	Probably yes	Some concerns
Parker et al. (2014)	some concerns	High	High	high	Some concerns	Probably yes	High
Poehlmann-Tynan et al. (2016)	Some concerns	Some concerns	Some concerns	Low	Some concerns	No	Some concerns
Quach et al. (2015)	Some concerns	Some concerns	Some concerns	Low	Some concerns	Probably yes	Some concerns
Rawlett et al., (2019)	Some concerns	Some concerns	Some concerns	Some concerns	Some concerns	No	Some concerns
Ricarte et al. (2015)	Some concerns	Low	Low	Some concerns	Some concerns	No	Some concerns
Schonert-Reichl et al. (2015)	Some concerns	Low	Low	Low	Some concerns	Probably yes	Some concerns
Semple et al. (2010)	Low	Low	Low	Some concerns	High	No	Some concerns
Shirk et al. (2014)	Some concerns	Low	Low	Some concerns	Some concerns	Probably yes	Some concerns
Shomaker et al. (2017)	Some concerns	Some concerns	Low	Some concerns	Some concerns	No	Some concerns
Shomaker et al. (2019)	Some concerns	Low	Low	Low	Some concerns	No	Some concerns
Sibinga et al. (2013)	Low	Low	Low	Some concerns	Some concerns	No	Some concerns
Sibinga et al. (2015)	Low	Some concerns	Some concerns	Some concerns	Some concerns	Probably no	Some concerns
Siffredi et al (2021)	Low	Low	Low	Some concerns	Some concerns	Yes	Some concerns
Solar (2018)	Some concerns	High	Some concerns	Some concerns	Some concerns	Probably yes	High

The summary Risk of Bias 2.0 assessment of each study

	randomisation process	deviations from intended interventions	missing outcome data	measurement of the outcome	selection of the reported result	Other bias: allegiancy effects	Overall bias
Tan & Martin (2014)	Some concerns	Low	High	Some concerns	Some concerns	Yes	High
Thomas & Atkinson (2016)	Some concerns	High	Some concerns	Some concerns	Some concerns	Probably no	High
Viglas & Perlman (2018)	Some concerns	Some concerns	Some concerns	Some concerns	Some concerns	Probably yes	Some concerns
Vohra et al., (2019)	Some concerns	Low	Some concerns	Some concerns	Low	Probably no	Some concerns
Volanen, et al., (2020)	Low	Low	Low	Some concerns	Some concerns	No	Some concerns
Wright et al., (2019)	Some concerns	Low	Low	Some concerns	Some concerns	Probably no	Some concerns
Zelazo et al., (2018)	Some concerns	Some concerns	Some concerns	Some concerns	Some concerns	Probably yes	Some concerns

Key

(red)	High risk of bias
(yellow)	Some concerns of risk of bias
(green)	Low risk of bias

Dunning D, *et al. Evid Based Ment Health* 2022; 25:135–142. doi: 10.1136/ebmental-2022-300464

Supplement F

The Grading of Recommendations, Assessment, Development and Evaluation (GRADE) Framework

Author(s): Dunning et al. 2022
Question: Mindfulness compared to active controls for improving cognitive skills, behavior and mental health of children and adolescents
Setting: Schools

Certainty assessment							№ of participants		Effect		Certainty	Importance
№ of studies	Study design	Risk of bias	Inconsistency	Indirectness	Imprecision	Other considerations	Mindfulness	TUE	Relative (95% CI)	Absolute (95% CI)		
**Anxiety/Stress (post-test; assessed with questionnaire)**												
17	RCTs	not double-blind	not serious	not serious	not serious	none	809	896	-	SMD **0.11 SD lower** (0.02 lower to 0.21 lower)	⨁⨁⨁◯ MODERATE	strong
**Attention (post-test; assessed with questionnaire and/or task)**												
11	RCTs	not double-blind	not serious	not serious	serious	none	763	740	-	SMD **0.04 SD higher** (-0.06 lower to 0.14 higher)	⨁⨁◯◯ LOW	weak
**Depression (post-test; assessed with questionnaire)**												
13	RCTs	not double-blind	serious	not serious	not serious	none	3045	2957	-	SMD **0.10 SD lower** (0.04 lower to 0.23 lower)	⨁⨁◯◯ LOW	strong
**Executive Functions (post-test; assessed with questionnaire and/or task)**												
14	RCTs	not double-blind	not serious	not serious	serious	none	947	940	-	SMD **0.04 SD higher** (-0.06 lower to 0.13 higher)	⨁⨁◯◯ LOW	weak
**Mindfulness (post-test; assessed with questionnaire)**												
14	RCTs	not double-blind	serious	not serious	not serious	none	778	850	-	SMD **0.24 SD higher** (0.06 higher to 0.42 higher)	⨁⨁◯◯ LOW	strong
**Negative Behaviour (post-test; assessed with questionnaire and some observational)**												
8	RCTs	not double-blind	serious	not serious	serious	none	525	520	-	SMD **0.13 SD lower** (-0.06 higher to 0.32 lower)	⨁◯◯◯ VERY LOW	weak
**Social Behaviour (post-test; assessed with questionnaire and a few observational)**												
8	RCTs	not double-blind	very serious	not serious	serious	none	637	620	-	SMD **-0.03 SD lower** (-0.27 lower to 0.22 higher)	⨁◯◯◯ VERY LOW	weak
**Wellbeing (post-test; assessed with questionnaire)**												
7	RCTs	not double-blind	serious	not serious	serious	none	1571	1600	-	SMD **0.11 SD higher** (-0.04 lower to 0.25 higher)	⨁◯◯◯ VERY LOW	weak

RCTs: Randomized Controlled Trials. **CI:** Confidence interval; **SMD:** Standardized mean difference

Author(s): Dunning et al. 2022
Question: Mindfulness compared to passive controls for improving cognitive skills, behavior and mental health of children and adolescents
Setting: Schools

Certainty assessment							№ of participants		Effect		Certainty	Importance
№ of studies	Study design	Risk of bias	Inconsistency	Indirectness	Imprecision	Other considerations	Mindfulness	TUE	Relative (95% CI)	Absolute (95% CI)		
**Anxiety/Stress (post-test; assessed with questionnaire)**												
21	RCTs	not double-blind	serious	not serious	not serious	publication bias suspected	5040	4990	-	SMD **0.12 SD lower** (0.01 lower to 0.38 lower)	⨁◯◯◯ VERY LOW	weak
**Attention (post-test; assessed with questionnaire and/or task)**												
14	RCTs	not double-blind	very serious	not serious	not serious	none	781	654	-	SMD **0.35 SD higher** (0.11 lower to 0.58 lower)	⨁⨁◯◯ LOW	strong
**Depression (post-test; assessed with questionnaire)**												
19	RCTs	not double-blind	serious	not serious	serious	none	7361	5592	-	SMD **0.06 SD lower** (-0.02 higher to 0.13 lower)	⨁◯◯◯ VERY LOW	weak
**Executive Functions (post-test; assessed with questionnaire and/or task)**												
25	RCTs	not double-blind	very serious	not serious	not serious	publication bias suspected	5003	4887	-	SMD **0.35 SD higher** (0.19 higher to 0.52 higher)	⨁◯◯◯ VERY LOW	weak
**Mindfulness (post-test; assessed with questionnaire)**												
12	RCTs	not double-blind	serious	not serious	serious	publication bias suspected	4522	4533	-	SMD **0.03 SD higher** (-0.11 lower to 0.16 higher)	⨁◯◯◯ VERY LOW	weak
**Negative Behaviour (post-test; assessed with questionnaire and a few observational)**												
17	RCTs	not double-blind	very serious	not serious	not serious	publication bias suspected potential dose response	4798	4664	-	SMD **0.23 SD lower** (0.08 lower to 0.37 lower)	⨁⨁◯◯ LOW	strong
**Social Behaviour (post-test; assessed with questionnaire and a few observational/grade)**												
11	RCTs	not double-blind	very serious	not serious	not serious	publication bias suspected	4525	5390	-	SMD **0.21 SD higher** (0.04 higher to 0.39 higher)	⨁◯◯◯ VERY LOW	weak
**Wellbeing (post-test; assessed with questionnaire)**												
6	RCTs	not double-blind	serious	not serious	serious	Potential inverse dose response	5463	4631	-	SMD **-0.10 SD lower** (-0.20 lower to 0.01 higher)	⨁◯◯◯ VERY LOW	weak

RCTs: Randomized Controlled Trials. **CI:** Confidence interval; **SMD:** Standardized mean difference

 BMJ Publishing Group Limited (BMJ) disclaims all liability and responsibility arising from any reliance placed on this supplemental material which has been supplied by the author(s)

Author(s): Dunning et al. 2022
Question: Selective mindfulness for improving cognitive skills, behavior and mental health of children and adolescents
Setting: Schools

Certainty assessment							№ of participants		Effect		Certainty	Importance
№ of studies	Study design	Risk of bias	Inconsistency	Indirectness	Imprecision	Other considerations	Mindfulness	TUE	Relative (95% CI)	Absolute (95% CI)		
**Anxiety/Stress (post-test; assessed with questionnaire)**												
18	RCTs	not double-blind	low	not serious	not serious	none	539	593	-	SMD **0.24 SD lower** (0.11 lower to 0.37 lower)	⨁⨁⨁◯ MODERATE	strong
**Attention (post-test; assessed with questionnaire and/or task)**												
9	RCTs	not double-blind	very serious	not serious	serious	none	394	369	-	SMD **0.34 SD higher** (-0.01 lower to 0.66 lower)	⨁◯◯◯ VERY LOW	weak
**Depression (post-test; assessed with questionnaire)**												
16	RCTs	not double-blind	serious	not serious	not serious	none	478	468	-	SMD **0.21 SD lower** (0.004 lower to 0.42 lower)	⨁⨁◯◯ LOW	strong
**Executive Functions (post-test; assessed with questionnaire and/or task)**												
13	RCTs	not double-blind	serious	not serious	not serious	none	495	471	-	SMD **0.39 SD higher** (0.14 higher to 0.65 higher)	⨁⨁◯◯ LOW	strong
**Mindfulness (post-test; assessed with questionnaire)**												
11	RCTs	not double-blind	not serious	not serious	not serious	none	252	236	-	SMD **0.29 SD higher** (0.09 higher to 0.50 higher)	⨁⨁⨁◯ MODERATE	strong
**Negative Behaviour (post-test; assessed with questionnaire)**												
12	RCTs	not double-blind	not serious	not serious	serious	none	443	442	-	SMD **0.10 SD lower** (-0.02 higher to 0.22 lower)	⨁⨁◯◯ LOW	weak
**Social Behaviour (post-test; assessed with questionnaire and a few observational)**												
7	RCTs	not double-blind	serious	not serious	serious	none	256	314	-	SMD -**0.11 SD lower** (-0.47 lower to 0.24 higher)	⨁◯◯◯ VERY LOW	weak
**Wellbeing (post-test; assessed with questionnaire)**												
1	RCTs	not double-blind	-	not serious	serious	only one study	22	49	-	SMD -**0.61 SD lower** (-1.12 lower to -0.10 lower)	⨁◯◯◯ VERY LOW	weak

RCTs: Randomized Controlled Trials, **CI:** Confidence interval; **SMD:** Standardized mean difference

Author(s): Dunning et al. 2022
Question: Universal mindfulness for improving cognitive skills, behavior and mental health of children and adolescents
Setting: Schools

Certainty assessment							№ of participants		Effect		Certainty	Importance
№ of studies	Study design	Risk of bias	Inconsistency	Indirectness	Imprecision	Other considerations	Mindfulness	TUE	Relative (95% CI)	Absolute (95% CI)		
**Anxiety/Stress (post-test; assessed with questionnaire)**												
18	RCTs	not double-blind	serious	not serious	serious	publication bias suspected	5256	5142	-	SMD **0.08 SD lower** (-0.02 higher to 0.18 lower)	⊕◯◯◯ VERY LOW	weak
**Attention (post-test; assessed mainly with questionnaire and some using task)**												
15	RCTs	not double-blind	serious	not serious	not serious	none	1117	1025	-	SMD **0.16 SD higher** (0.01 lower to 0.32 lower)	⊕⊕◯◯ LOW	strong
**Depression (post-test; assessed with questionnaire)**												
14	RCTs	not double-blind	serious	not serious	serious	none	7548	7930	-	SMD **0.05 SD lower** (-0.02 higher to 0.12 lower)	⊕◯◯◯ VERY LOW	weak
**Executive Functions (post-test; assessed mainly with questionnaire and some using task)**												
23	RCTs	not double-blind	very serious	not serious	not serious	publication bias suspected	5296	5356	-	SMD **0.19 SD higher** (0.06 higher to 0.31 higher)	⊕◯◯◯ VERY LOW	weak
**Mindfulness (post-test; assessed with questionnaire)**												
14	RCTs	not double-blind	very serious	not serious	serious	publication bias suspected	5048	4996	-	SMD **0.10 SD higher** (-0.04 lower to 0.24 higher)	⊕◯◯◯ VERY LOW	weak
**Negative Behaviour (post-test; assessed mainly with questionnaire and a few observational)**												
11	RCTs	not double-blind	very serious	not serious	not serious	publication bias suspected potential dose response	4715	4718	-	SMD **0.31 SD lower** (0.10 lower to 0.51 lower)	⊕⊕◯◯ LOW	strong
**Social Behaviour (post-test; assessed with questionnaire)**												
11	RCTs	not double-blind	very serious	not serious	not serious	publication bias suspected	4785	4696	-	SMD **0.20 SD higher** (0.04 higher to 0.36 higher)	⊕◯◯◯ VERY LOW	weak
**Wellbeing (post-test; assessed with questionnaire)**												
9	RCTs	not double-blind	serious	not serious	serious	publication bias suspected	5787	6031	-	SMD **0.05 SD higher** (-0.04 lower to 0.13 higher)	⊕◯◯◯ VERY LOW	weak

RCTs: Randomized Controlled Trials. **CI:** Confidence interval; **SMD:** Standardized mean difference

Author(s): Dunning et al. 2022
Question: mindfulness for improving cognitive skills, behavior and mental health of children and adolescents at follow-up
Setting: Schools

Certainty assessment							№ of participants		Effect		Certainty	Importance
№ of studies	Study design	Risk of bias	Inconsistency	Indirectness	Imprecision	Other considerations	Mindfulness	TUE	Relative (95% CI)	Absolute (95% CI)		
**Anxiety/Stress (follow up: median 3 weeks; assessed with: Questionnaire)**												
10	RCTs	not double-blind	serious	not serious	serious	Potential dose response	4613	4807	-	SMD **0.05 SD lower** (-0.04 higher to 0.14 lower)	⊕⊕◯◯ LOW	weak
**Attention (follow up: median 3 weeks; assessed with: ; assessed mainly with questionnaire and some using task)**												
5	RCTs	not double-blind	very serious	not serious	serious	none	85	69	-	SMD **0.72 SD higher** (-0.22 lower to 1.66 higher)	⊕◯◯◯ VERY LOW	weak
**Depression (follow up: median 3 weeks; assessed with: Questionnaire)**												
12	RCTs	not double-blind	serious	not serious	serious	none	6826	7781	-	SMD **0.01 SD lower** (-0.04 higher to 0.07 lower)	⊕◯◯◯ VERY LOW	weak
**Executive Functions (follow up: median 3 weeks; assessed mainly with questionnaire and some using task)**												
9	RCTs	not double-blind	very serious	not serious	serious	none	4055	4480	-	SMD **0.27 SD higher** (-0.03 lower to 0.57 higher)	⊕◯◯◯ VERY LOW	weak
**Mindfulness (follow up: median 3 weeks; assessed with: Questionnaire)**												
13	RCTs	not double-blind	serious	not serious	serious	none	4714	4945	-	SMD **0.01 SD higher** (-0.10 lower to 0.13 higher)	⊕◯◯◯ VERY LOW	weak
**Negative Behaviour (follow up: median 3.5 weeks; assessed with: Questionnaire)**												
6	RCTs	not double-blind	serious	not serious	serious	Potential dose response	4021	4375	-	SMD **0.10 SD lower** (-0.12 higher to 0.32 lower)	⊕⊕◯◯ LOW	weak
**Social Behaviour (follow up: median 3 weeks; assessed with: Questionnaire)**												
7	RCTs	not double-blind	serious	not serious	not serious	none	4293	4616	-	SMD **0.12 SD higher** (0.002 higher to 0.24 higher)	⊕⊕◯◯ LOW	strong
**Wellbeing (follow up: median 3 weeks; assessed with: Questionnaire)**												
9	RCTs	not double-blind	not serious	not serious	serious	none	5735	6332	-	SMD **0.00 SD higher** (-0.04 lower to 0.03 higher)	⊕⊕◯◯ LOW	weak

RCTs: Randomized Controlled Trials. **CI:** Confidence interval; **SMD:** Standardized mean difference

Supplement G

Forest plots for all MBPs, MBP vs active controls; MBP vs passive controls; MBPs as Selective interventions; MWP as Universal interventions

Note: positive effect sizes show MBPs superior to controls; larger plot size of squares represent larger proportional weight of each study. The lozenge at the bottom of each forest plots represents the grand mean

1. <u>All studies</u>

1.1 Forest plot showing standardised difference in mean and 95% confidence intervals for anxiety/stress outcomes following a MBP

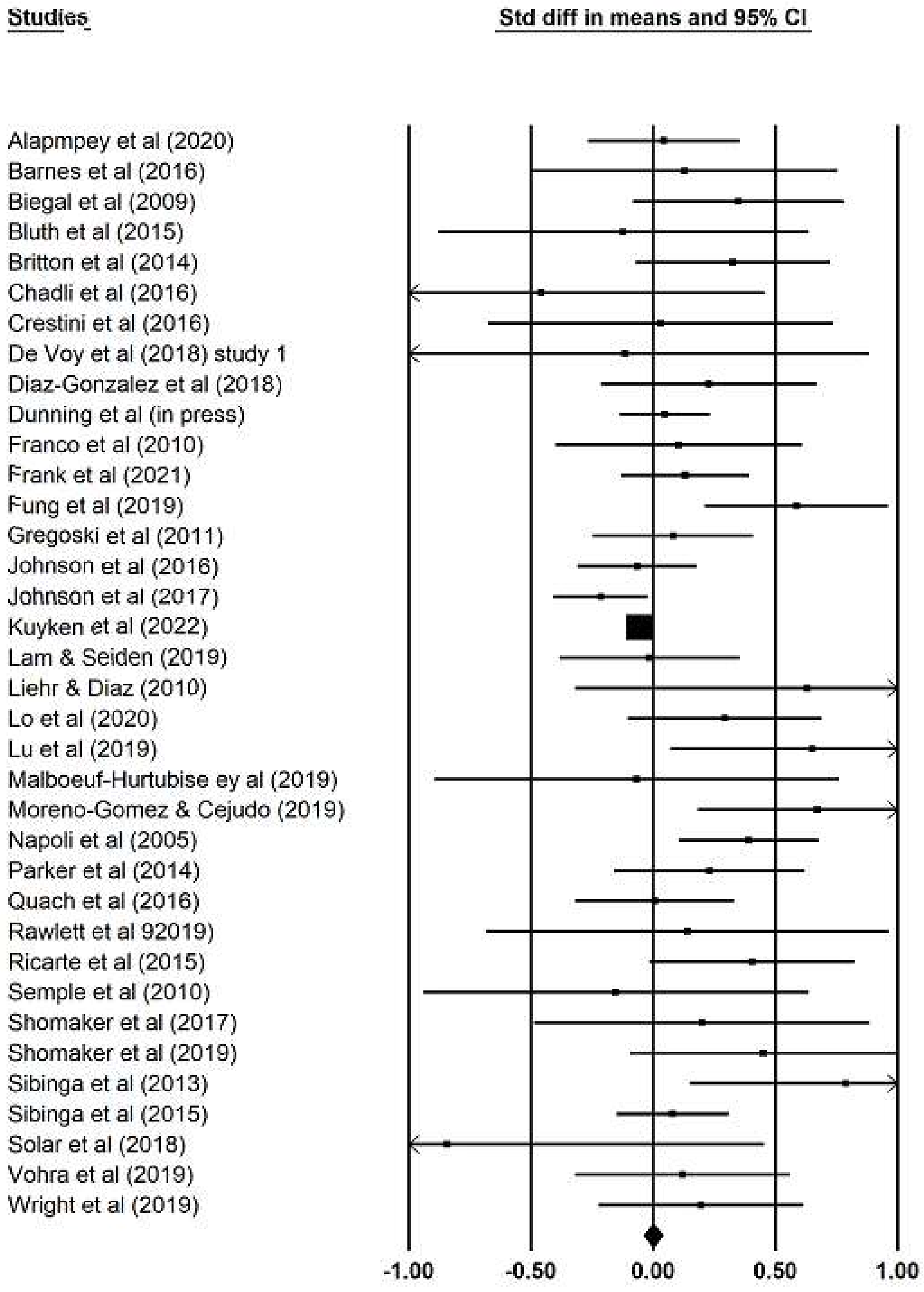

1.2 Forest plot showing standardised difference in mean and 95% confidence intervals for attention outcomes following mindfulness training

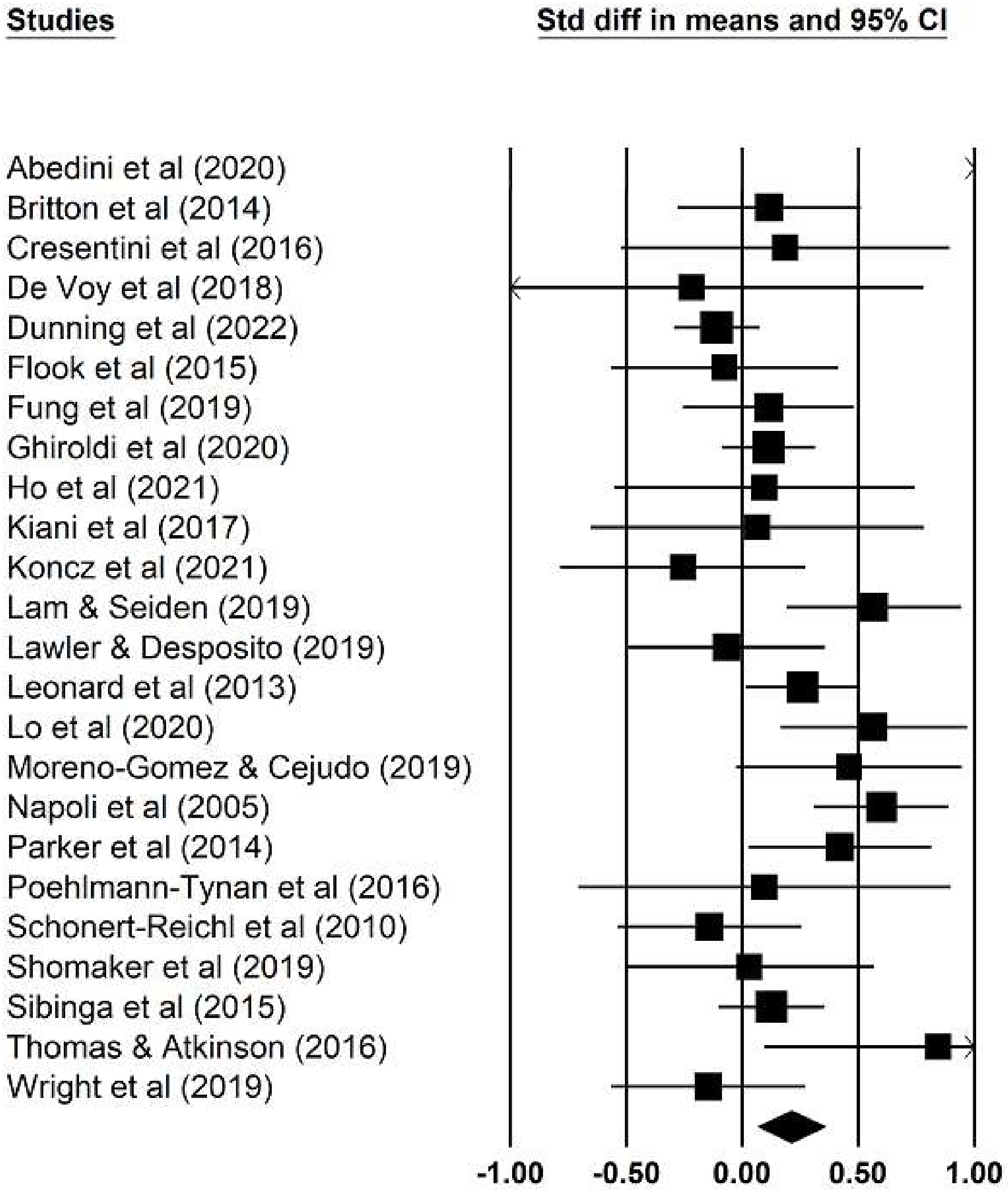

Dunning D, *et al. Evid Based Ment Health* 2022; 25:135–142. doi: 10.1136/ebmental-2022-300464

1.3 Forest plot showing standardised difference in mean and 95% confidence intervals for depression outcomes following mindfulness training

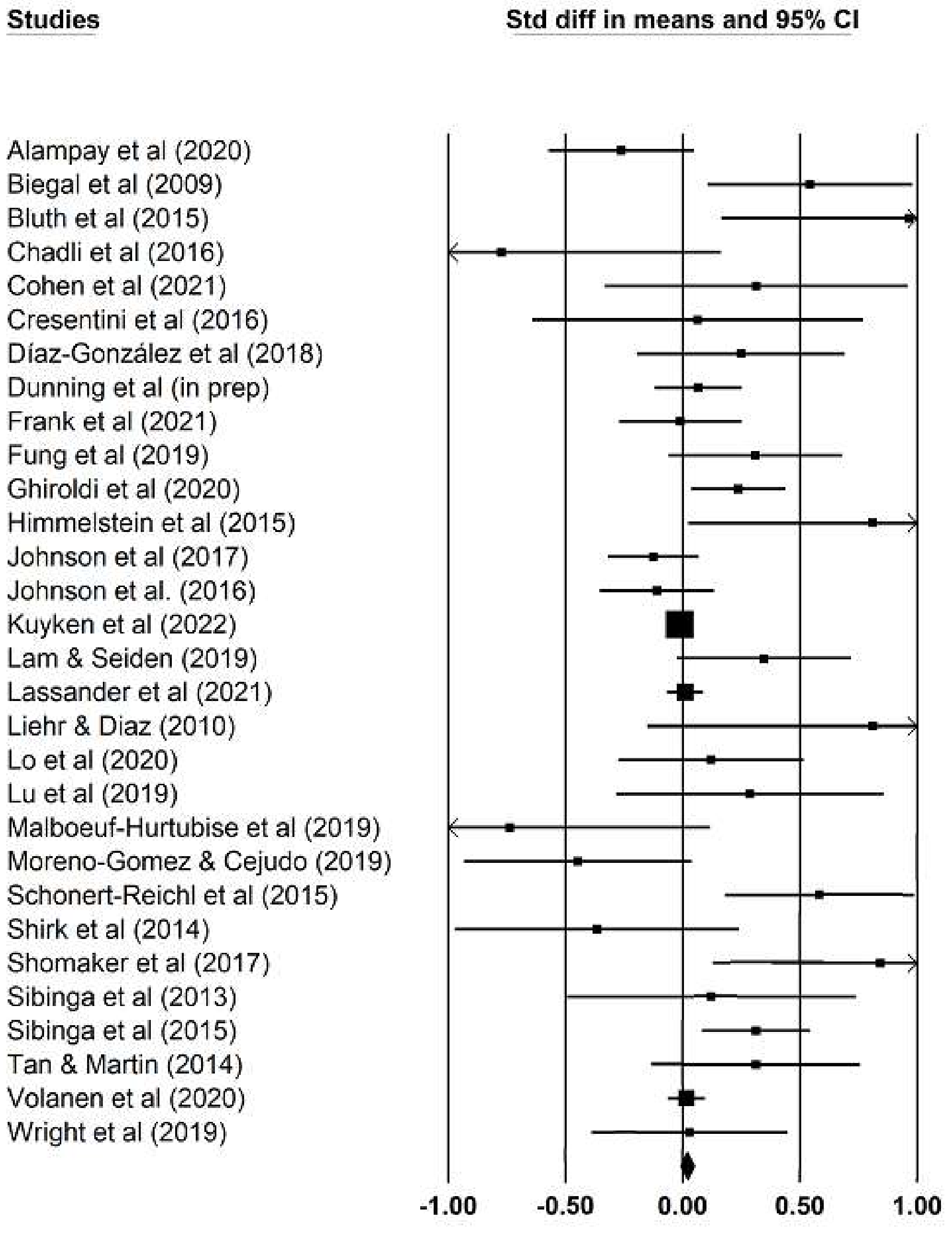

Dunning D, *et al. Evid Based Ment Health* 2022; 25:135–142. doi: 10.1136/ebmental-2022-300464

1.4 Forest plot showing standardised difference in mean and 95% confidence intervals for executive functions outcomes following mindfulness training

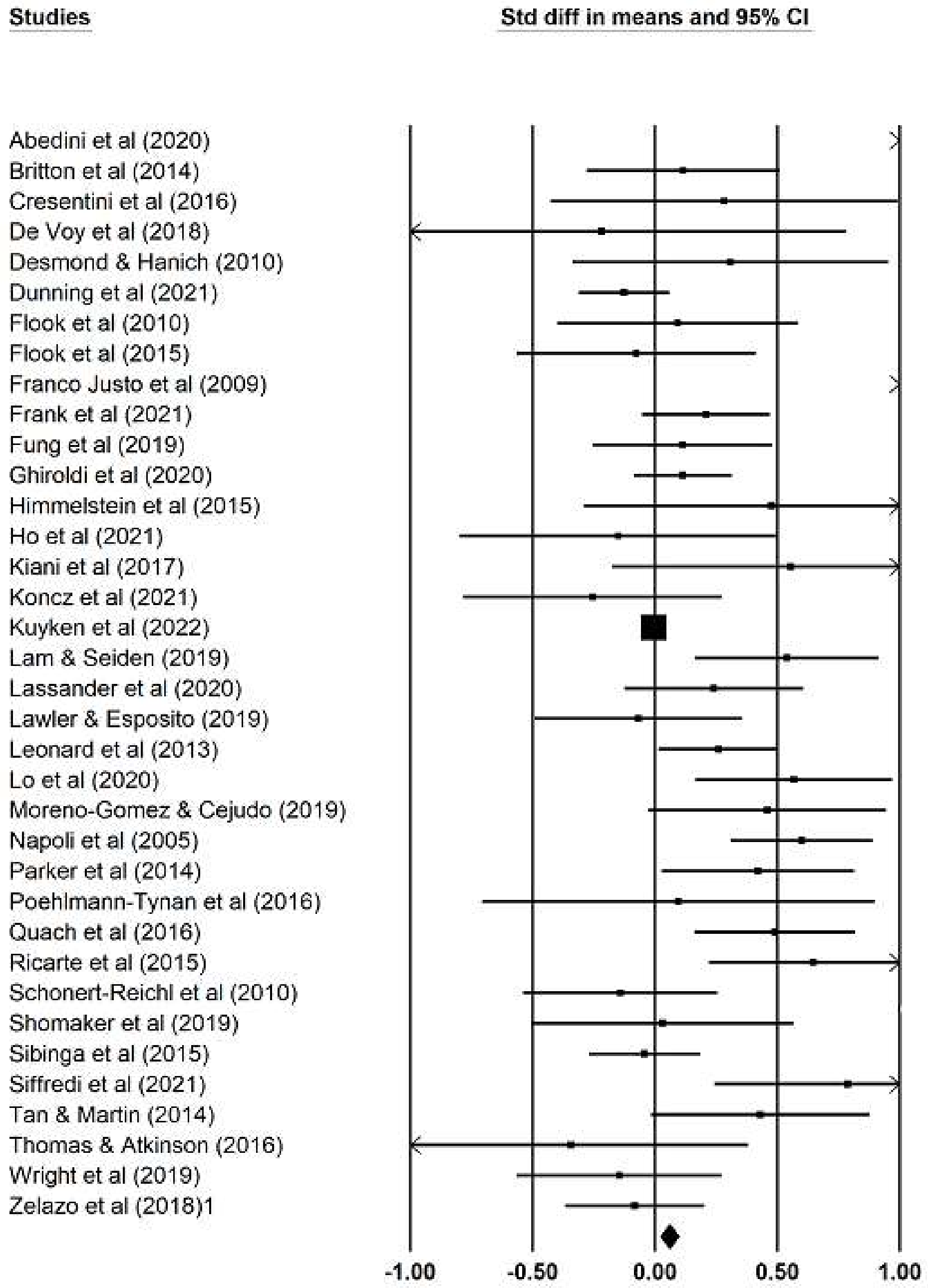

Dunning D, *et al. Evid Based Ment Health* 2022; 25:135–142. doi: 10.1136/ebmental-2022-300464

1.5 *Forest plot showing standardised difference in mean and 95% confidence intervals for mindfulness outcomes following mindfulness training*

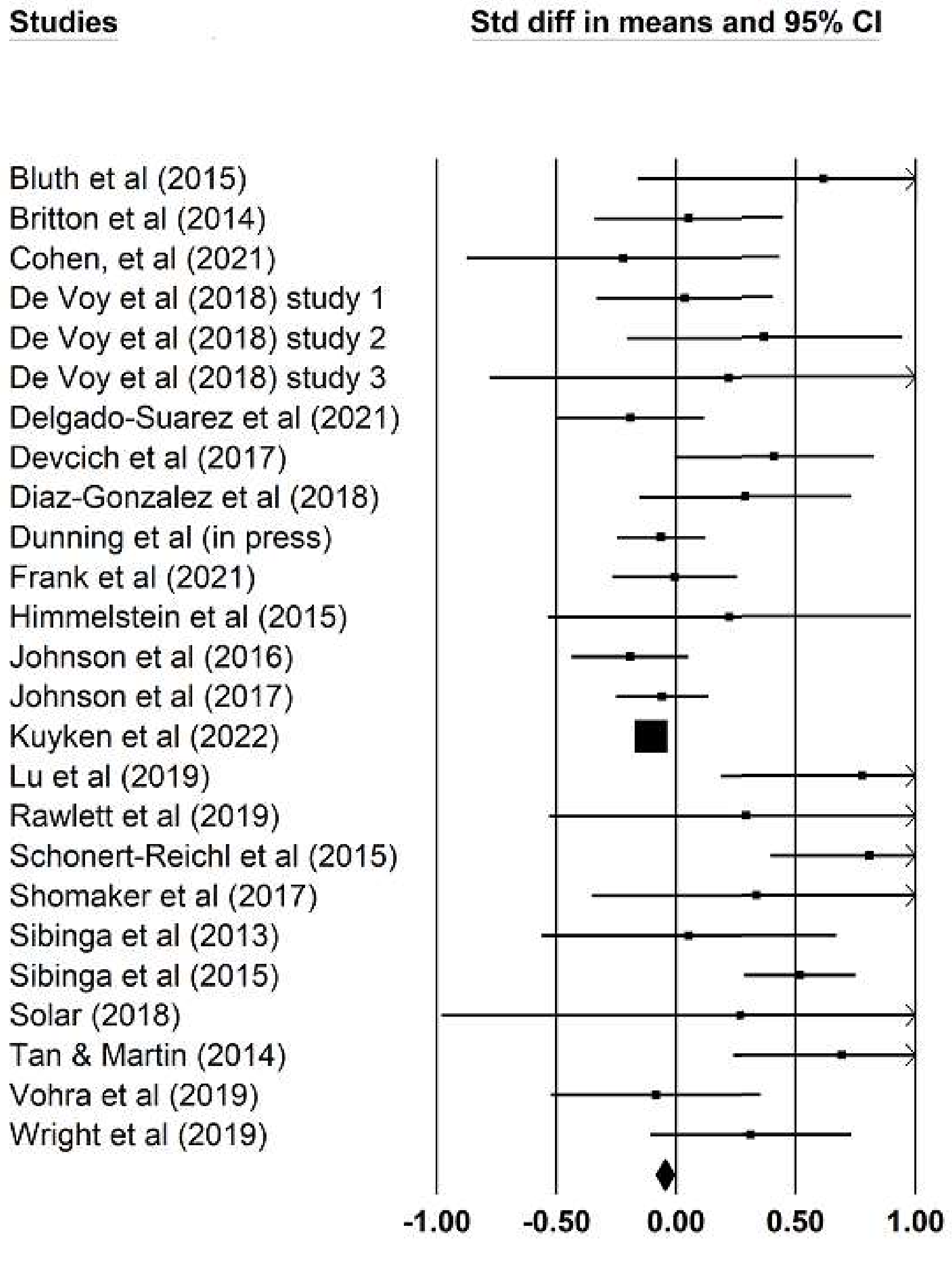

1.6 Forest plot showing standardised difference in mean and 95% confidence intervals for negative behaviour outcomes following mindfulness training

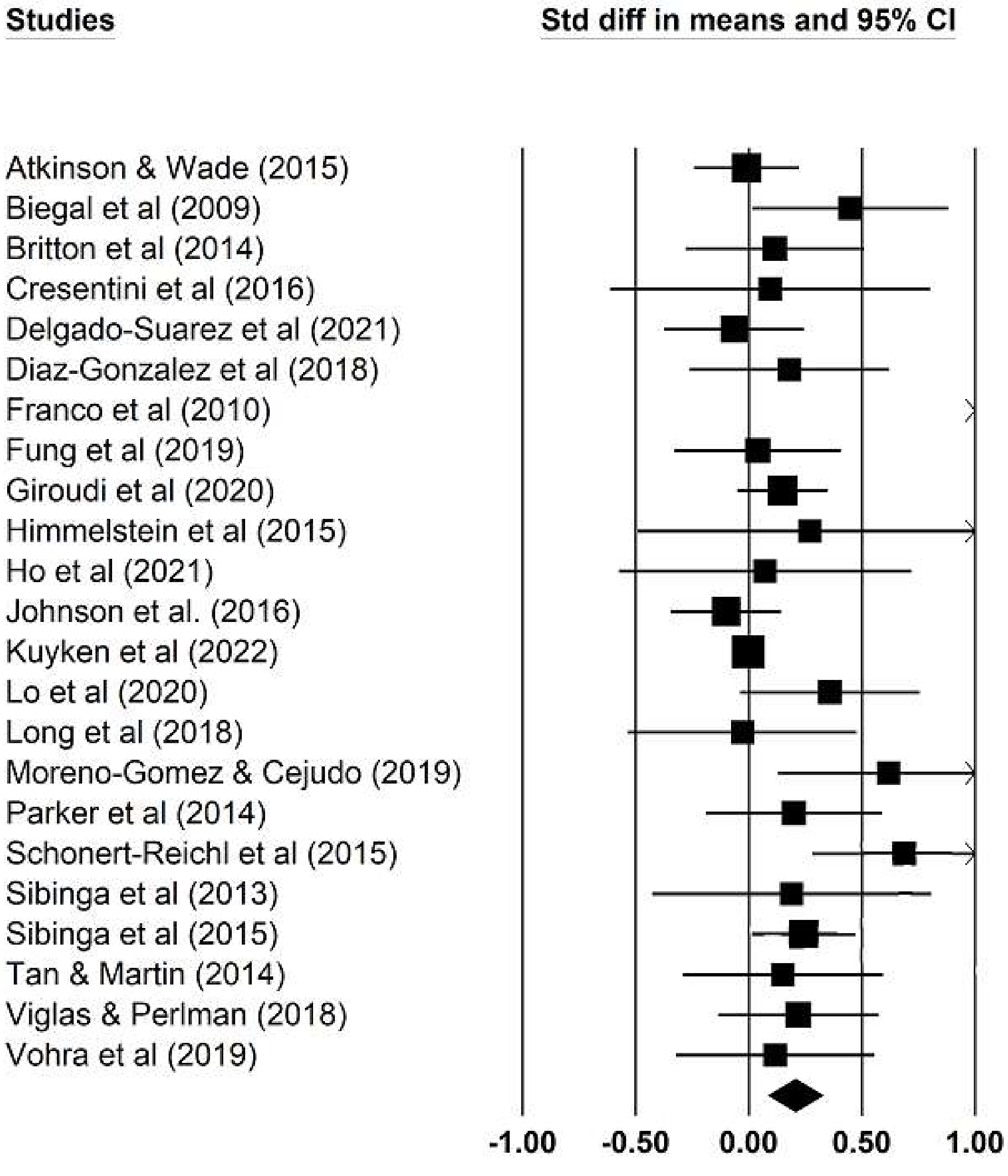

1.7 Forest plot showing standardised difference in mean and 95% confidence intervals for social behaviour outcomes following mindfulness training

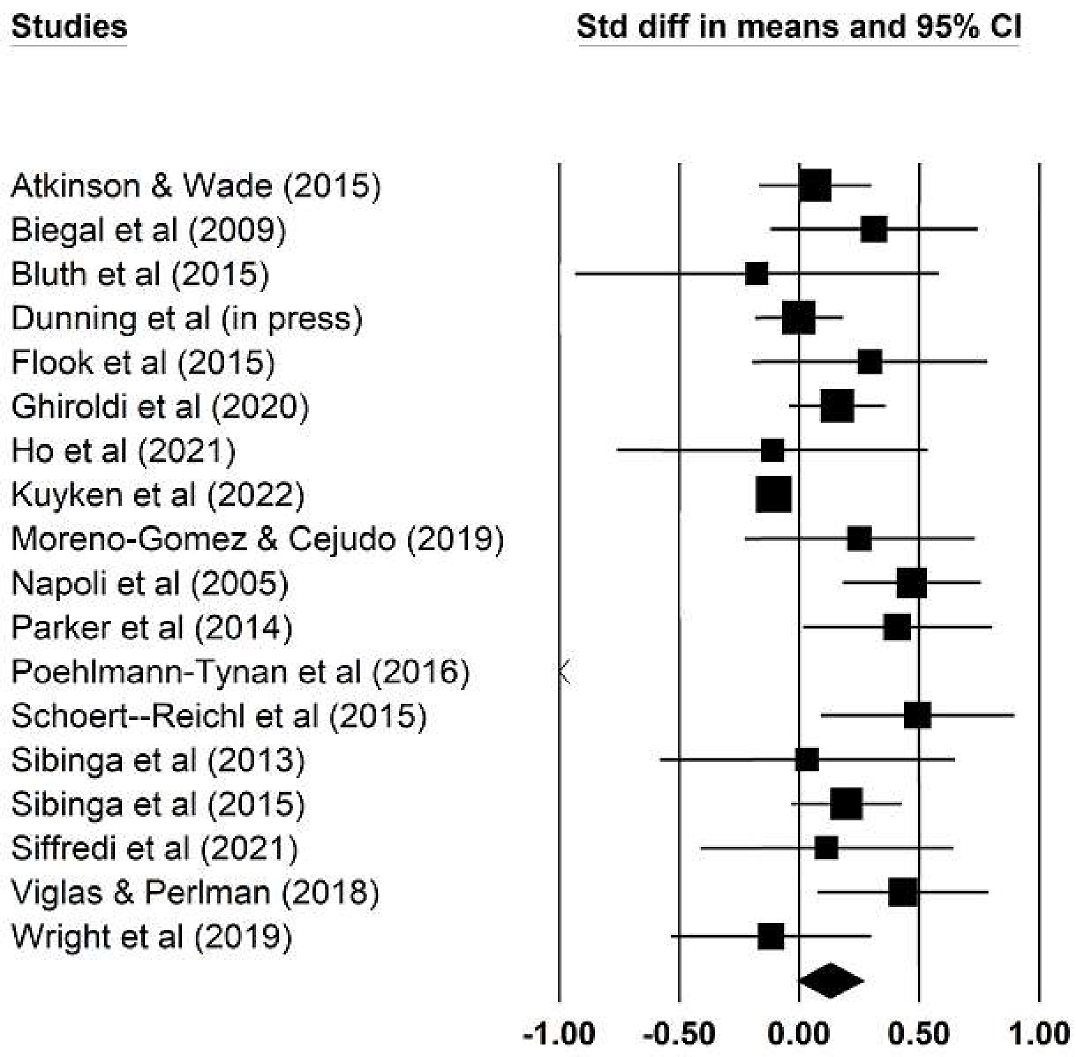

1.8 Forest plot showing standardised difference in mean and 95% confidence intervals for wellbeing outcomes following mindfulness training

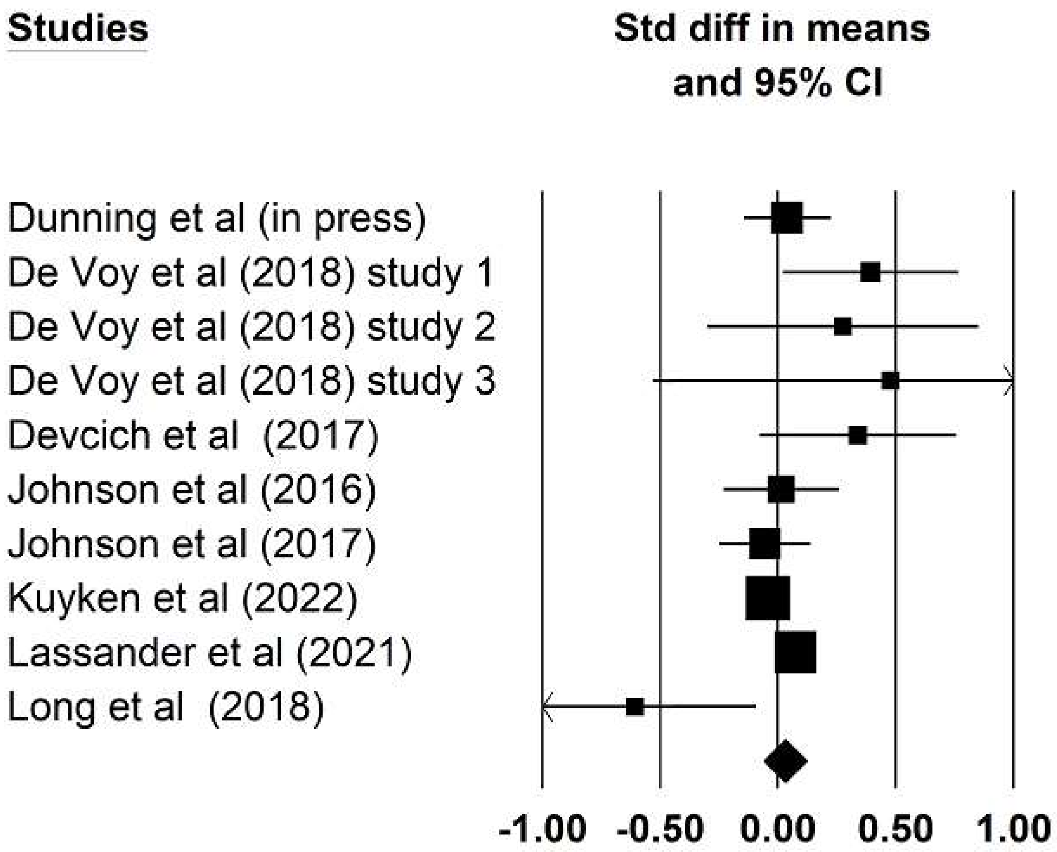

2. Mindfulness compared to Active controls

2.1 *Forest plot showing standardised difference in mean and 95% confidence intervals for anxiety/stress outcomes following mindfulness training compared to active control groups*

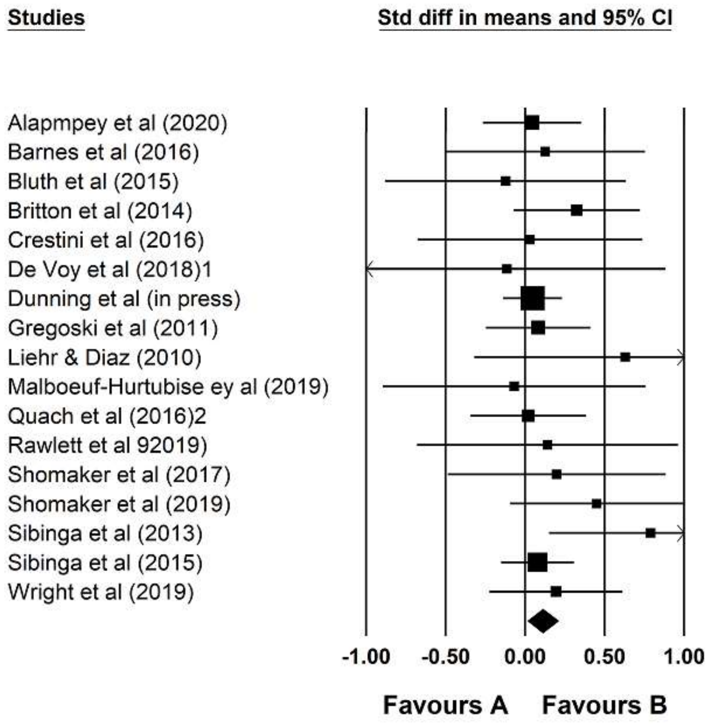

2.2 *Forest plot showing standardised difference in mean and 95% confidence intervals for attention outcomes following mindfulness training compared to active control groups*

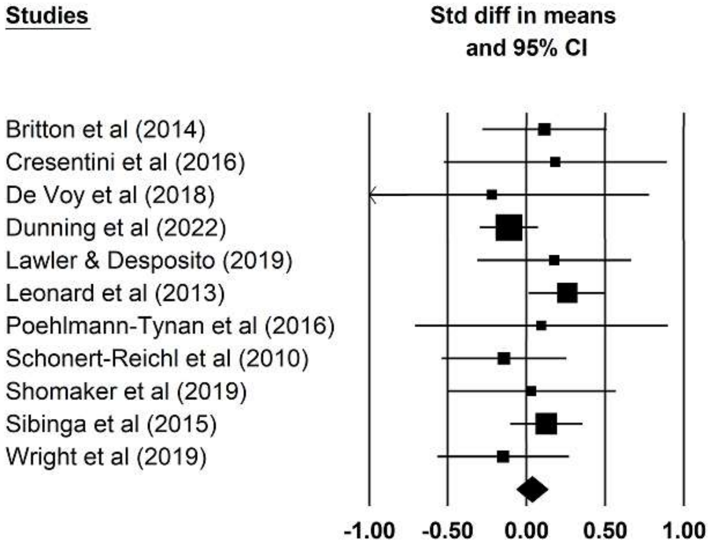

Dunning D, *et al. Evid Based Ment Health* 2022; 25:135–142. doi: 10.1136/ebmental-2022-300464

2.3 Forest plot showing standardised difference in mean and 95% confidence intervals for depression outcomes following mindfulness training compared to active control groups

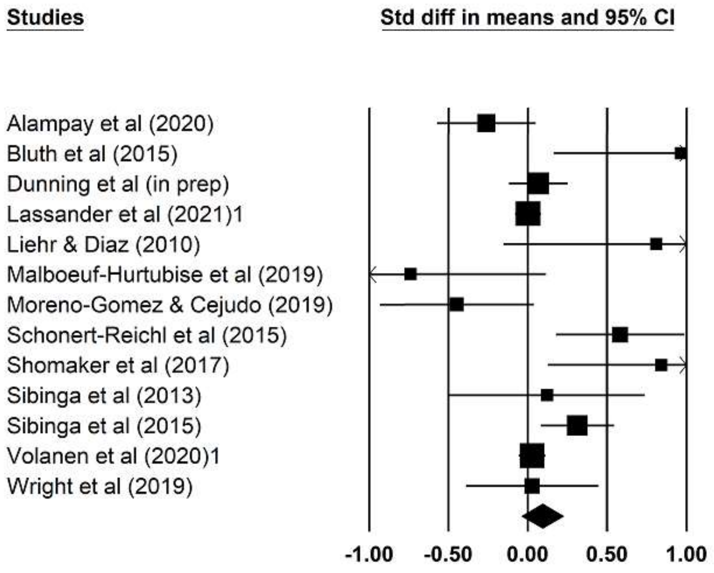

2.4 *Forest plot showing standardised difference in mean and 95% confidence intervals for executive function outcomes following mindfulness training compared to active control groups*

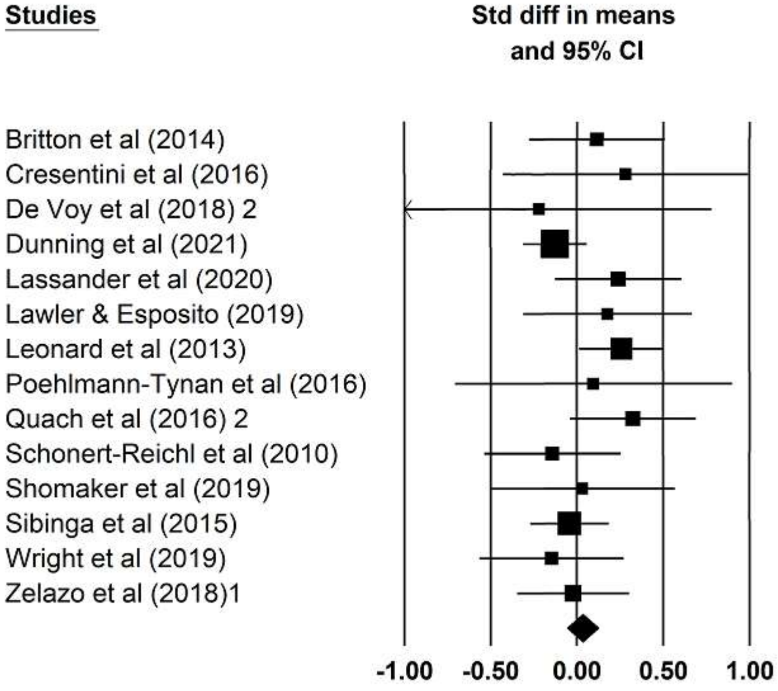

2.5 Forest plot showing standardised difference in mean and 95% confidence intervals for mindfulness outcomes following mindfulness training compared to active control groups

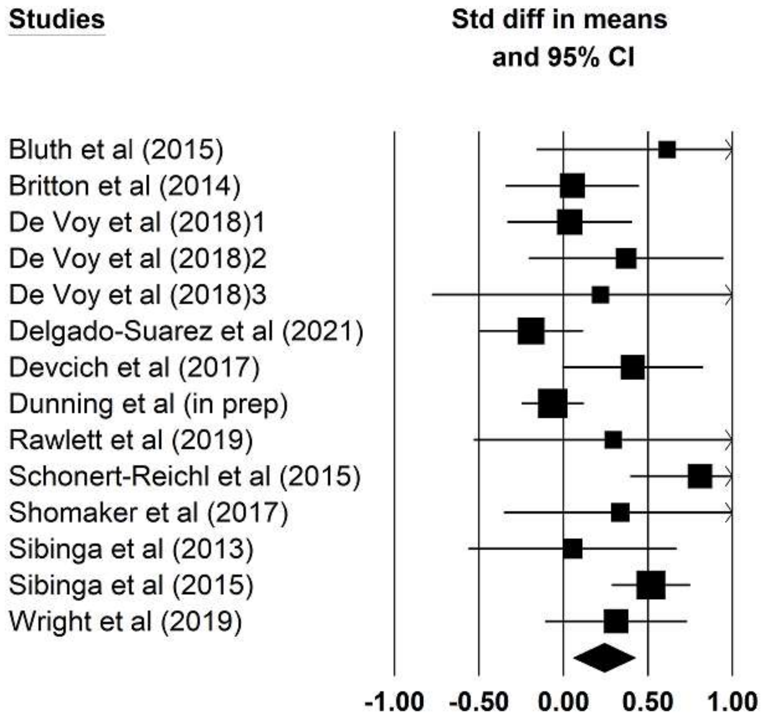

2.6 Forest plot showing standardised difference in mean and 95% confidence intervals for negative behaviour outcomes following mindfulness training compared to active control groups

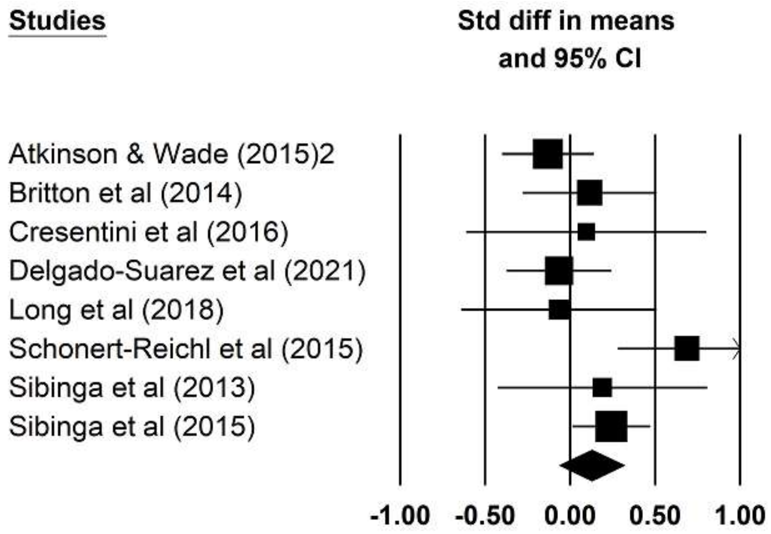

2.7 Forest plot showing standardised difference in mean and 95% confidence intervals for social behaviour outcomes following mindfulness training compared to active control groups

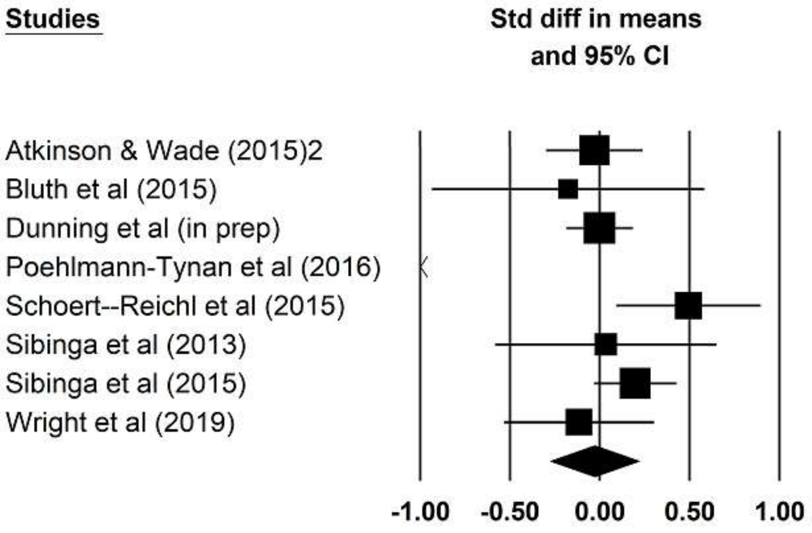

2.8 Forest plot showing standardised difference in mean and 95% confidence intervals for wellbeing outcomes following mindfulness training compared to active control groups

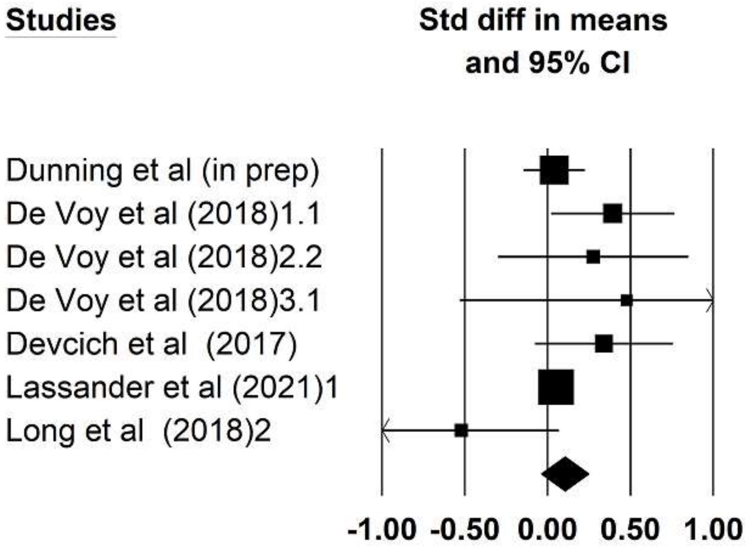

Dunning D, *et al. Evid Based Ment Health* 2022; 25:135–142. doi: 10.1136/ebmental-2022-300464

3. Mindfulness versus passive groups

3.1 Forest plot showing standardised difference in mean and 95% confidence intervals for wellbeing outcomes following mindfulness training compared to passive control groups

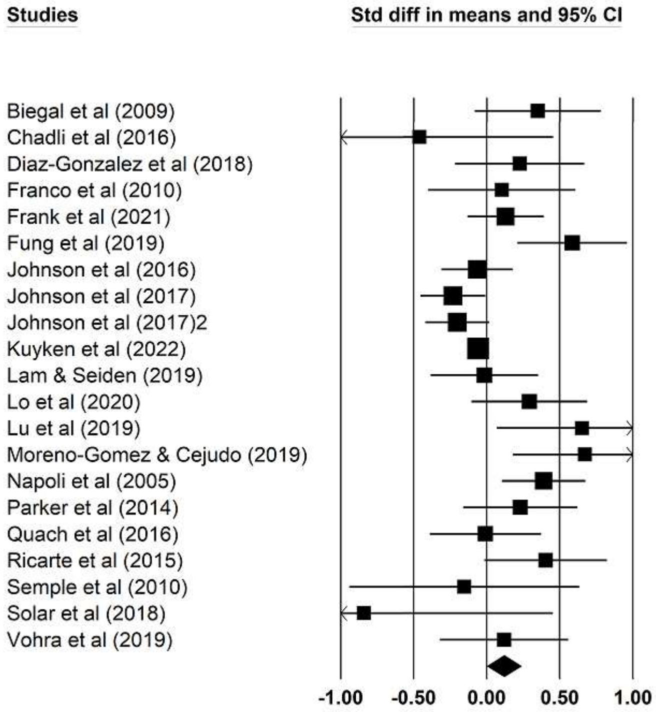

3.2 Forest plot showing standardised difference in mean and 95% confidence intervals for attention outcomes following mindfulness training compared to passive control groups

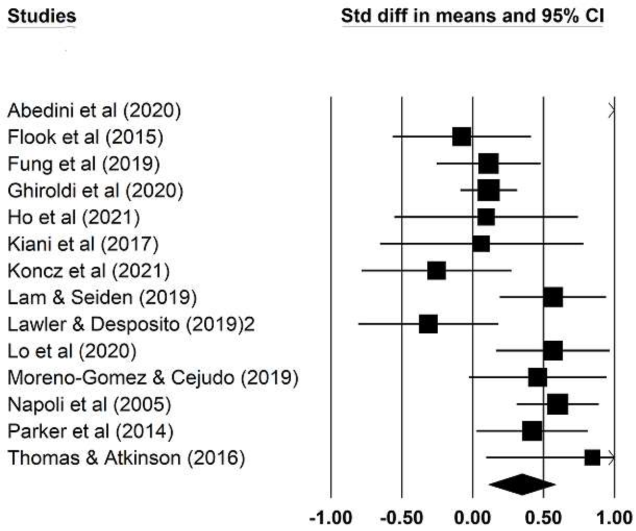

3.3 Forest plot showing standardised difference in mean and 95% confidence intervals for depression outcomes following mindfulness training compared to passive control groups

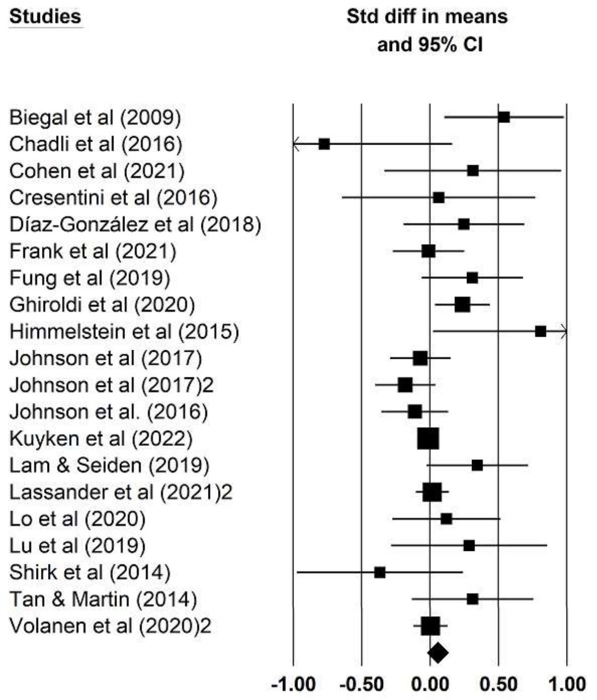

3.4 *Forest plot showing standardised difference in mean and 95% confidence intervals for executive function outcomes following mindfulness training compared to passive control groups*

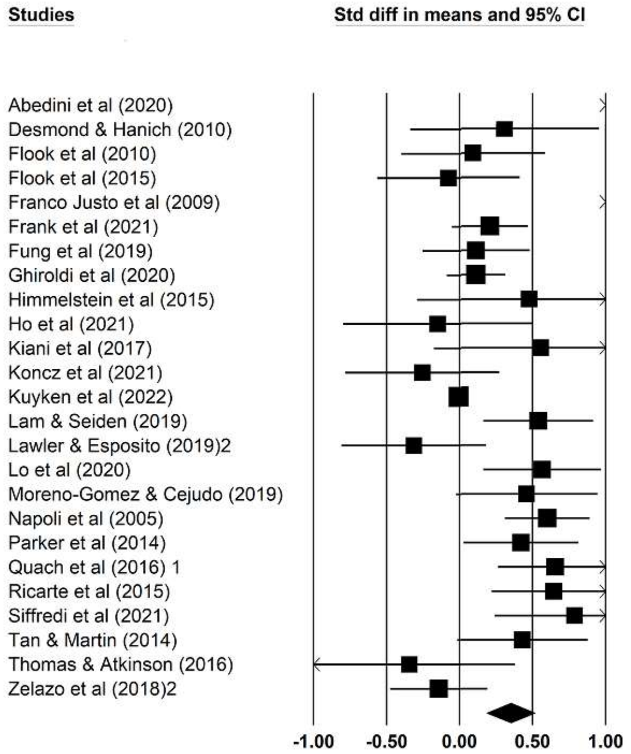

3.5 Forest plot showing standardised difference in mean and 95% confidence intervals for mindfulness outcomes following mindfulness training compared to passive control groups

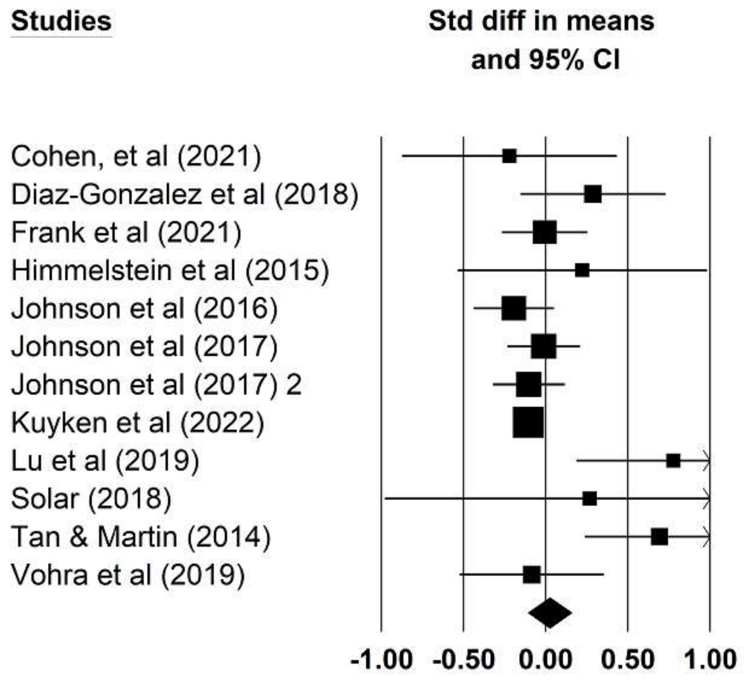

3.*6 Forest plot showing standardised difference in mean and 95% confidence intervals for negative behaviour outcomes following mindfulness training compared to passive control groups*

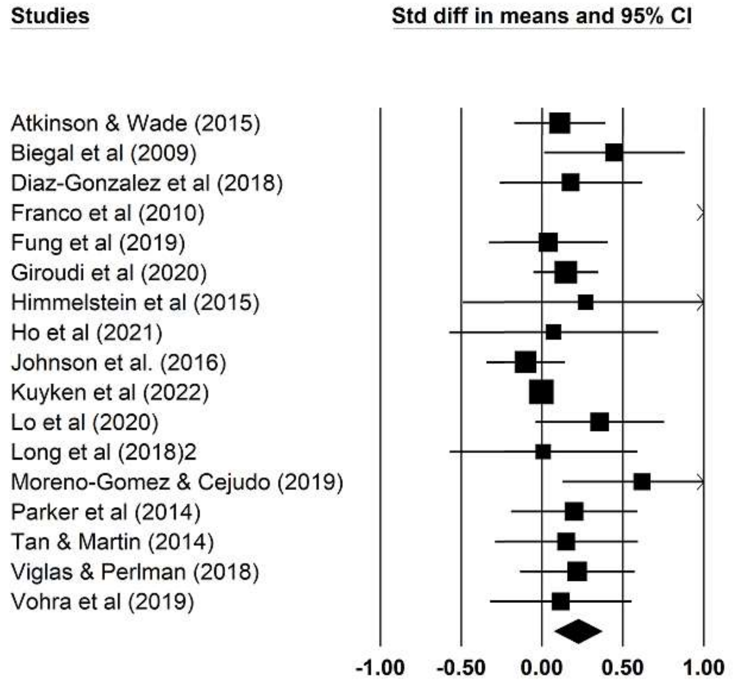

Dunning D, *et al. Evid Based Ment Health* 2022; 25:135–142. doi: 10.1136/ebmental-2022-300464

3.7 Forest plot showing standardised difference in mean and 95% confidence intervals for social behaviour outcomes following mindfulness training compared to passive control groups

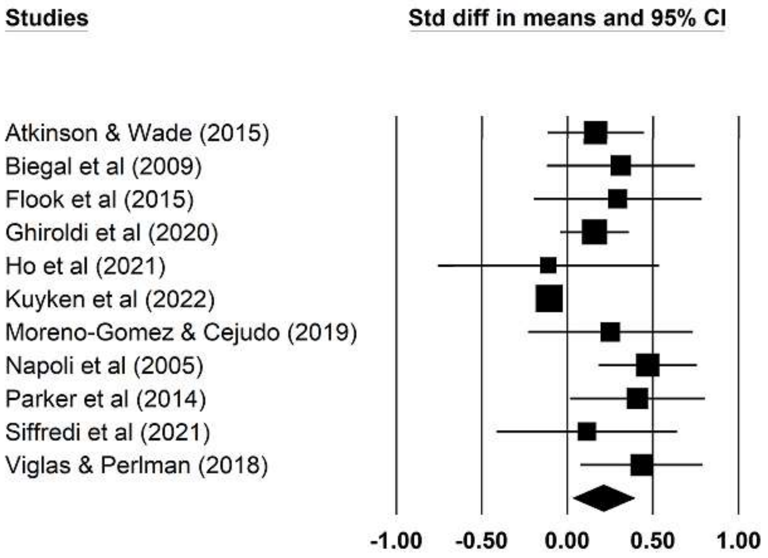

3.8 Forest plot showing standardised difference in mean and 95% confidence intervals for wellbeing outcomes following mindfulness training compared to passive control groups

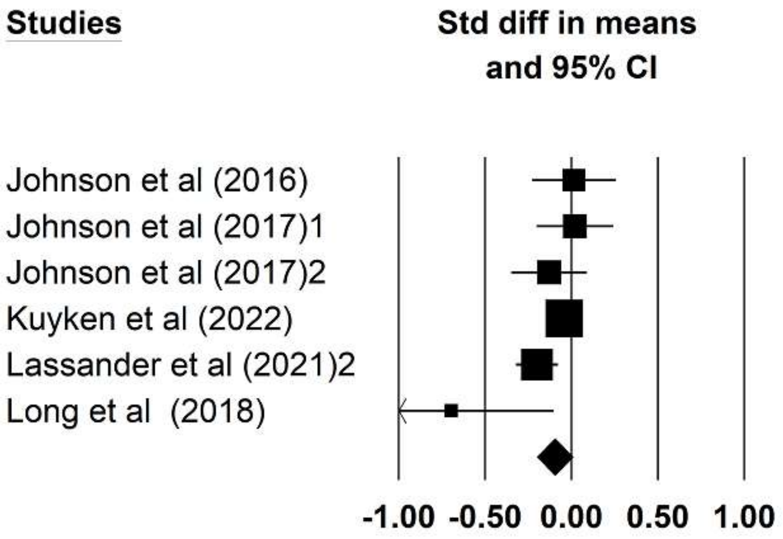

4. Mindfulness as a selective intervention

4.1 Forest plot showing standardised difference in mean and 95% confidence intervals for anxiety outcomes following mindfulness training as a selective intervention

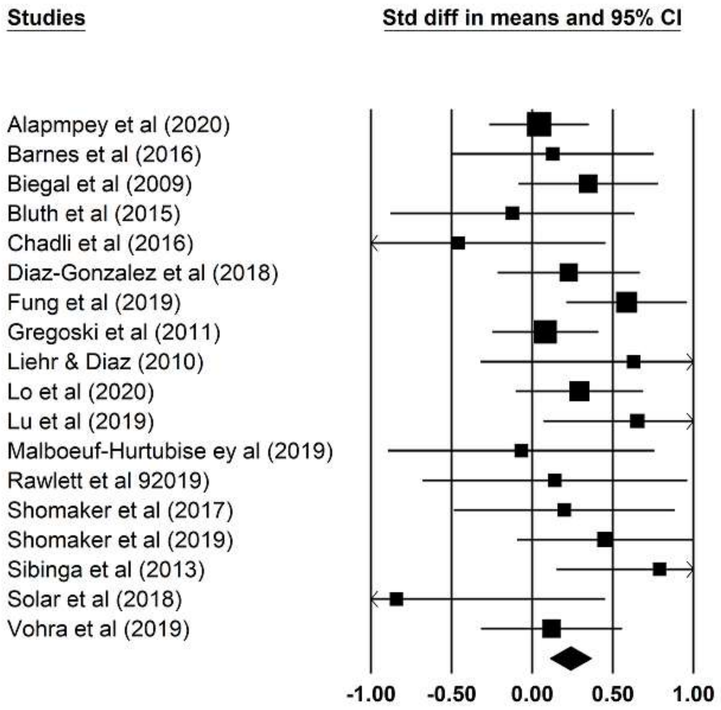

4.2 Forest plot showing standardised difference in mean and 95% confidence intervals for attention outcomes following mindfulness training as a selective intervention

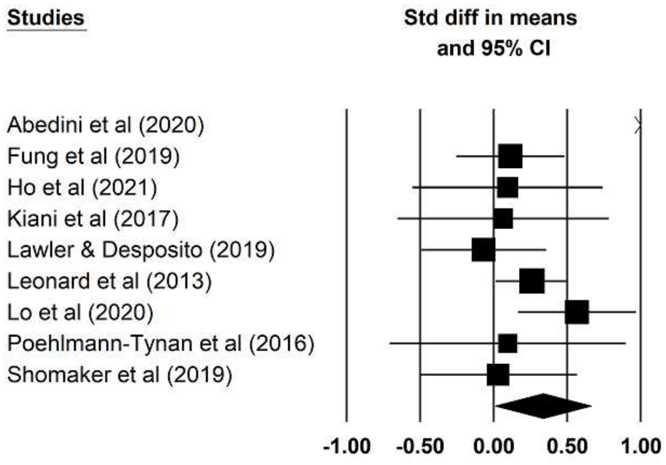

Dunning D, *et al. Evid Based Ment Health* 2022; 25:135–142. doi: 10.1136/ebmental-2022-300464

4.3 Forest plot showing standardised difference in mean and 95% confidence intervals for depression outcomes following mindfulness training as a selective intervention

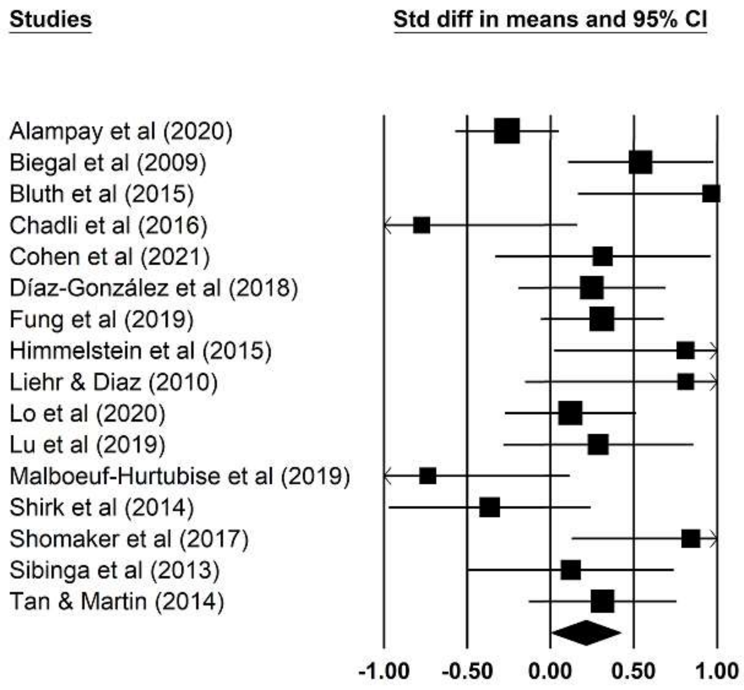

4.4 Forest plot showing standardised difference in mean and 95% confidence intervals for executive function outcomes following mindfulness training as a selective intervention

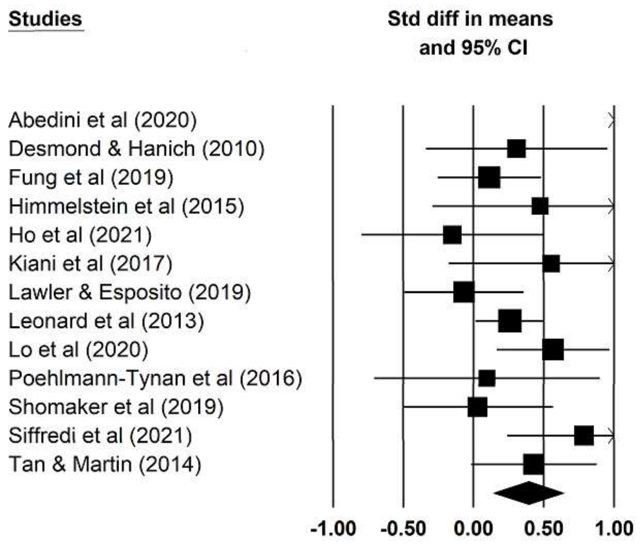

Dunning D, *et al. Evid Based Ment Health* 2022; 25:135–142. doi: 10.1136/ebmental-2022-300464

4.5 Forest plot showing standardised difference in mean and 95% confidence intervals for mindfulness outcomes following mindfulness training as a selective intervention

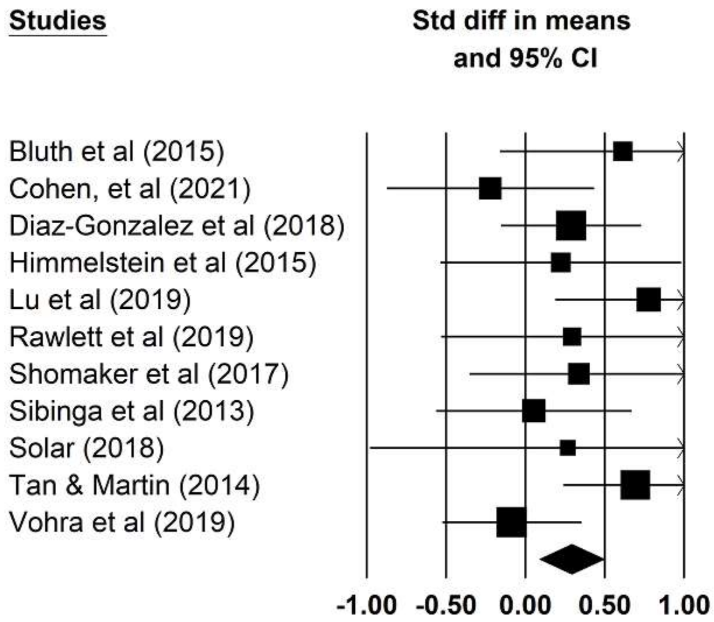

4.6 Forest plot showing standardised difference in mean and 95% confidence intervals for negative behaviour outcomes following mindfulness training as a selective intervention

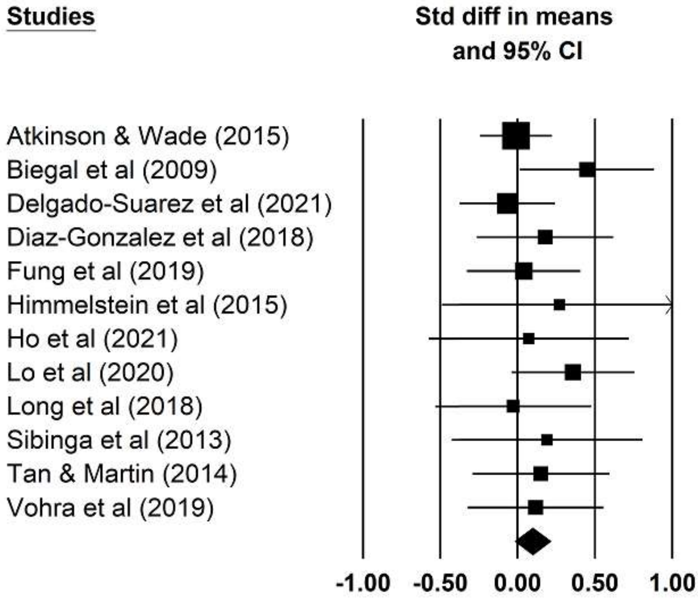

4.7 Forest plot showing standardised difference in mean and 95% confidence intervals for social behaviour outcomes following mindfulness training as a selective intervention

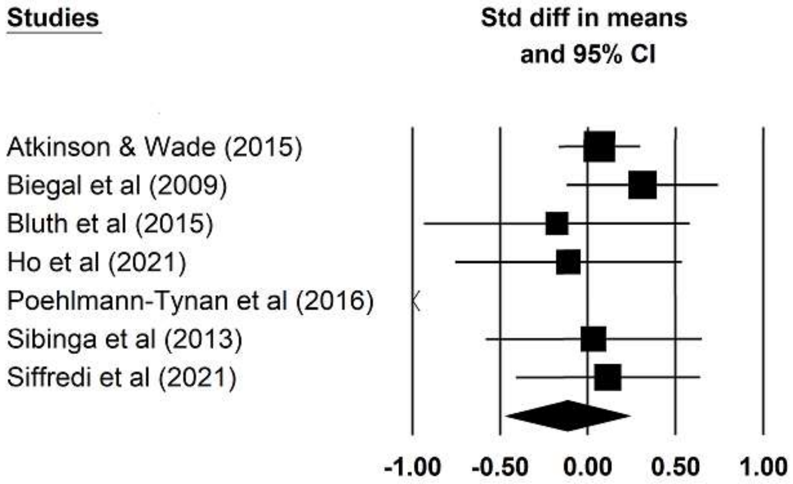

Dunning D, *et al. Evid Based Ment Health* 2022; 25:135–142. doi: 10.1136/ebmental-2022-300464

5. <u>Mindfulness as a universal intervention</u>

5.1 Forest plot showing standardised difference in mean and 95% confidence intervals for anxiety/stress outcomes following mindfulness training as a universal intervention

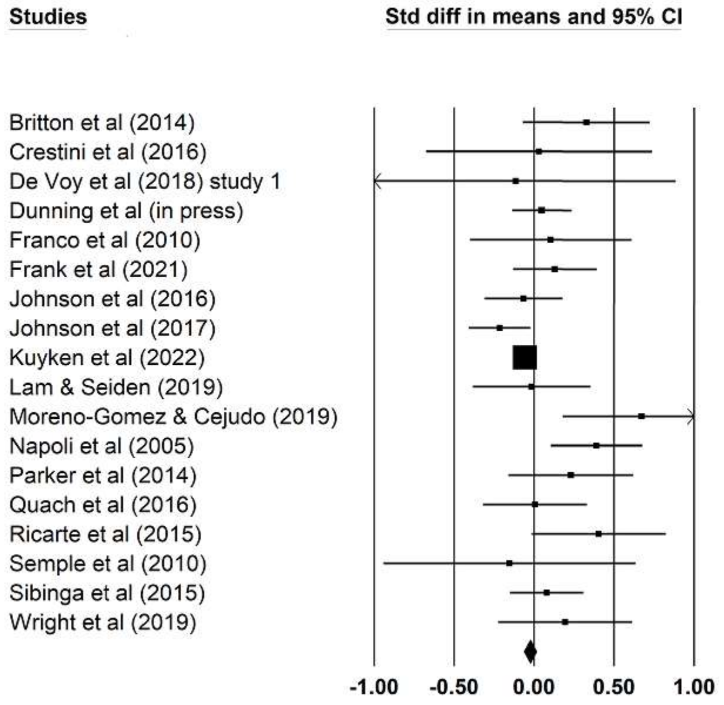

5.2 Forest plot showing standardised difference in mean and 95% confidence intervals for attention outcomes following mindfulness training as a universal intervention

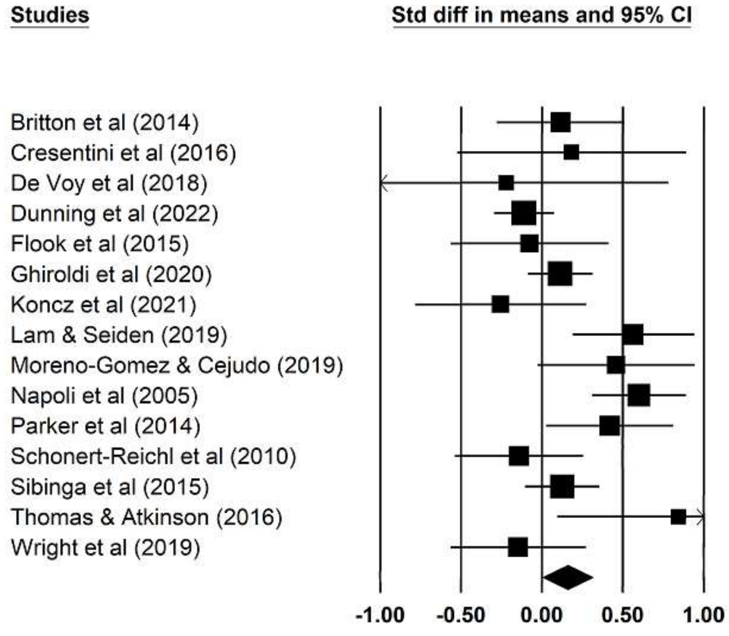

5.3 Forest plot showing standardised difference in mean and 95% confidence intervals for depression outcomes following mindfulness training as a universal intervention

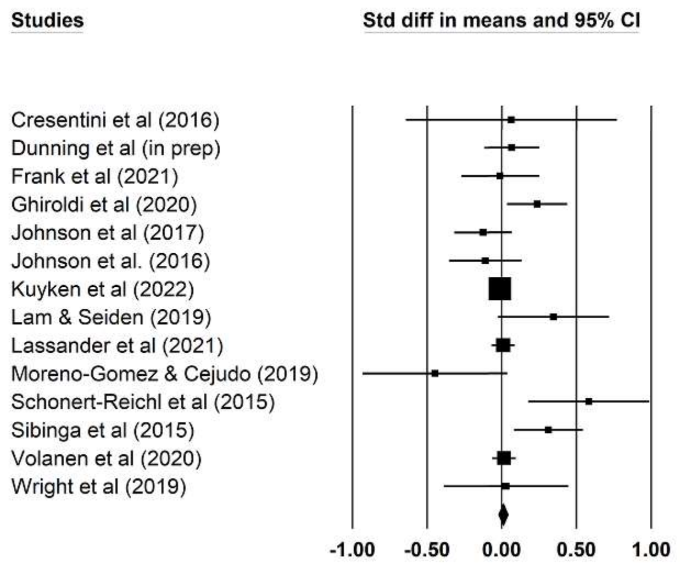

5.4 Forest plot showing standardised difference in mean and 95% confidence intervals executive functioning outcomes following mindfulness training as a universal intervention

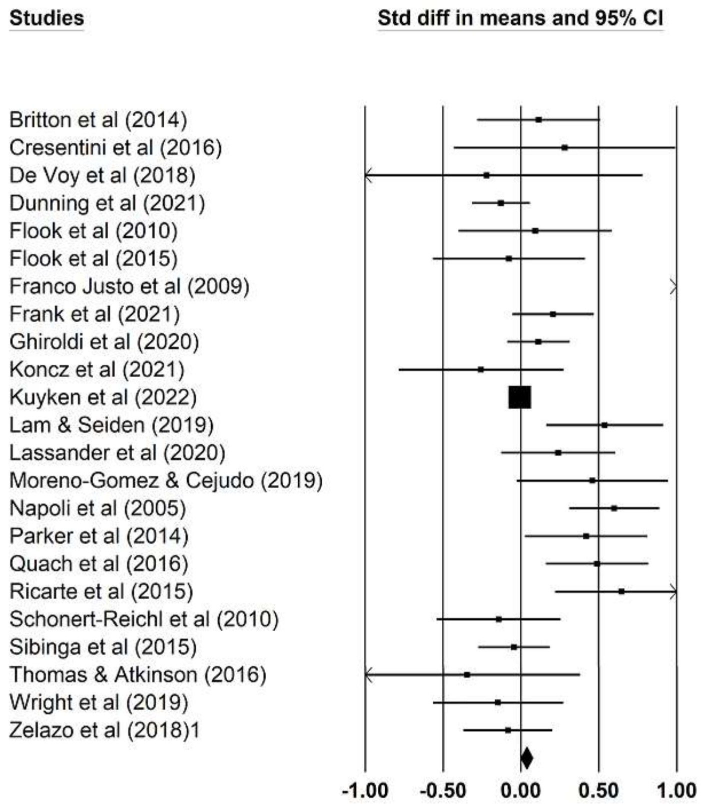

Dunning D, *et al. Evid Based Ment Health* 2022; 25:135–142. doi: 10.1136/ebmental-2022-300464

5.5 Forest plot showing standardised difference in mean and 95% confidence intervals for mindfulness outcomes following mindfulness training as a universal intervention

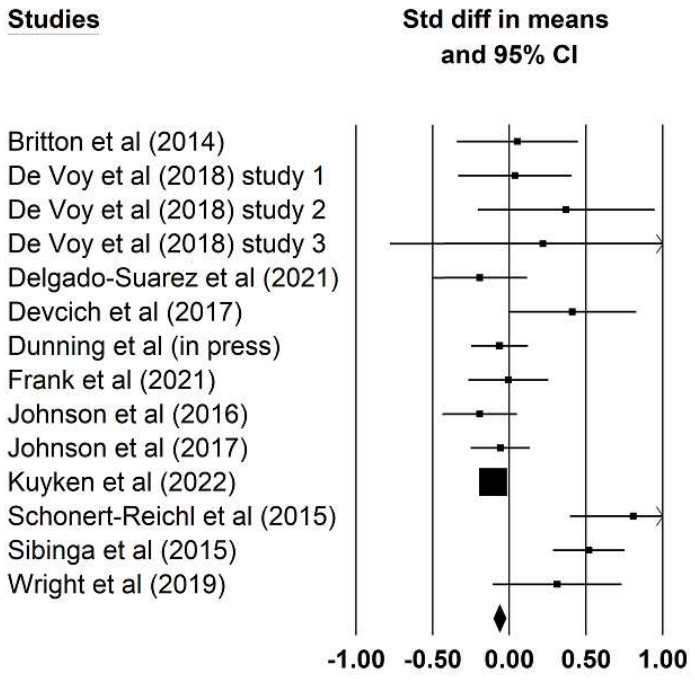

5.6 Forest plot showing standardised difference in mean and 95% confidence intervals for negative behaviour outcomes following mindfulness training as a universal intervention

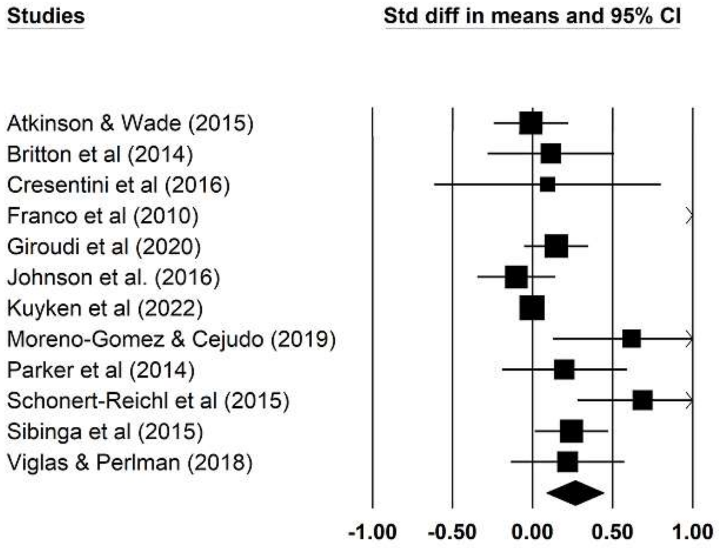

5.7 Forest plot showing standardised difference in mean and 95% confidence intervals for social behaviour outcomes following mindfulness training as a universal intervention

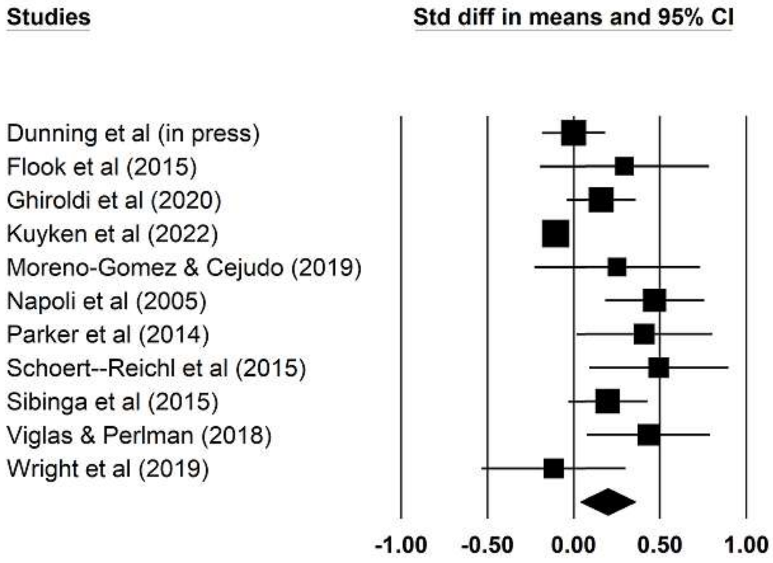

5.8 Forest plot showing standardised difference in mean and 95% confidence intervals for wellbeing outcomes following mindfulness training as a universal intervention

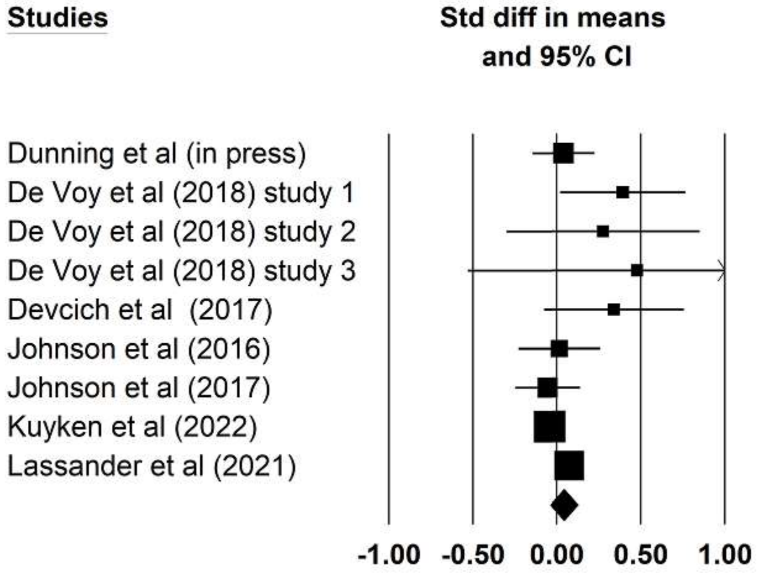

Dunning D, *et al. Evid Based Ment Health* 2022; 25:135–142. doi: 10.1136/ebmental-2022-300464

Supplement H

Funnel plots examining publication bias. Funnel plots for all MBPs, MBP vs active controls; MBP vs passive controls; MBPs as Selective interventions; MWP as Universal interventions

NB: The funnel it based on the assumption that studies with high precision will be plotted near the average, and studies with low precision will be spread evenly on both sides of the average, creating a roughly funnel-shaped distribution. All funnel plots show the observed standardized difference in means and standard error in transparent circles and imputed/missing studies in black. Black dots were generated using the Trim and Fill method (Duvall & Tweedie, 2000). The transparent lozenge at the bottom of the forest plot shows the mean for all studies. The black lozenge at the bottom of the forest plot shows the mean for all studies, including imputed/missing studies. The horizontal points of the diamond are 95% confidence intervals.

Dunning D, *et al. Evid Based Ment Health* 2022; 25:135–142. doi: 10.1136/ebmental-2022-300464

1. All studies

1.1 Funnel plot for anxiety/stress outcomes following Mindfulness training

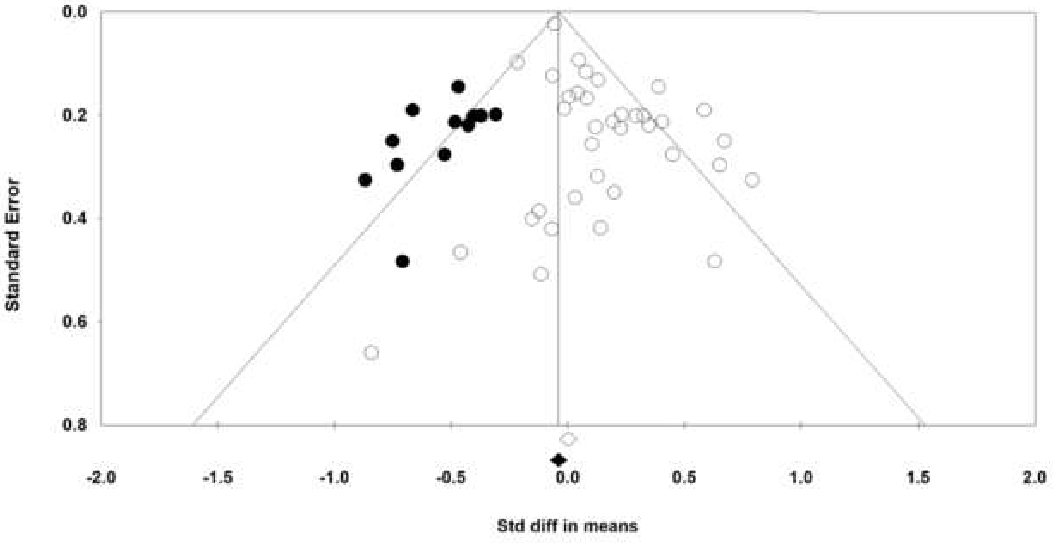

1.2 Funnel plot for attention outcomes following Mindfulness training

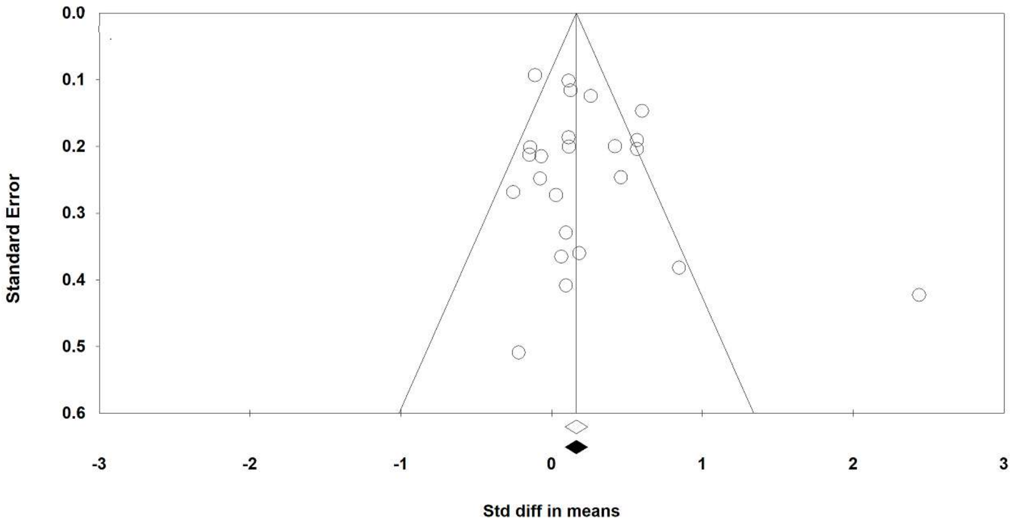

Dunning D, *et al. Evid Based Ment Health* 2022; 25:135–142. doi: 10.1136/ebmental-2022-300464

1.3 Funnel plot for depression outcomes following Mindfulness training

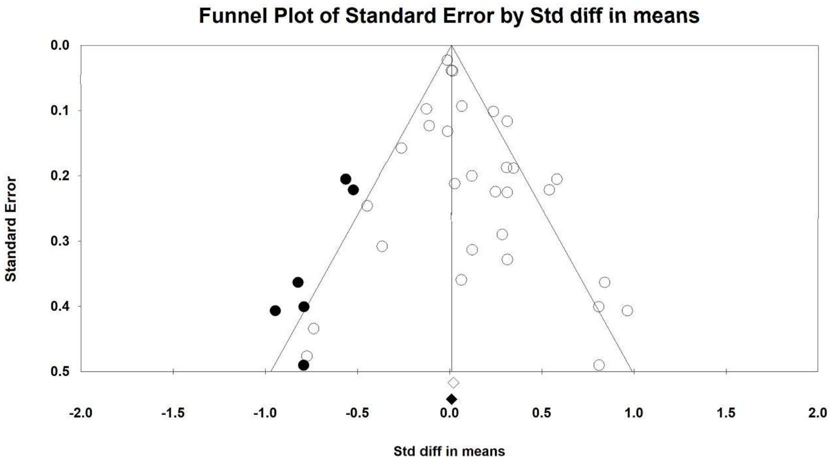

1.4 Funnel plot for executive function outcomes following Mindfulness training

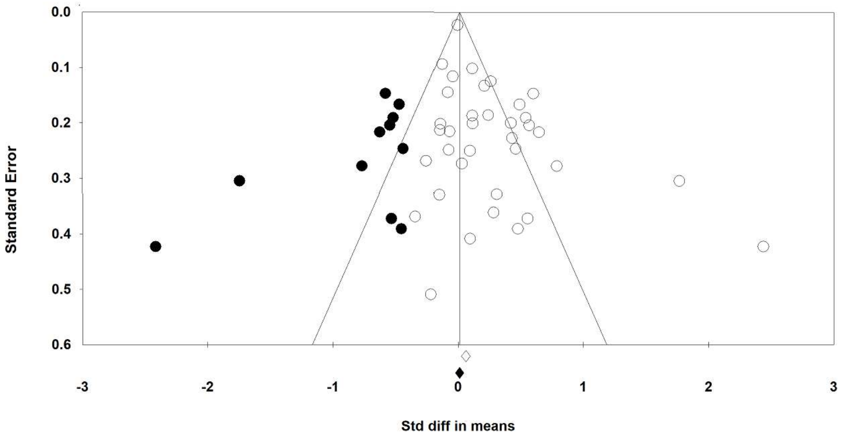

1.5 Funnel plot for mindfulness outcomes following Mindfulness training

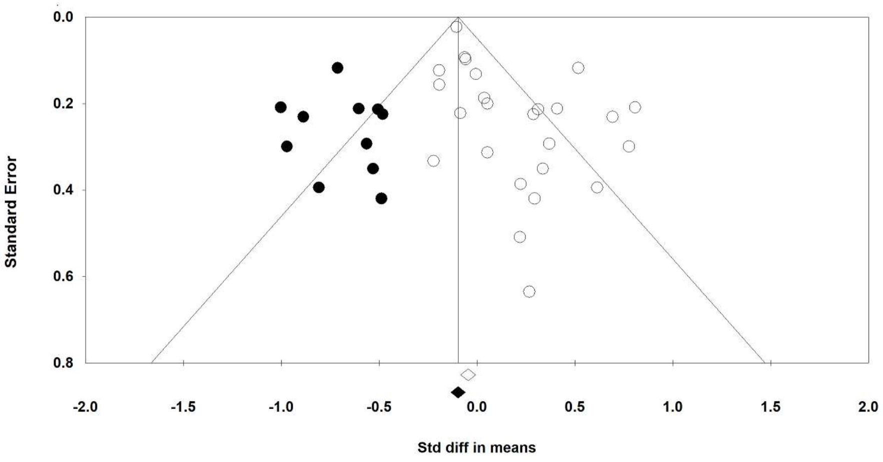

1.6 Funnel plot for negative behaviour outcomes following Mindfulness training

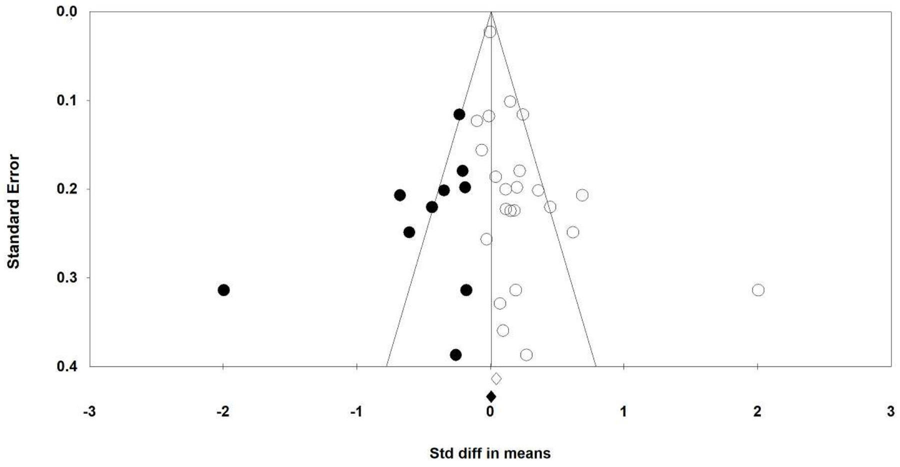

1.7 Funnel plot for social behaviour outcomes following Mindfulness training

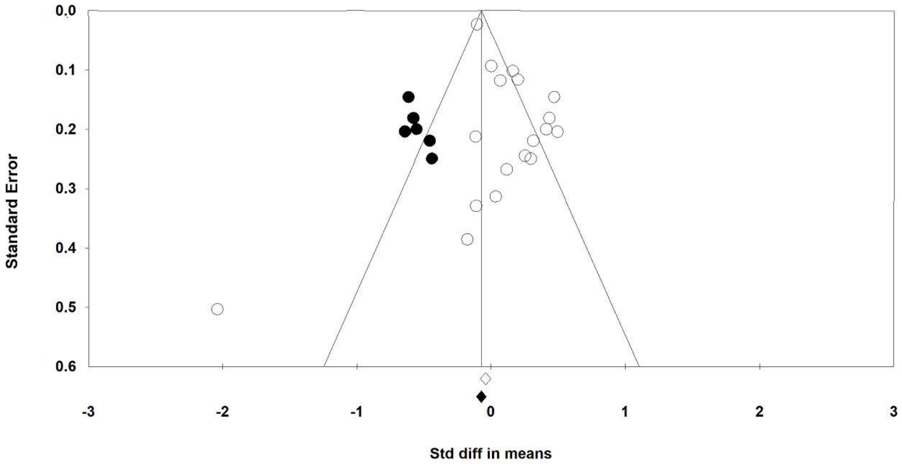

1.8 Funnel plot for wellbeing outcomes following Mindfulness training

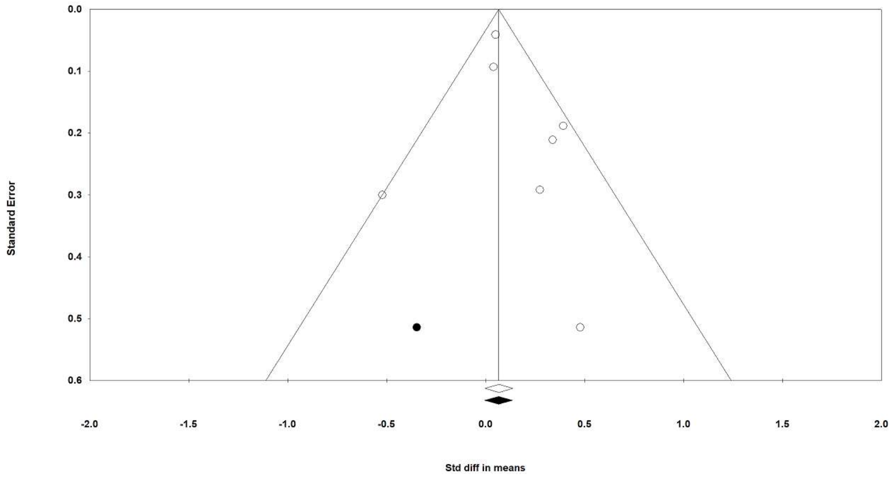

2. Mindfulness versus active controls

2.1 Funnel plot for anxiety/stress outcomes following Mindfulness training compared to active control conditions

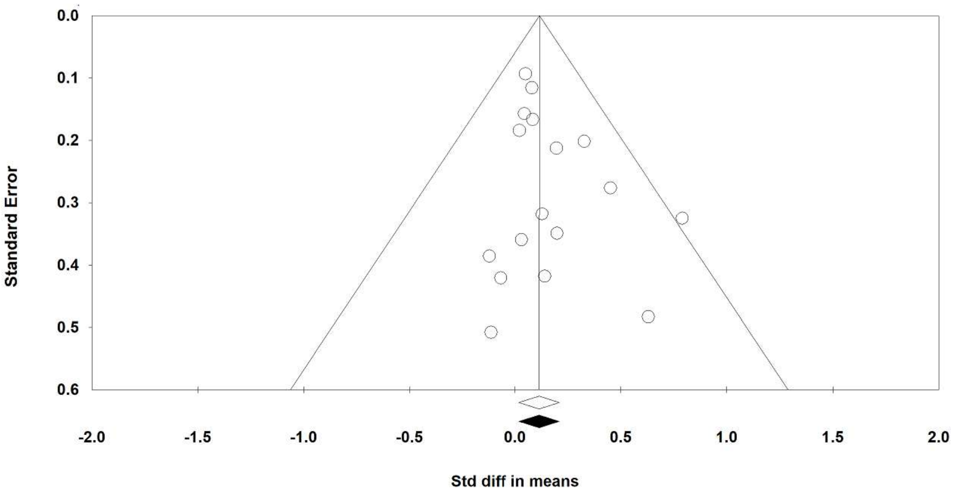

2.2 Funnel plot for attention outcomes following Mindfulness training compared to active control conditions

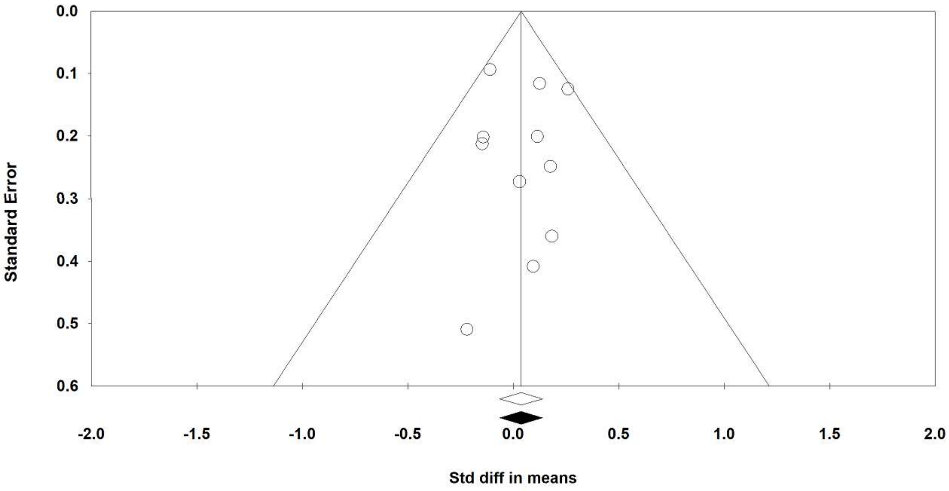

2.3 Funnel plot for depression outcomes following Mindfulness training compared to active control conditions

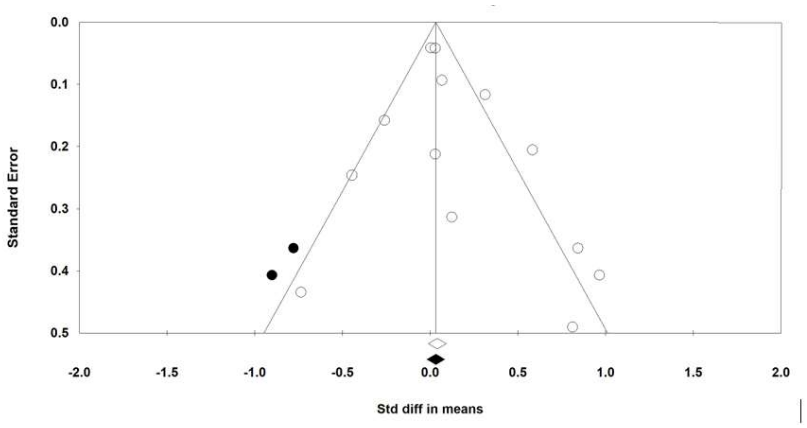

2.4 Funnel plot for Executive functions outcomes following Mindfulness training compared to active control conditions

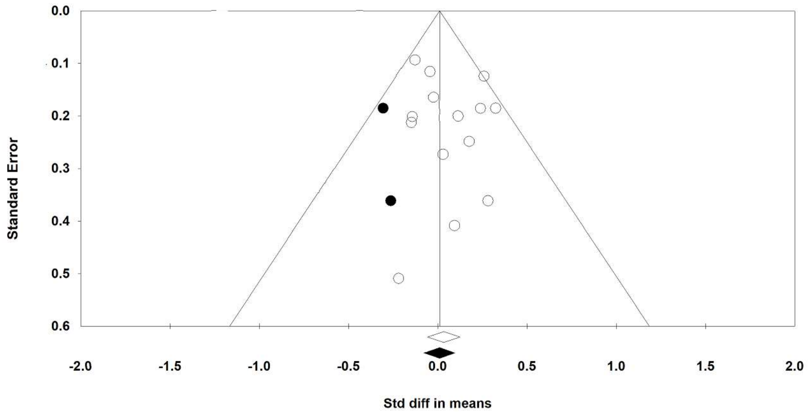

2.5 Funnel plot for mindfulness outcomes following Mindfulness training compared to active control conditions

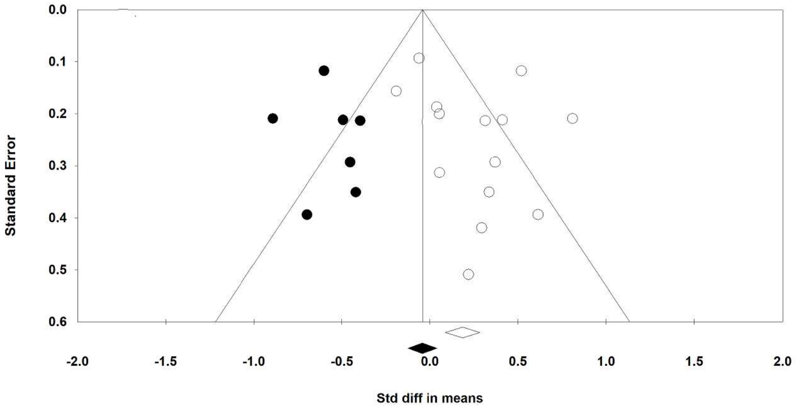

2.6 Funnel plot for negative behaviour outcomes following Mindfulness training compared to active control conditions

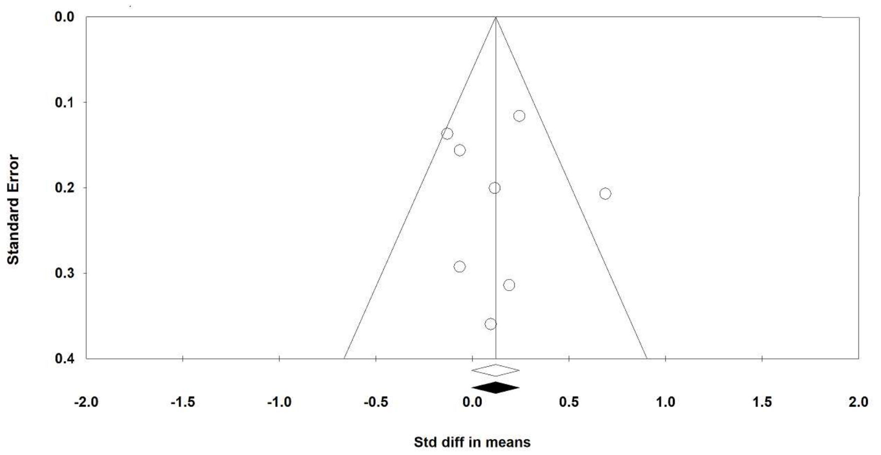

2.7 Funnel plot for social behaviour outcomes following Mindfulness training compared to active control conditions

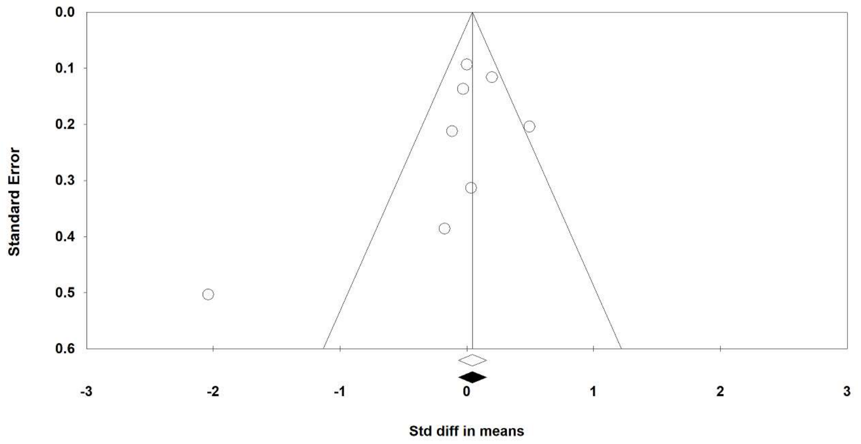

2.8 Funnel plot for wellbeing outcomes following Mindfulness training compared to active control conditions

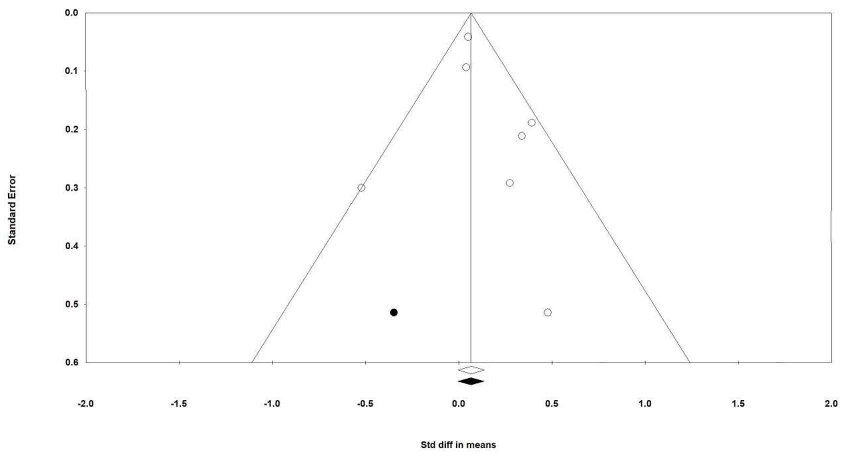

3. Mindfulness compared with passive control groups

3.1 *Funnel plot for anxiety/stress outcomes following Mindfulness training compared to passive control conditions*

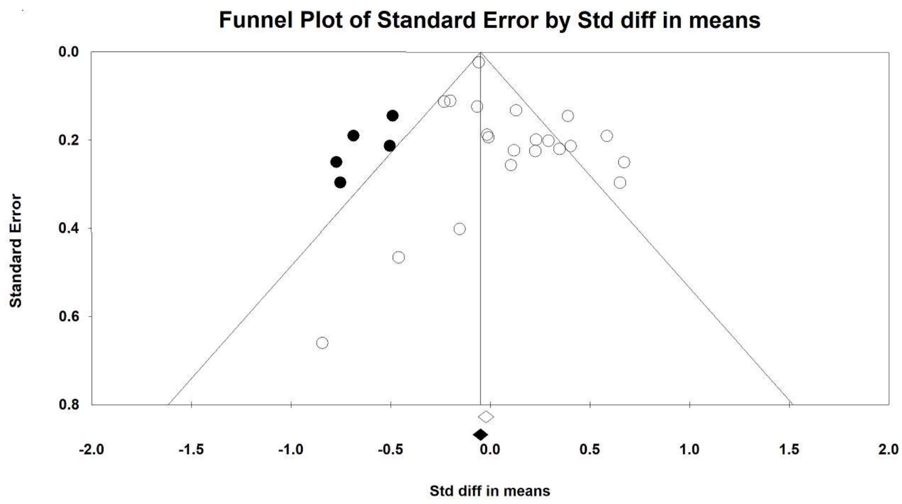

3.2 *Funnel plot for attention outcomes following Mindfulness training compared to passive control conditions*

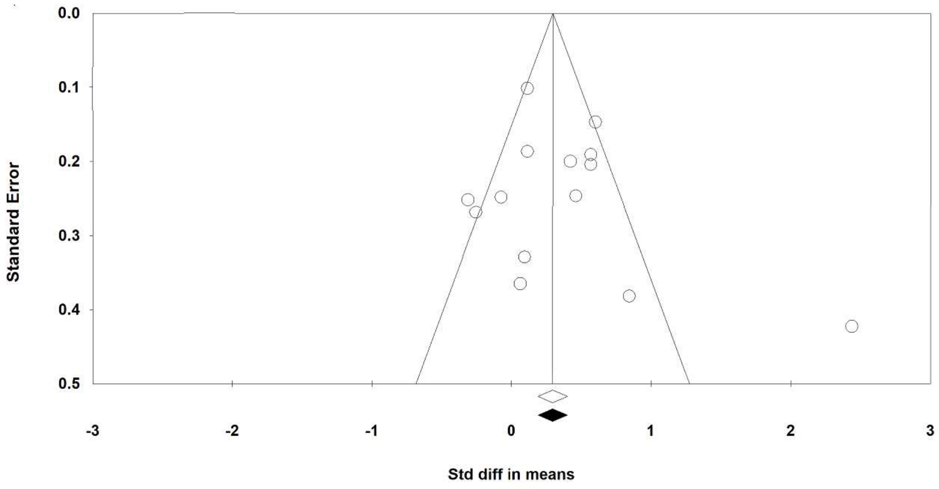

3.3 *Funnel plot for depression outcomes following Mindfulness training compared to passive control conditions*

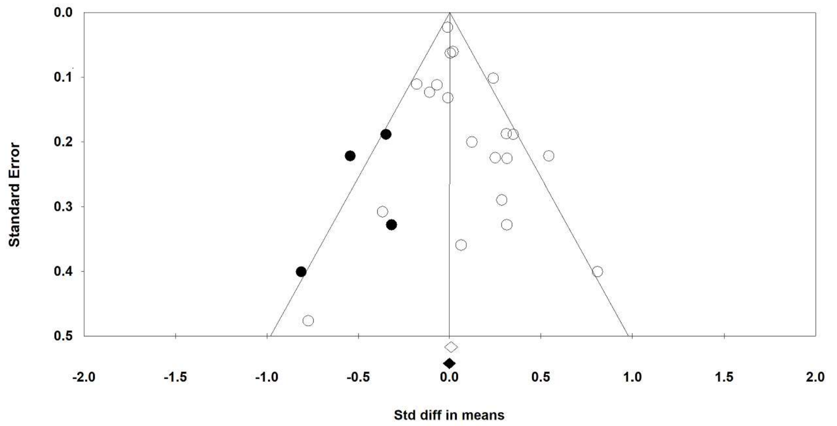

3.4 *Funnel plot for executive function outcomes following Mindfulness training compared to passive control conditions*

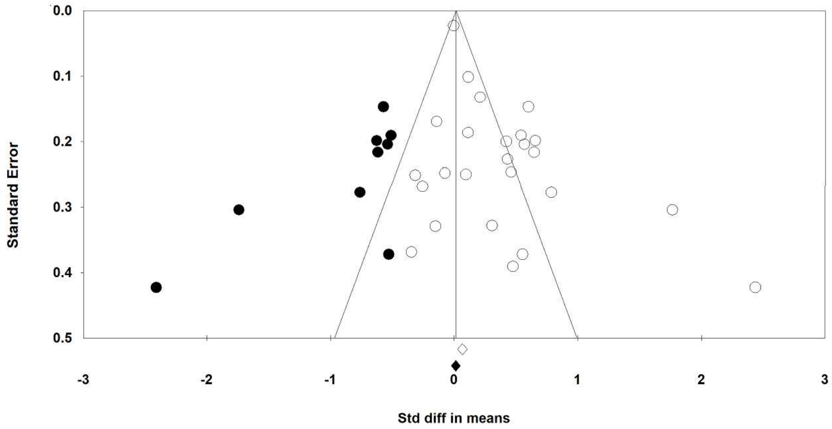

3.5 *Funnel plot for mindfulness outcomes following Mindfulness training compared to passive control conditions*

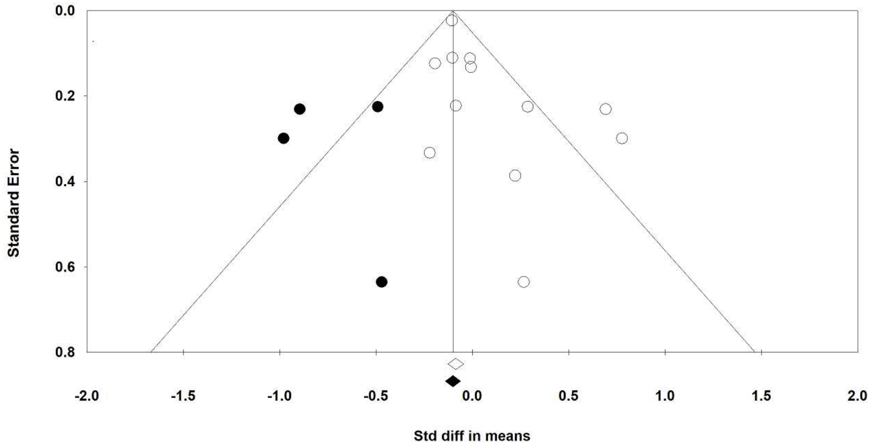

3.6 *Funnel plot for negative behaviour outcomes following Mindfulness training compared to passive control conditions*

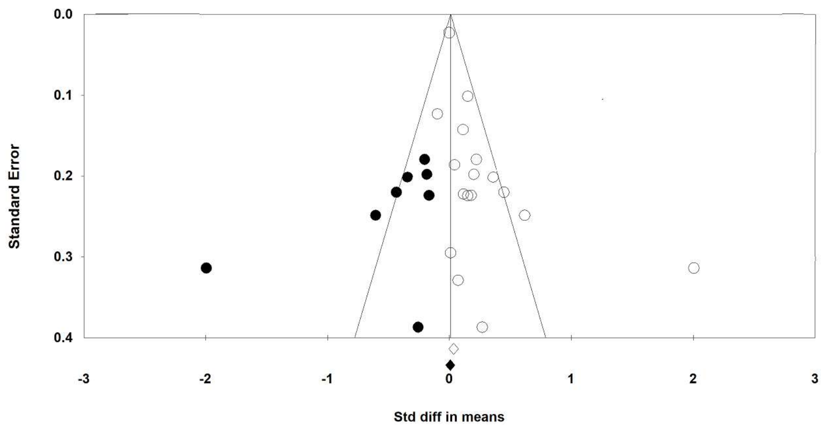

3.7 *Funnel plot for social behaviour outcomes following Mindfulness training compared to passive control conditions*

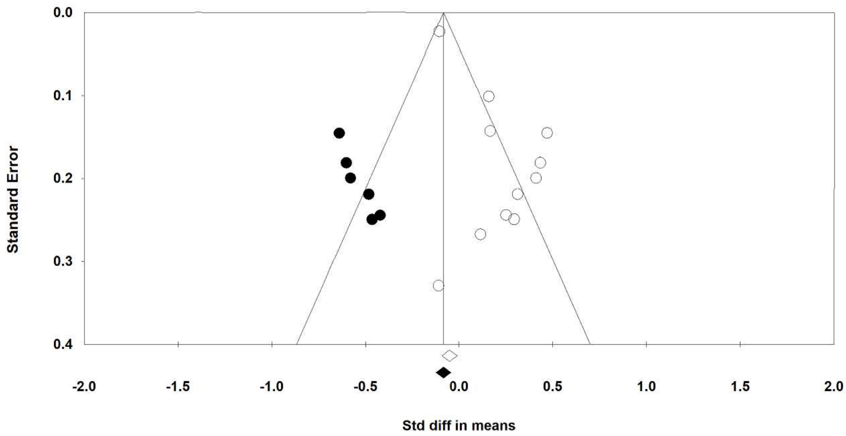

3.8 *Funnel plot for wellbeing outcomes following Mindfulness training compared to passive control conditions*

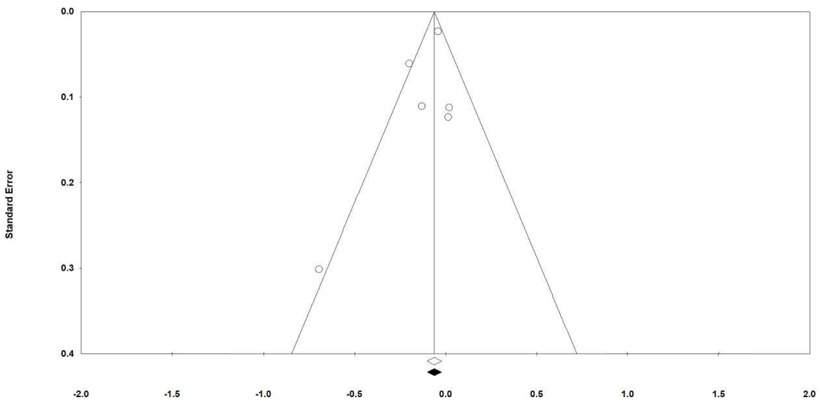

4. <u>Mindfulness as a selective intervention compared to control groups</u>

4.1 *Funnel plot for anxiety/stress outcomes following Mindfulness training as a selective intervention*

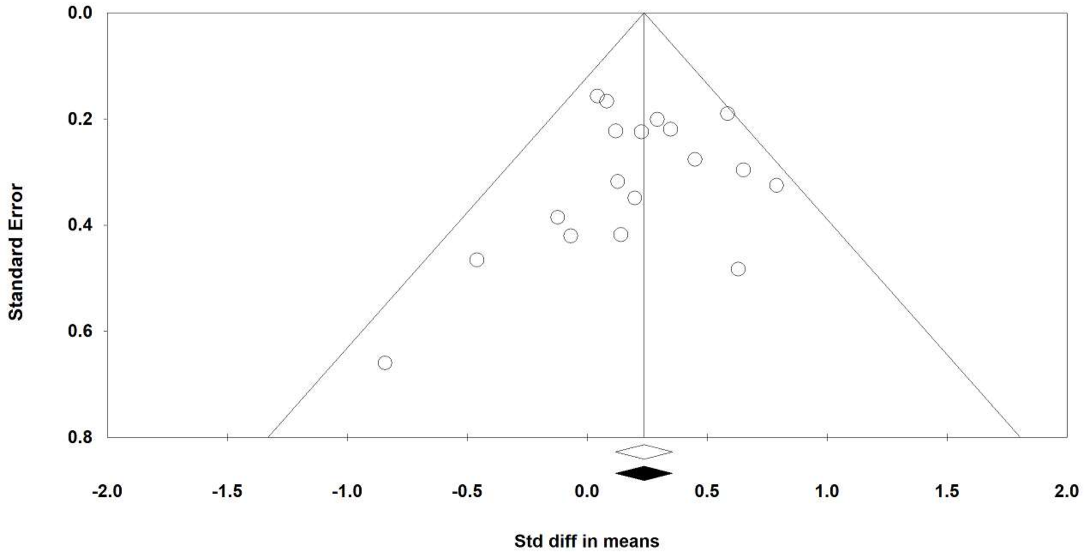

4.2 *Funnel plot for attention outcomes following Mindfulness training as a selective intervention*

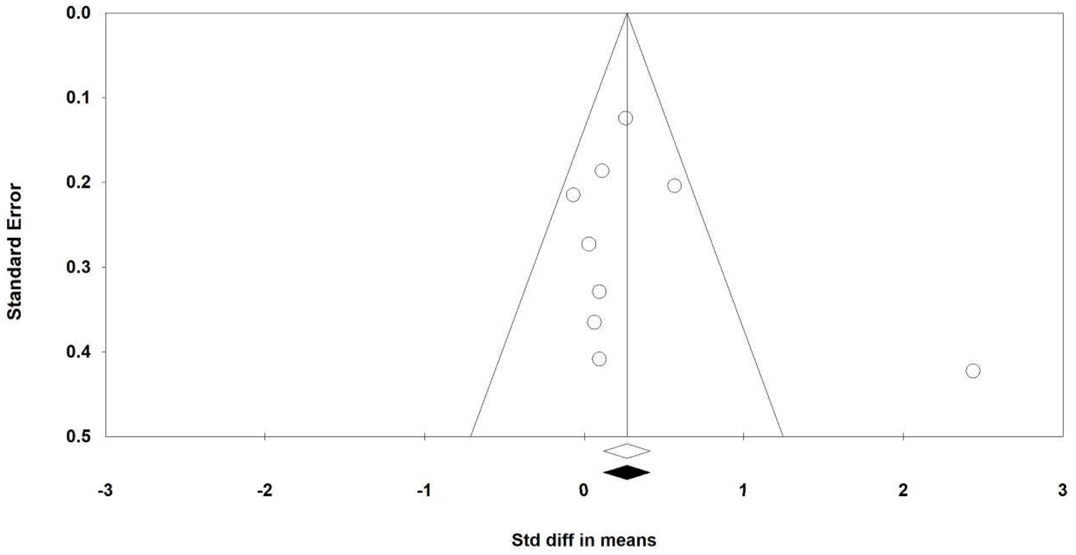

4.3 *Funnel plot for depression outcomes following Mindfulness training as a selective intervention*

Dunning D, *et al. Evid Based Ment Health* 2022; 25:135–142. doi: 10.1136/ebmental-2022-300464

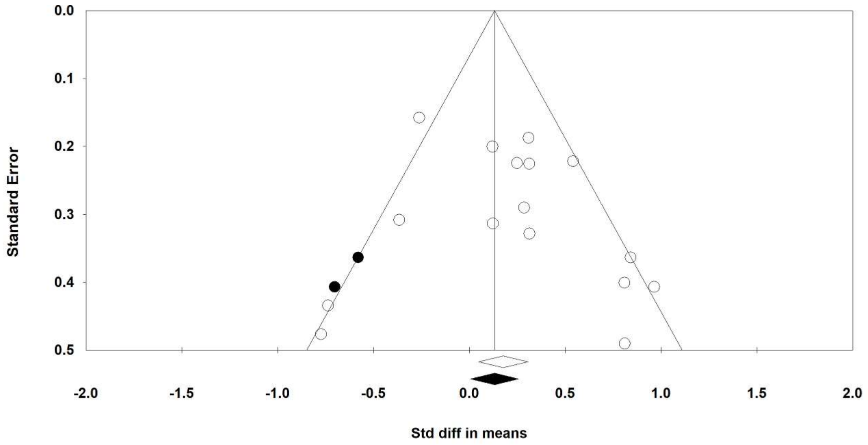

4.4 *Funnel plot for executive function outcomes following Mindfulness training as a selective intervention*

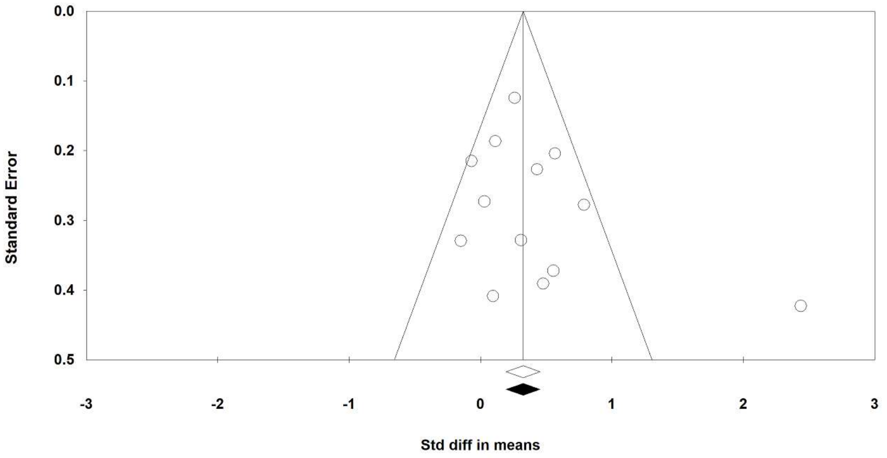

4.5 *Funnel plot for mindfulness outcomes following Mindfulness training as a selective intervention*

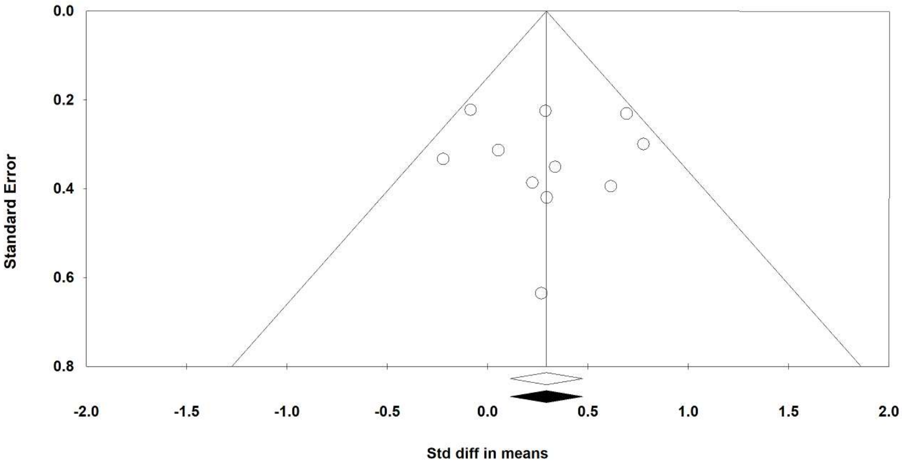

4.6 Funnel plot for negative behaviour outcomes following Mindfulness training as a selective intervention

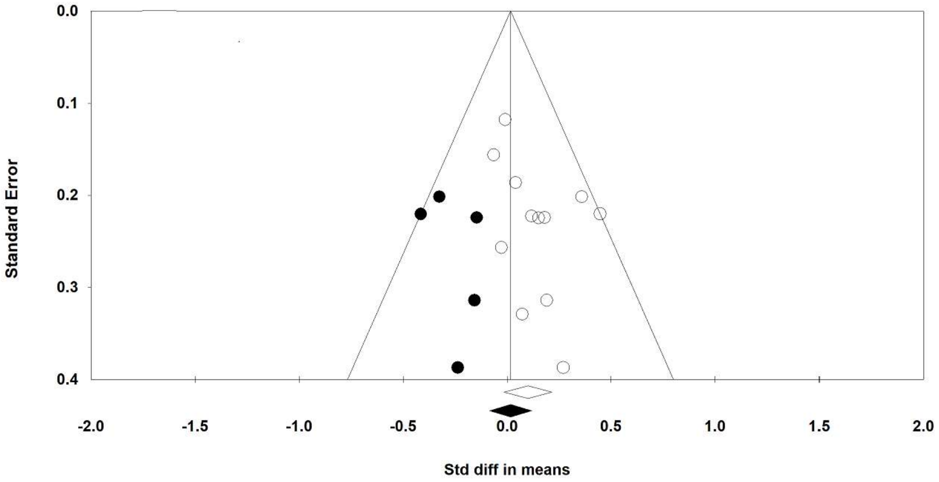

4.1 *Funnel plot for social behaviour outcomes following Mindfulness training as a selective intervention*

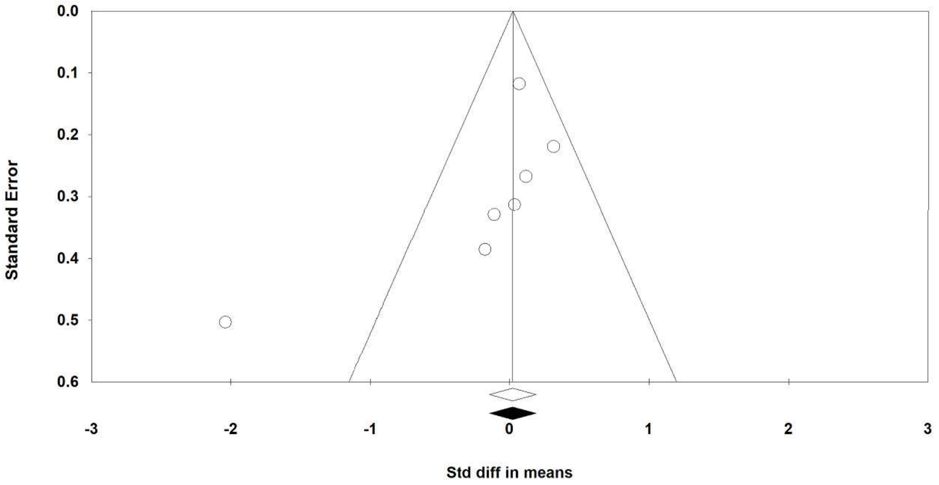

Dunning D, *et al. Evid Based Ment Health* 2022; 25:135–142. doi: 10.1136/ebmental-2022-300464

5. Mindfulness as a universal intervention compared to controls

5.1 *Funnel plot for anxiety/stress outcomes following Mindfulness training as a universal intervention*

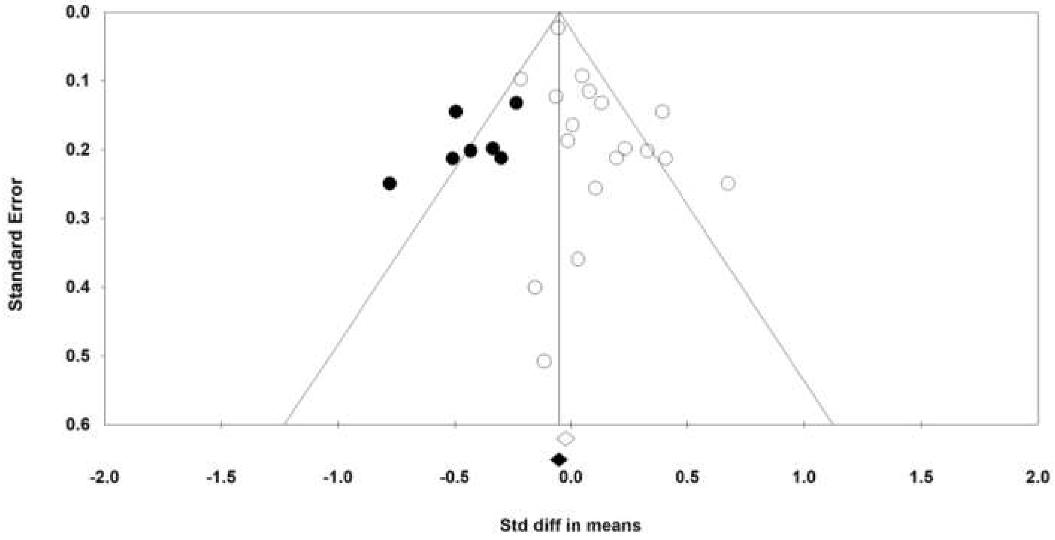

5.2 *Funnel plot for attention outcomes following Mindfulness training as a universal intervention*

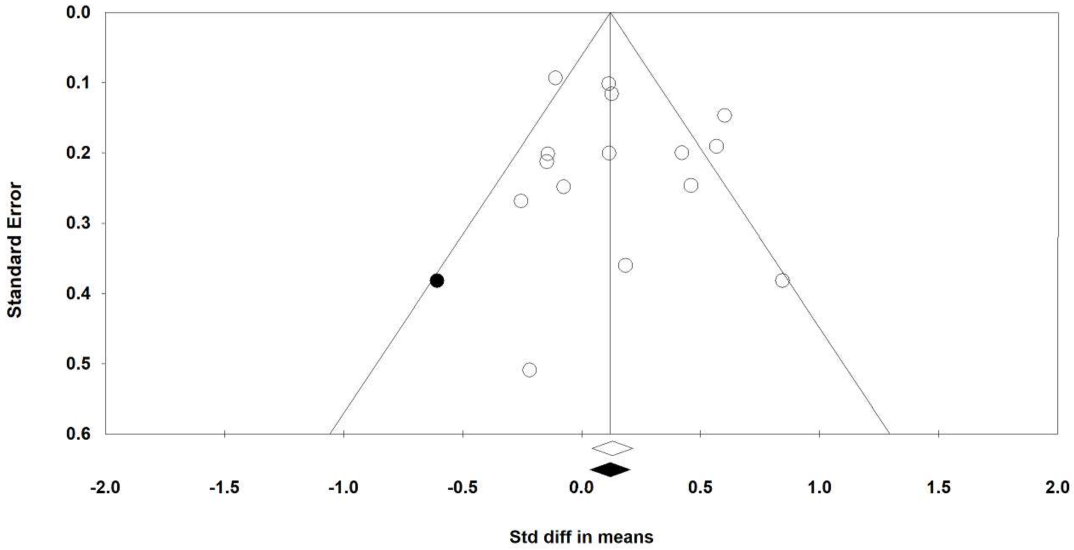

5.3 *Funnel plot for depression outcomes following Mindfulness training as a universal intervention*

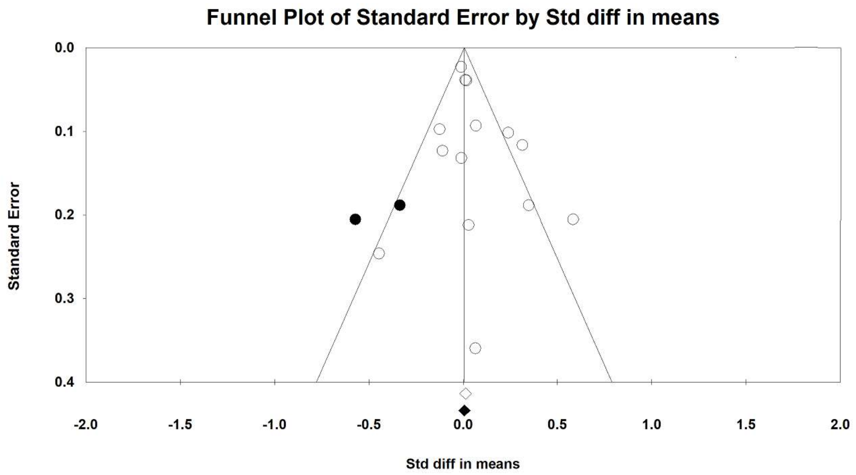

5.4 *Funnel plot for executive function outcomes following Mindfulness training as a universal intervention*

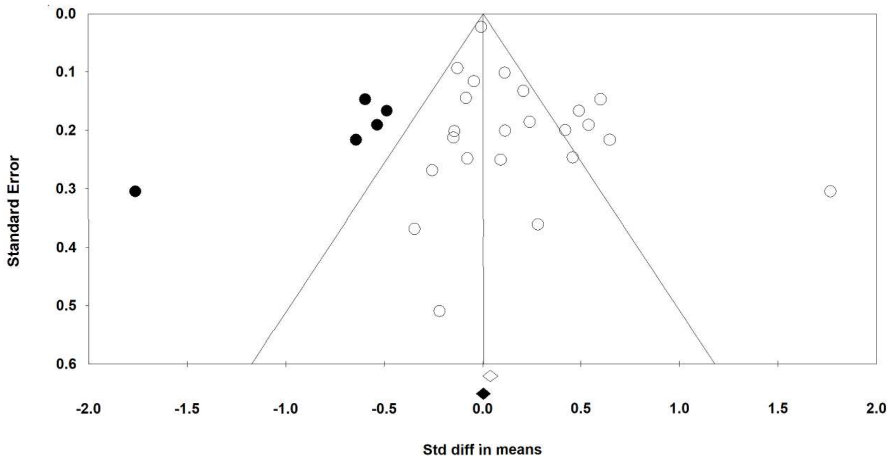

5.5 *Funnel plot for mindfulness outcomes following Mindfulness training as a universal intervention*

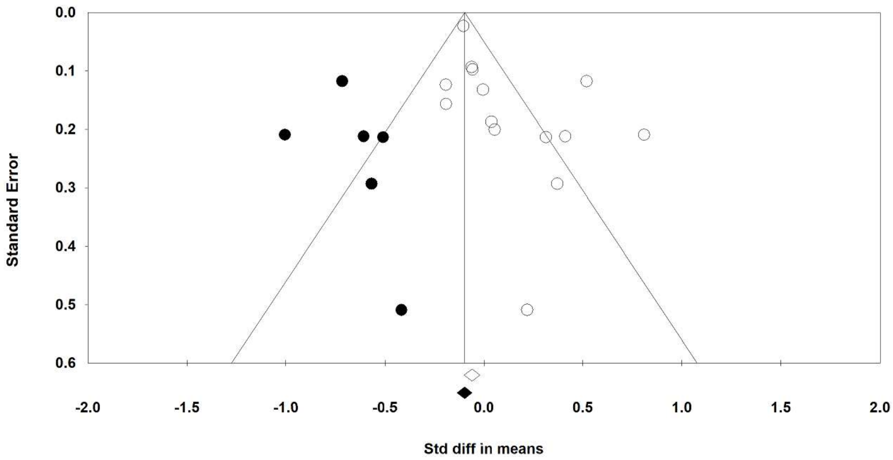

5.6 *Funnel plot for negative behaviour outcomes following Mindfulness training as a universal intervention*

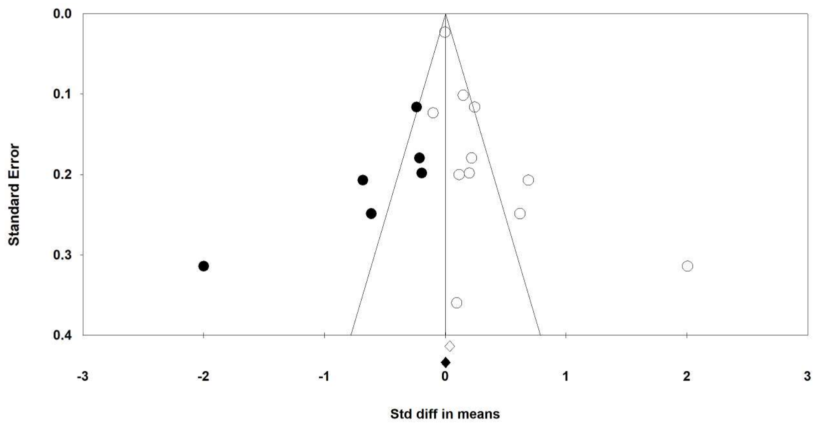

5.7 *Funnel plot for social behaviour outcomes following Mindfulness training as a universal intervention*

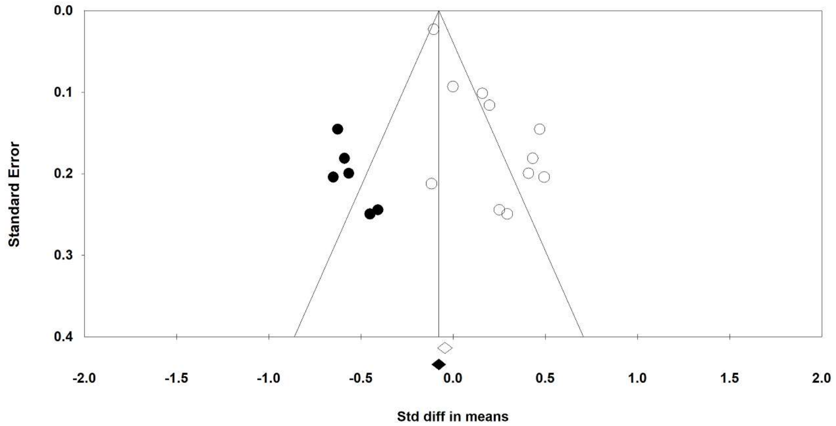

5.8 *Funnel plot for wellbeing outcomes following Mindfulness training as a universal intervention*

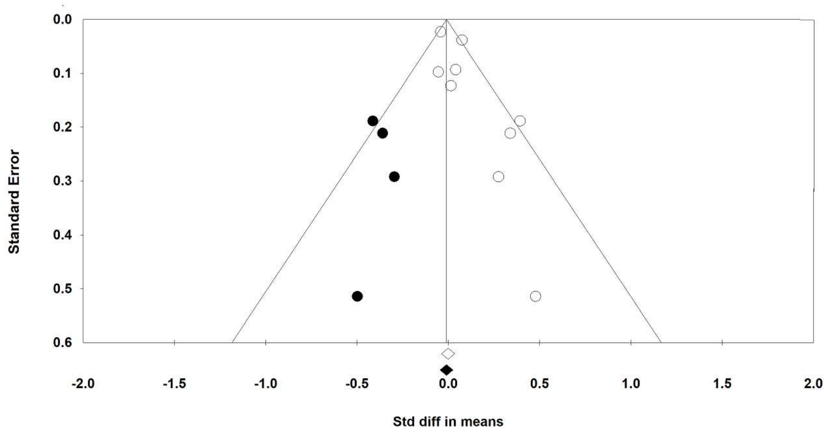

Supplement H – Supplemental references

References

1. Beck AT, Steer RA, Brown GK. Beck depression inventory (BDI-II): Pearson London, UK; 1996.

2. Wang Y-P, Gorenstein C. Psychometric properties of the Beck Depression Inventory-II: a comprehensive review. Brazilian Journal of Psychiatry. 2013;35:416-31.

3. Osman A, Barrios FX, Gutierrez PM, Williams JE, Bailey J. Psychometric properties of the Beck Depression Inventory-II in nonclinical adolescent samples. Journal of clinical psychology. 2008;64(1):83-102.

4. Beck JS, Beck AT, Jolly JB. Beck youth inventories of emotional & social impairment: Depression inventory for youth, anxiety inventory for youth, anger inventory for youth, disruptive behavior inventory for youth, self-concept inventory for youth: Manual: Psychological Corporation; 2001.

5. Bose-Deakins JE, Floyd RG. A review of the Beck Youth Inventories of emotional and social impairment. Journal of School Psychology. 2004;42(4):333-40.

6. Thastum M, Ravn K, Sommer S, Trillingsgaard A. Reliability, validity and normative data for the Danish Beck Youth Inventories. Scandinavian Journal of Psychology. 2009;50(1):47-54.

7. Reynolds CR. Behavior assessment system for children. The Corsini encyclopedia of psychology. 2010:1-2.

8. Eaton WW, Smith C, Ybarra M, Muntaner C, Tien A. Center for Epidemiologic Studies Depression Scale: review and revision (CESD and CESD-R). 2004.

9. Van Dam NT, Earleywine M. Validation of the Center for Epidemiologic Studies Depression Scale—Revised (CESD-R): Pragmatic depression assessment in the general population. Psychiatry Research. 2011;186(1):128-32.

10. Hensley VR. Australian normative study of the Achenbach child behaviour checklist. Australian Psychologist. 1988;23(3):371-82.

11. Kariuki SM, Abubakar A, Murray E, Stein A, Newton CR. Evaluation of psychometric properties and factorial structure of the pre-school child behaviour checklist at the Kenyan Coast. Child and adolescent psychiatry and mental health. 2016;10(1):1-10.

12. Nøvik TS. Validity of the Child Behaviour Checklist in a Norwegian sample. European Child & Adolescent Psychiatry. 1999;8(4):247-54.

13. Foa EB, Johnson KM, Feeny NC, Treadwell KRH. The Child PTSD Symptom Scale: A Preliminary Examination of its Psychometric Properties. Journal of Clinical Child & Adolescent Psychology. 2001;30(3):376-84.

14. Abela JR, Vanderbilt E, Rochon A. A test of the integration of the response styles and social support theories of depression in third and seventh grade children. Journal of Social and Clinical Psychology. 2004;23(5):653-74.

15. Abela JRZ, Aydin CM, Auerbach RP. Responses to Depression in Children: Reconceptualizing the Relation Among Response Styles. Journal of Abnormal Child Psychology. 2007;35(6):913-27.

Dunning D, *et al. Evid Based Ment Health* 2022; 25:135–142. doi: 10.1136/ebmental-2022-300464

16. Lovibond PF, Lovibond SH. The structure of negative emotional states: Comparison of the Depression Anxiety Stress Scales (DASS) with the Beck Depression and Anxiety Inventories. Behaviour research and therapy. 1995;33(3):335-43.

17. Clara IP, Cox BJ, Enns MW. Confirmatory Factor Analysis of the Depression–Anxiety–Stress Scales in Depressed and Anxious Patients. Journal of Psychopathology and Behavioral Assessment. 2001;23(1):61-7.

18. Patrick J, Dyck M, Bramston P. Depression Anxiety Stress Scale: is it valid for children and adolescents? Journal of clinical psychology. 2010;66(9):996-1007.

19. Szabó M. The short version of the Depression Anxiety Stress Scales (DASS-21): Factor structure in a young adolescent sample. Journal of adolescence. 2010;33(1):1-8.

20. Izard CE, Libero DZ, Putnam P, Haynes OM. Stability of emotion experiences and their relations to traits of personality. Journal of personality and social psychology. 1993;64(5):847.

21. Youngstrom EA, Green KW. Reliability generalization of self-report of emotions when using the Differential Emotions Scale. Educational and Psychological Measurement. 2003;63(2):279-95.

22. Ravens-Sieberer U, Gosch A, Rajmil L, Erhart M, Bruil J, Duer W, et al. KIDSCREEN-52 quality-of-life measure for children and adolescents. Expert review of pharmacoeconomics & outcomes research. 2005;5(3):353-64.

23. Bullinger M, Brütt AL, Erhart M, Ravens-Sieberer U, the BSG. Psychometric properties of the KINDL-R questionnaire: results of the BELLA study. European Child & Adolescent Psychiatry. 2008;17(1):125-32.

24. Kroenke K, Spitzer RL, Williams JB. The PHQ-9: validity of a brief depression severity measure. Journal of general internal medicine. 2001;16(9):606-13.

25. Richardson LP, McCauley E, Grossman DC, McCarty CA, Richards J, Russo JE, et al. Evaluation of the Patient Health Questionnaire-9 Item for detecting major depression among adolescents. Pediatrics. 2010;126(6):1117-23.

26. Burdzovic Andreas J, Brunborg GS. Depressive symptomatology among Norwegian adolescent boys and girls: the patient health Questionnaire-9 (PHQ-9) psychometric properties and correlates. Frontiers in psychology. 2017;8:887.

27. Osman A, Gutierrez PM, Kopper BA, Barrios FX, Chiros CE. The Positive and Negative Suicide Ideation Inventory: Development and Validation. Psychological Reports. 1998;82(3):783-93.

28. Muehlenkamp JJ, Gutierrez PM, Osman A, Barrios FX. Validation of the Positive and Negative Suicide Ideation (PANSI) Inventory in a diverse sample of young adults. Journal of clinical psychology. 2005;61(4):431-45.

29. Nolen-Hoeksema S, Morrow J. A prospective study of depression and posttraumatic stress symptoms after a natural disaster: the 1989 Loma Prieta Earthquake. Journal of personality and social psychology. 1991;61(1):115.

30. Treynor W, Gonzalez R, Nolen-Hoeksema S. Rumination reconsidered: A psychometric analysis. Cognitive therapy and research. 2003;27(3):247-59.

31. Chorpita BF, Yim L, Moffitt C, Umemoto LA, Francis SE. Assessment of symptoms of DSM-IV anxiety and depression in children: a revised child anxiety and depression scale. Behaviour Research and Therapy. 2000;38(8):835-55.

32. Piqueras JA, Martín-Vivar M, Sandin B, San Luis C, Pineda D. The Revised Child Anxiety and Depression Scale: A systematic review and reliability generalization meta-analysis. Journal of Affective Disorders. 2017;218:153-69.

33. Rosenberg M. Rosenberg self-esteem scale. Journal of Religion and Health. 1965.

34. Robins RW, Hendin HM, Trzesniewski KH. Measuring global self-esteem: Construct validation of a single-item measure and the Rosenberg Self-Esteem Scale. Personality and social psychology bulletin. 2001;27(2):151-61.

35. Rains C. Seattle Personality Questionnaire—Original (fast track project technical report). Retrieved October. 2005.

36. Derogatis LR, Unger R. Symptom checklist-90-revised. The Corsini encyclopedia of psychology. 2010:1-2.

37. Müller JM, Postert C, Beyer T, Furniss T, Achtergarde S. Comparison of Eleven Short Versions of the Symptom Checklist 90-Revised (SCL-90-R) for Use in the Assessment of General Psychopathology. Journal of Psychopathology and Behavioral Assessment. 2010;32(2):246-54.

38. Hart SR, Stewart K, Jimerson SR. Teacher Engagement Report Form-New. Contemporary School Psychology.

39. Hart SR, Stewart K, Jimerson SR. The student engagement in schools questionnaire (SESQ) and the teacher engagement report form-new (TERF-N): Examining the preliminary evidence. Contemporary School Psychology: Formerly" The California School Psychologist". 2011;15(1):67-79.

40. Kovacs M. Children’s depression inventory. Acta Paedopsychiatrica: International Journal of Child & Adolescent Psychiatry. 1992.

41. Masip AF, Amador-Campos JA, Gómez-Benito J, del Barrio Gándara V. Psychometric properties of the Children's Depression Inventory in community and clinical sample. The Spanish journal of psychology. 2010;13(2):990-9.

42. Angold A, Costello EJ, Messer SC, Pickles A. Development of a short questionnaire for use in epidemiological studies of depression in children and adolescents. International journal of methods in psychiatric research. 1995.

43. Kent L, Vostanis P, Feehan C. Detection of major and minor depression in children and adolescents: evaluation of the Mood and Feelings Questionnaire. Journal of Child Psychology and Psychiatry. 1997;38(5):565-73.

44. Xiang Y, Cuijpers P, Teng T, Li X, Fan L, Liu X, et al. Comparative short-term efficacy and acceptability of a combination of pharmacotherapy and psychotherapy for depressive disorder in children and adolescents: a systematic review and meta-analysis. BMC psychiatry. 2022;22(1):1-13.

45. Muris P, Meesters C, Spinder M. Relationships between child- and parent-reported behavioural inhibition and symptoms of anxiety and depression in normal adolescents. Personality and Individual Differences. 2003;34(5):759-71.

46. Hope TL, Adams C, Reynolds L, Powers D, Perez RA, Kelley ML. Parent vs. self-report: Contributions toward diagnosis of adolescent psychopathology. Journal of Psychopathology and Behavioral Assessment. 1999;21(4):349-63.

47. Toplak ME, West RF, Stanovich KE. Practitioner review: Do performance-based measures and ratings of executive function assess the same construct? Journal of child psychology and psychiatry. 2013;54(2):131-43.

48. Buchanan T. Self-report measures of executive function problems correlate with personality, not performance-based executive function measures, in nonclinical samples. Psychological Assessment. 2016;28(4):372.

49. Byrne DG, Davenport S, Mazanov J. Profiles of adolescent stress: The development of the adolescent stress questionnaire (ASQ). Journal of adolescence. 2007;30(3):393-416.

50. De Vriendt T, Clays E, Moreno LA, Bergman P, Vicente-Rodriguez G, Nagy E, et al. Reliability and validity of the Adolescent Stress Questionnaire in a sample of European adolescents - the HELENA study. BMC Public Health. 2011;11(1):717.

51. Spitzer RL, Kroenke K, Williams JB, Löwe B. A brief measure for assessing generalized anxiety disorder: the GAD-7. Archives of internal medicine. 2006;166(10):1092-7.

52. Kroenke K, Spitzer RL, Williams JB, Löwe B. The patient health questionnaire somatic, anxiety, and depressive symptom scales: a systematic review. General hospital psychiatry. 2010;32(4):345-59.

53. Mossman SA, Luft MJ, Schroeder HK, Varney ST, Fleck DE, Barzman DH, et al. The Generalized Anxiety Disorder 7-item (GAD-7) scale in adolescents with generalized anxiety disorder: signal detection and validation. Annals of clinical psychiatry: official journal of the American Academy of Clinical Psychiatrists. 2017;29(4):227.

54. March JS, Parker JDA, Sullivan K, Stallings P, Conners CK. The Multidimensional Anxiety Scale for Children (MASC): Factor Structure, Reliability, and Validity. Journal of the American Academy of Child & Adolescent Psychiatry. 1997;36(4):554-65.

55. Baldwin JS, Dadds MR. Reliability and Validity of Parent and Child Versions of the Multidimensional Anxiety Scale for Children in Community Samples. Journal of the American Academy of Child & Adolescent Psychiatry. 2007;46(2):252-60.

56. March JS, Sullivan K, Parker J. Test-retest reliability of the Multidimensional Anxiety Scale for Children. Journal of anxiety disorders. 1999;13(4):349-58.

57. Cohen S, Kamarck T, Mermelstein R. A global measure of perceived stress. Journal of health and social behavior. 1983:385-96.

58. Lee E-H. Review of the Psychometric Evidence of the Perceived Stress Scale. Asian Nursing Research. 2012;6(4):121-7.

59. Kechter A, Black DS, Riggs NR, Warren CM, Ritt-Olson A, Chou C-P, et al. Factors in the perceived stress scale differentially associate with mindfulness disposition and executive function among early adolescents. Journal of child and family studies. 2019;28(3):814-21.

60. Griffin DW, Bartholomew K. Relationship scales questionnaire. Journal of Personality and Social Psychology. 1994.

61. Zortea TC, Gray CM, O'Connor RC. Adult attachment: Investigating the factor structure of the Relationship Scales Questionnaire. Journal of clinical psychology. 2019;75(12):2169-87.

62. Birmaher B, Khetarpal S, Brent D, Cully M, Balach L, Kaufman J, et al. The Screen for Child Anxiety Related Emotional Disorders (SCARED): Scale Construction and Psychometric Characteristics. Journal of the American Academy of Child & Adolescent Psychiatry. 1997;36(4):545-53.

63. La Greca AM, Stone WL. Social anxiety scale for children-revised: Factor structure and concurrent validity. Journal of clinical child psychology. 1993;22(1):17-27.

64. Walker C, Kaufman K, Keyser D, Sweetland R. Review of the State-Trait Anxiety Inventory for Children. DJ Keyser & RC Sweetland, RC (Eds), Test Critiques. 1984;1:633-40.

65. Seligman LD, Ollendick TH, Langley AK, Baldacci HB. The utility of measures of child and adolescent anxiety: A meta-analytic review of the Revised Children's Manifest Anxiety Scale, the State–Trait Anxiety Inventory for Children, and the Child Behavior Checklist. Journal of Clinical Child and Adolescent Psychology. 2004;33(3):557-65.

66. Sarason IG. The test anxiety scale: Concept and research. 1977.

67. Lowe PA, Grumbein MJ, Raad JM. Examination of the psychometric properties of the Test Anxiety Scale for Elementary Students (TAS-E) scores. Journal of Psychoeducational Assessment. 2011;29(6):503-14.

68. Conners CK, Sitarenios G, Parker JDA, Epstein JN. Revision and Restandardization of the Conners Teacher Rating Scale (CTRS-R): Factor Structure, Reliability, and Criterion Validity. Journal of Abnormal Child Psychology. 1998;26(4):279-91.

69. Reynolds CR, Richmond BO. Factor structure and construct validity of'What I think and feel': The revised children's manifest anxiety scale. Journal of Personality Assessment. 1979;43(3):281-3.

70. Wisniewski JJ, Genshaft JL, Mulick JA, Coury DL. Test-Retest Reliability of the Revised Children's Manifest Anxiety Scale. Perceptual and Motor Skills. 1987;65(1):67-70.

71. Reynolds CR. Concurrent validity of what I think and feel: The revised children's manifest anxiety scale. Journal of consulting and clinical psychology. 1980;48(6):774.

72. Deschesnes M. Étude de la validité et de la fidélité de l'Indice de détresse psychologique de Santé Québec (IDPSQ-14), chez une population adolescente. Canadian Psychology/Psychologie canadienne. 1998;39(4):288.

73. Tennant R, Hiller L, Fishwick R, Platt S, Joseph S, Weich S, et al. The Warwick-Edinburgh Mental Well-being Scale (WEMWBS): development and UK validation. Health and Quality of Life Outcomes. 2007;5(1):63.

74. Clarke A, Friede T, Putz R, Ashdown J, Martin S, Blake A, et al. Warwick-Edinburgh Mental Well-being Scale (WEMWBS): Validated for teenage school students in England and Scotland. A mixed methods assessment. BMC Public Health. 2011;11(1):487.

75. Liddle I, Carter GF. Emotional and psychological well-being in children: the development and validation of the Stirling Children's Well-being Scale. Educational Psychology in Practice. 2015;31(2):174-85.

76. Renshaw TL, Long AC, Cook CR. Assessing adolescents' positive psychological functioning at school: Development and validation of the Student Subjective Wellbeing Questionnaire. School psychology quarterly. 2015;30(4):534.

77. Greco LA, Baer RA, Smith GT. Assessing mindfulness in children and adolescents: development and validation of the Child and Adolescent Mindfulness Measure (CAMM). Psychological assessment. 2011;23(3):606.

78. Kuby AK, McLean N, Allen K. Validation of the Child and Adolescent Mindfulness Measure (CAMM) with Non-Clinical Adolescents. Mindfulness. 2015;6(6):1448-55.

79. Kumar SM. Grieving mindfully: A compassionate and spiritual guide to coping with loss: New Harbinger Publications; 2005.

80. Feldman G, Westine M, Edelman A, Higgs M, Renna M, Greeson J. Cognitive and Affective Mindfulness Scale-Revised (CAMS-R). Handbook of Assessment in Mindfulness Research: Springer; 2022. p. 1-24.

81. Brown KW, Ryan RM. The benefits of being present: mindfulness and its role in psychological well-being. Journal of personality and social psychology. 2003;84(4):822.

82. Brown KW, West AM, Loverich TM, Biegel GM. Assessing adolescent mindfulness: validation of an adapted Mindful Attention Awareness Scale in adolescent normative and psychiatric populations. Psychological assessment. 2011;23(4):1023.

83. Neff KD. The development and validation of a scale to measure self-compassion. Self and identity. 2003;2(3):223-50.

84. Cunha M, Xavier A, Castilho P. Understanding self-compassion in adolescents: Validation study of the Self-Compassion Scale. Personality and Individual Differences. 2016;93:56-62.

85. Johnson C, Burke C, Brinkman S, Wade T. Development and validation of a multifactor mindfulness scale in youth: The Comprehensive Inventory of Mindfulness Experiences–Adolescents (CHIME-A). Psychological assessment. 2017;29(3):264.

86. Davis MH. A multidimensional approach to individual differences in empathy. 1980.

87. Hawk ST, Keijsers L, Branje SJ, Graaff JVd, Wied Md, Meeus W. Examining the interpersonal reactivity index (IRI) among early and late adolescents and their mothers. Journal of personality assessment. 2013;95(1):96-106.

88. Ullmann RK, Sleator EK, Sprague R. Add-h comprehensive teacher's rating scale (acters). Los Angeles, CA: Western Psychological Services. 1991.

89. Carlini RJ, Parks TW. ADD-H comprehensive teacher's rating scale. Journal of Psychoeducational Assessment. 1993;11(1):95-7.

90. Bretherton I, Prentiss C, Ridgeway D. Family relationships as represented in a story-completion task at thirty-seven and fifty-four months of age. New Directions for Child and Adolescent Development. 1990;1990(48):85-105.

91. Bohn K, Fairburn CG. The clinical impairment assessment questionnaire (CIA). Cognitive behavioral therapy for eating disorders. 2008:1-3.

92. Reas DL, Rø Ø, Kapstad H, Lask B. Psychometric properties of the clinical impairment assessment: norms for young adult women. International Journal of Eating Disorders. 2010;43(1):72-6.

93. Poehlmann-Tynan J, Vigna AB, Weymouth LA, Gerstein ED, Burnson C, Zabransky M, et al. A Pilot Study of Contemplative Practices with Economically Disadvantaged Preschoolers: Children's Empathic and Self-Regulatory Behaviors. Mindfulness. 2016;7(1):46-58.

94. Zahn-Waxler C, Robinson JL, Emde RN. The development of empathy in twins. Dev Psychol. 1992;28(6):1038.

95. Flook L, Goldberg SB, Pinger L, Davidson RJ. Promoting prosocial behavior and self-regulatory skills in preschool children through a mindfulness-based Kindness Curriculum. Dev Psychol. 2015;51(1):44-51.

96. Anderman LH, Anderman EM. Social predictors of changes in students' achievement goal orientations. Contemporary educational psychology. 1999;24(1):21-37.

97. Thompson JK, Van Den Berg P, Roehrig M, Guarda AS, Heinberg LJ. The sociocultural attitudes towards appearance scale-3 (SATAQ-3): Development and validation. International journal of eating disorders. 2004;35(3):293-304.

98. Goodman R. The Strengths and Difficulties Questionnaire: a research note. Journal of child psychology and psychiatry. 1997;38(5):581-6.

99. Muris P, Meesters C, van den Berg F. The Strengths and Difficulties Questionnaire (SDQ). European Child & Adolescent Psychiatry. 2003;12(1):1-8.

100. Group CPPR. Teacher Social Competence Scale Technical Report. 1995. Retrieved from the Fast Track Project website: http://www fasttrackproject org.

101. Lee RM, Robbins SB. Measuring belongingness: The social connectedness and the social assurance scales. Journal of counseling psychology. 1995;42(2):232.

102. Orpinas P, Frankowski R. The Aggression Scale: A self-report measure of aggressive behavior for young adolescents. The Journal of Early Adolescence. 2001;21(1):50-67.

103. Himelstein S, Saul S, Garcia-Romeu A. Does Mindfulness Meditation Increase Effectiveness of Substance Abuse Treatment with Incarcerated Youth? A Pilot Randomized Controlled Trial. Mindfulness. 2015;6(6):1472-80.

104. Cook CR. Student Externalizing Behavior Screener. School Psychology Quarterly. 2012.

105. Hartman K, Gresham FM, Byrd S. Student internalizing and externalizing behavior screeners: Evidence for reliability, validity, and usability in elementary schools. Behavioral disorders. 2017;42(3):108-18.

106. de la Villa Moral MM, Bernal AO. Análisis diferencial por niveles de edad de las actitudes hacia el consumo de sustancias psicoactivas en adolescentes españoles. Revista Interamericana de Psicología/Interamerican Journal of Psychology. 2005;39(3):325-38.

107. Funk JB, Elliott R, Urman ML, Flores GT, Mock RM. The Attitudes Towards Violence Scale: A Measure for Adolescents. Journal of Interpersonal Violence. 1999;14(11):1123-36.

108. Song L-y, Singh J, Singer M. The Youth Self-Report inventory: A study of its measurements fidelity. Psychological Assessment. 1994;6(3):236.

109. Doyle R, Mick E, Biederman J. Convergence Between the Achenbach Youth Self-Report and Structured Diagnostic Interview Diagnoses in ADHD and Non-ADHD Youth. The Journal of Nervous and Mental Disease. 2007;195(4).

110. Davis J, Matthews R. NEPSY-II Review. Journal of Psychoeducational Assessment. 2010;28:175-82.

111. Fan J, McCandliss BD, Sommer T, Raz A, Posner MI. Testing the efficiency and independence of attentional networks. Journal of cognitive neuroscience. 2002;14(3):340-7.

112. Johnson KA, Robertson IH, Barry E, Mulligan A, Dáibhis A, Daly M, et al. Impaired conflict resolution and alerting in children with ADHD: evidence from the Attention Network Task (ANT). Journal of Child Psychology and Psychiatry. 2008;49(12):1339-47.

113. Unsworth N, Heitz RP, Schrock JC, Engle RW. An automated version of the operation span task. Behavior Research Methods. 2005;37(3):498-505.

114. Wunsch K, Meier M, Ueberholz L, Strahler J, Kasten N. Acute psychosocial stress and working memory performance: the potential of physical activity to modulate cognitive functions in children. BMC Pediatrics. 2019;19(1):271.

115. Drollette ES, Scudder MR, Raine LB, Davis Moore R, Pontifex MB, Erickson KI, et al. The sexual dimorphic association of cardiorespiratory fitness to working memory in children. Developmental Science. 2016;19(1):90-108.

116. Greco LA, Lambert W, Baer RA. Psychological inflexibility in childhood and adolescence: development and evaluation of the Avoidance and Fusion Questionnaire for Youth. Psychological assessment. 2008;20(2):93.

117. Livheim F, Tengström A, Bond FW, Andersson G, Dahl J, Rosendahl I. Psychometric properties of the Avoidance and Fusion Questionnaire for Youth: A psychological measure of psychological inflexibility in youth. Journal of Contextual Behavioral Science. 2016;5(2):103-10.

118. Fergus TA, Valentiner DP, Gillen MJ, Hiraoka R, Twohig MP, Abramowitz JS, et al. Assessing psychological inflexibility: The psychometric properties of the Avoidance and Fusion Questionnaire for Youth in two adult samples. Psychological assessment. 2012;24(2):402.

119. Gioia GA, Isquith PK, Guy SC, Kenworthy L. Test review behavior rating inventory of executive function. Child Neuropsychology. 2000;6(3):235-8.

120. Isquith PK, Roth RM, Gioia G. Contribution of rating scales to the assessment of executive functions. Applied Neuropsychology: Child. 2013;2(2):125-32.

121. Corbett BA, Constantine LJ, Hendren R, Rocke D, Ozonoff S. Examining executive functioning in children with autism spectrum disorder, attention deficit hyperactivity disorder and typical development. Psychiatry research. 2009;166(2-3):210-22.

122. Riccio CA, Reynolds CR, Lowe PA. Clinical applications of continuous performance tests: Measuring attention and impulsive responding in children and adults: John Wiley & Sons Inc; 2001.

123. Corsi PM. Human memory and the medial temporal region of the brain. 1972.

124. Orsini A. Corsi's Block-Tapping Test: Standardization and Concurrent Validity with WISC—R for Children Aged 11 to 16. Perceptual and Motor Skills. 1994;79(3_suppl):1547-54.

125. Isaacs EB, Vargha-Khadem F. Differential course of development of spatial and verbal memory span: A normative study. British Journal of Developmental Psychology. 1989;7(4):377-80.

126. Hansen B. Decision Making Skills Scale. Tanglewood Research Evaluation, Substance Abuse and Mental Health …; 1996.

127. Wechsler D. WISC-IV administration manual. San Antonio, TX: The Psychological Corporation. 2003.

128. Wechsler D. Wechsler intelligence scale for children–Fourth Edition (WISC-IV). San Antonio, TX: The Psychological Corporation. 2003;3.

129. Zelazo PD. The Dimensional Change Card Sort (DCCS): a method of assessing executive function in children. Nature Protocols. 2006;1(1):297-301.

130. Doebel S, Zelazo PD. A meta-analysis of the Dimensional Change Card Sort: Implications for developmental theories and the measurement of executive function in children. Developmental Review. 2015;38:241-68.

131. Zelazo PD, Anderson JE, Richler J, Wallner-Allen K, Beaumont JL, Weintraub S. II. NIH Toolbox Cognition Battery (CB): Measuring executive function and attention. Monographs of the Society for Research in Child Development. 2013;78(4):16-33.

132. Simpson A, Riggs KJ. Conditions under which children experience inhibitory difficulty with a “button-press” go/no-go task. Journal of Experimental Child Psychology. 2006;94(1):18-26.

133. Wiebe SA, Sheffield TD, Espy KA. Separating the fish from the sharks: A longitudinal study of preschool response inhibition. Child development. 2012;83(4):1245-61.

134. Ponitz CC, McClelland MM, Matthews JS, Morrison FJ. A structured observation of behavioral self-regulation and its contribution to kindergarten outcomes. Dev Psychol. 2009;45(3):605.

135. McClelland MM, Cameron CE, Duncan R, Bowles RP, Acock AC, Miao A, et al. Predictors of early growth in academic achievement: The head-toes-knees-shoulders task. Frontiers in psychology. 2014;5:599.

136. Davidson MC, Amso D, Anderson LC, Diamond A. Development of cognitive control and executive functions from 4 to 13 years: Evidence from manipulations of memory, inhibition, and task switching. Neuropsychologia. 2006;44(11):2037-78.

137. Camerota M, Willoughby MT, Blair CB. Speed and accuracy on the Hearts and Flowers task interact to predict child outcomes. Psychological assessment. 2019;31(8):995.

138. Carlson S, Zelazo P. Minnesota executive function scale: Test manual. Saint Paul, MN: Reflection Sciences. 2014.

139. Prager EO, Sera MD, Carlson SM. Executive function and magnitude skills in preschool children. Journal of Experimental Child Psychology. 2016;147:126-39.

140. Diamond A, Taylor C. Development of an aspect of executive control: Development of the abilities to remember what I said and to "Do as I say, not as I do". Developmental psychobiology. 1996;29(4):315-34.

141. Lipsey MW, Nesbitt KT, Farran DC, Dong N, Fuhs MW, Wilson SJ. Learning-related cognitive self-regulation measures for prekindergarten children: a comparative evaluation of the educational relevance of selected measures. Journal of Educational Psychology. 2017;109(8):1084.

142. Thurstone L, Yela M. CARAS. Test de percepción de diferencias. Tea (11 ª ed) http://www web teaediciones com/ejemplos/caras-r% 20manual_2012 pdf. 2012.

143. Ricarte J, Ros L, Latorre J, Beltrán M. Mindfulness-based intervention in a rural primary school: Effects on attention, concentration and mood. International Journal of Cognitive Therapy. 2015;8(3):258-70.

144. Lawler JM, Esposito EA, Doyle CM, Gunnar MR. A Preliminary, Randomized-Controlled Trial of Mindfulness and Game-Based Executive Function Trainings to Promote Self-Regulation in Internationally-Adopted Children. Development and Psychopathology. 2019;31(4):1513-25.

145. Solanto MV, Abikoff H, Sonuga-Barke E, Schachar R, Logan GD, Wigal T, et al. The ecological validity of delay aversion and response inhibition as measures of impulsivity in AD/HD: a supplement to the NIMH multimodal treatment study of AD/HD. Journal of abnormal child psychology. 2001;29(3):215-28.

146. Stroop JR. Studies of interference in serial verbal reactions. Journal of Experimental Psychology: General. 1992;121(1):15.

147. Martínez-Loredo V, Fernández-Hermida JR, Carballo JL, Fernández-Artamendi S. Long-term reliability and stability of behavioral measures among adolescents: The Delay Discounting and Stroop tasks. Journal of Adolescence. 2017;58:33-9.

148. Robertson IH, Manly T, Andrade J, Baddeley BT, Yiend J. Oops!': performance correlates of everyday attentional failures in traumatic brain injured and normal subjects. Neuropsychologia. 1997;35(6):747-58.

149. Thomson P, Vijayakumar N, Johnson KA, Malpas CB, Sciberras E, Efron D, et al. Longitudinal Trajectories of Sustained Attention Development in Children and Adolescents with ADHD. Journal of Abnormal Child Psychology. 2020;48(12):1529-42.

150. Das J. Attention: Concept, tests and teacher ratings. Psychology and Developing Societies. 2002;14(2):241-60.

151. Manly T, Anderson V, Nimmo-Smith I, Turner A, Watson P, Robertson IH. The differential assessment of children's attention: The Test of Everyday Attention for Children (TEA-Ch), normative sample and ADHD performance. The Journal of Child Psychology and Psychiatry and Allied Disciplines. 2001;42(8):1065-81.

152. Bates ME, Lemay EP. The d2 Test of attention: construct validity and extensions in scoring techniques. Journal of the International Neuropsychological Society. 2004;10(3):392-400.

153. Budde H, Voelcker-Rehage C, Pietraßyk-Kendziorra S, Ribeiro P, Tidow G. Acute coordinative exercise improves attentional performance in adolescents. Neuroscience Letters. 2008;441(2):219-23.

154. Gualtieri CT, Johnson LG. Reliability and validity of a computerized neurocognitive test battery, CNS Vital Signs. Archives of Clinical Neuropsychology. 2006;21(7):623-43.

155. Swanson J, Schuck S, Mann M, Carlson C, Hartman K, Sergeant J, et al. Categorical and dimensional definitions and evaluations of symptoms of ADHD: The SNAP and the SWAN Ratings Scales [Draft]. Irvine, CA: UCL Child Development Center. 2005.

156. Swanson JM, Schuck S, Porter MM, Carlson C, Hartman CA, Sergeant JA, et al. Categorical and dimensional definitions and evaluations of symptoms of ADHD: history of the SNAP and the SWAN rating scales. The International journal of educational and psychological assessment. 2012;10(1):51.

157. Torrance EP. Norms technical manual: Torrance tests of creative thinking. Ginn Lexington, MA; 1974.

158. Kim KH. Can we trust creativity tests? A review of the Torrance Tests of Creative Thinking (TTCT). Creativity research journal. 2006;18(1):3-14.

159. Shallice T. Specific impairments of planning. Philosophical Transactions of the Royal Society of London B, Biological Sciences. 1982;298(1089):199-209.

160. Anderson P, Anderson V, Lajoie G. The tower of London test: Validation and standardization for pediatric populatons. The Clinical Neuropsychologist. 1996;10(1):54-65.

161. Delis DC, Kaplan E, Kramer JH. Delis-Kaplan executive function system. 2001.

162. Allen DN, Haderlie M, Kazakov D, Mayfield J. Construct and Criterion Validity of the Comprehensive Trail Making Test in Children and Adolescents with Traumatic Brain Injury. Child Neuropsychology. 2009;15(6):543-53.

163. Daneman M, Carpenter PA. Individual differences in working memory and reading. Journal of verbal learning and verbal behavior. 1980;19(4):450-66.

164. Bayliss DM, Jarrold C, Gunn DM, Baddeley AD. The complexities of complex span: explaining individual differences in working memory in children and adults. Journal of Experimental Psychology: General. 2003;132(1):71.

165. Kirchner WK. Age differences in short-term retention of rapidly changing information. Journal of experimental psychology. 1958;55(4):352.

166. Forns J, Esnaola M, López-Vicente M, Suades-González E, Alvarez-Pedrerol M, Julvez J, et al. The n-back Test and the Attentional Network Task as measures of child neuropsychological development in epidemiological studies. Neuropsychology. 2014;28(4):519.

167. Jaeggi SM, Buschkuehl M, Perrig WJ, Meier B. The concurrent validity of the N-back task as a working memory measure. Memory. 2010;18(4):394-412.

168. Barnes VA, Davis HC, Murzynowski JB, Treiber FA. Impact of meditation on resting and ambulatory blood pressure and heart rate in youth. Psychosomatic medicine. 2004 Nov 1;66(6):909-14.

169. Barnes VA, Pendergrast RA, Harshfield GA, Treiber FA. Impact of breathing awareness meditation on ambulatory blood pressure and sodium handling in prehypertensive African American adolescents. Ethnicity & disease. 2008;18(1):1.

170. Beattie M, Hankonen N, Salo G, Knittle K, Volanen SM. Applying behavioral theory to increase mindfulness practice among adolescents: an exploratory intervention study using a within-trial RCT design. Mindfulness. 2019 Feb;10(2):312-24.

171. Beattie MM, Konttinen HM, Volanen SM, Knittle KP, Hankonen NE. Social cognitions and mental health as predictors of adolescents' mindfulness practice. Mindfulness. 2020 May;11(5):1204-17.

172. Khoshkerdar P, Raeisi Z. The effect of mindfulness-based stress reduction on body image concerns of adolescent girls with dysfunctional eating attitudes. Australian Journal of Psychology. 2020 Mar 1;72(1):11-9.

173. Malboeuf-Hurtubise C, Léger-Goodes T, Mageau GA, et al. Philosophy for children and mindfulness during COVID-19: Results from a randomized cluster trial and impact on mental health in elementary school students. Progress in Neuro-Psychopharmacology and Biological Psychiatry. 2021 Apr 20;107:110260.

174. Salmoirago-Blotcher E, Druker S, Frisard C, et al. Integrating mindfulness training in school health education to promote healthy behaviors in adolescents: Feasibility and preliminary effects on exercise and dietary habits. Preventive medicine reports. 2018 Mar 1;9:92-5.

175. Lee RL, Lane SJ, Tang AC, Leung C, Kwok SW, Louie LH, Browne G, Chan SW. Effects of an unstructured free play and mindfulness intervention on wellbeing in kindergarten students. International Journal of Environmental Research and Public Health. 2020 Jan;17(15):5382.

176. Brown Wright L, Gregoski MJ, Tingen MS, et al. Impact of stress reduction interventions on hostility and ambulatory systolic blood pressure in African American adolescents. Journal of Black Psychology. 2011 May;37(2):210-33.

177. Yuan Y. Mindfulness training on the resilience of adolescents under the COVID-19 epidemic: A latent growth curve analysis. Personality and individual differences. 2021 Apr 1;172:110560.
